# Supplementary material for: Improving experience of medical abortion at home in a changing therapeutic, technological and regulatory landscape: a realist review
Source: BMJ Open. 2022 Nov 15;12(11):e066650. doi: 10.1136/bmjopen-2022-066650 (PMC9670095; doi:10.1136/bmjopen-2022-066650)
Supplement: Supplementary data [file bmjopen-2022-066650supp001.pdf]

Appendix 1: Databases searched and search terms

Ovid MEDLINE(R) and Epub Ahead of Print, In-Process & Other Non-Indexed Citations, Daily and Versions(R) 1946 to December 8, 2021; Embase Classic+Embase 1947 to 2021 December 08; APA PsycInfo 1806 to November Week 5 2021; APA PsycExtra 1908 to November 08, 2021; Global Health 1910 to 2021 Week 49; Social Policy and Practice 202010; CINAHL Plus, Web fo Science Core collection; Scopus (Elsevier); Cochrane Library.

Seach terms for Ovid: ((exp Abortion, Induced/) OR (abortion) OR (abortions) OR (pregnancy adj4 terminat*)) AND ((self care/) OR (self-management/) OR (Self Administration/) OR (Telemedicine/) OR (exp communications media/) OR (Internet-Based Intervention/) OR (Text Messaging/) OR (Outpatients/) OR (nurses/) OR (nursing staff/) OR (Nurse Midwives/) OR (Midwifery/) OR (Pharmacists/) OR (exp General Practice/) OR (Primary Health Care/) OR (Patient-Centred Care/) OR (self-manag*) OR (self-administer*) OR (self-induc*) OR (self-assess*) OR (self-sourc*) OR ("at home") OR ("home use") OR (home adj4 dosage) OR (home adj4 administ*) OR (home adj4 abortion) OR (telemedic*) OR (digit*) OR (online) OR (web) OR (website) OR (internet) OR (mobile) OR (sms) OR (text*) OR (messag*) OR (app) OR ("social media") OR (outpatient) OR (task-shar*) OR (task-shift*) OR (mid-level) OR (midlevel) OR (nurs*) OR (midwi*) OR (pharmacist) OR (pharmacy) OR (GP) OR (GPs) OR (general adj3 practic*) OR (non-speciali*) OR (non-clinical) OR (licen*) OR (qualif*) OR (decrim*) OR (woman-centered) OR (woman-centred) OR (women-centered) OR (women-centred) OR (patient-centered) OR (patient-centred) OR (gestation* adj5 limit*)) AND (("Patient Acceptance of Health Care"/) OR (Health Services Accessibility/) OR (exp patient satisfaction/ or patient preference/) OR (access*) OR (safe*) OR (accept*) OR (complicat*) OR (side effect*) OR (efficac*) OR (effect*) OR (feasib*) OR (prefer*) OR (satisf*) OR (feel*) or (autonom*) OR (delay*) OR (wait*) OR (experienc*) OR (uptake*) OR (time*) OR (distan*) OR (succe*) OR (impact) OR (opinion*) OR (best practice*) OR (capacity) OR (capab*) OR (competen*))

We searched Google (limited to the first 100 results) to identify grey literature and included known documents suggested by experts within the team and those identified through hand searches of the references of retrieved articles. Results from 01/01/2000 and 09/12/2022 that were peer reviewed research (journal articles and conference proceedings) theses or grey literature were included. We included papers where only a part of the investigation was relevant. We did not include books, book sections, cases, serials.
