# Supplementary material for: Improving experience of medical abortion at home in a changing therapeutic, technological and regulatory landscape: a realist review
Source: BMJ Open. 2022 Nov 15;12(11):e066650. doi: 10.1136/bmjopen-2022-066650 (PMC9670095; doi:10.1136/bmjopen-2022-066650)
Supplement: Supplementary data [file bmjopen-2022-066650supp002.pdf]

Appendix 2: Questions used to identify relevance

Five questions to operationalise a realist logic of analysis, adapted from Duddy et al (2021).

- Is there content that provides information on the context, mechanism or outcome of interventions? (CMO value)
- What do these data tell us about the context, mechanism or outcome of the interventions studies? (CMO outcomes)
- How should the CMO outcomes enable refinement of our initial programme theory? (refinement)
