# Supplementary material for: Improving experience of medical abortion at home in a changing therapeutic, technological and regulatory landscape: a realist review
Source: BMJ Open. 2022 Nov 15;12(11):e066650. doi: 10.1136/bmjopen-2022-066650 (PMC9670095; doi:10.1136/bmjopen-2022-066650)
Supplement: Supplementary data [file bmjopen-2022-066650supp003.pdf]

Appendix 3: Summary of papers included in the review

First Author	Date	Title	Source Type	Country	Setting	Participant Type	Number of Participants	Study Design	Is the paper relevant to the question?	Are the methods used appropriate?	Are the findings plausible?	Do conclusions support those of other studies?
Aiken, A. R. A	2018	Motivations and Experiences of People Seeking Medication Abortion Online in the United States	Original research paper	United States	Online	People seeking online medical abortion services	32	Qualitative interview study	Yes - paper explores motivations for considering self-managed abortion.	Yes - in-depth interviews were used to gather qualitative data on people's motivations and experiences of seeking online medical abortion.	Yes	Yes
Aiken, A.	2021b	Demand for self-managed online telemedicine abortion in eight European countries during the COVID-19 pandemic: a regression discontinuity analysis	Original research paper	Germany; Hungary; Italy; Malta; Netherlands; Northern Ireland; Portugal; Great Britain	Online	N/A	3915 requests for self-managed abortion to Women on Web (WoW)	Cross-sectional comparative analysis of requests for self-managed abortion pre- and post the Covid-19 pandemic	Yes - paper assesses whether the COVID-19 pandemic increased demand for self-managed medical abortion provided through online services and suggests reasons for trends in demand for online accessed abortion during the pandemic.	Yes - request rates in 8 countries are compared using regression discontinuity both before and after lockdown measures.	Yes	Yes
Atay	2021	Why women choose at-home abortion via teleconsultation in France: drivers of telemedicine abortion during and beyond the	Original research paper	France	Online	People accessing online medical abortion services	140	Mixed Methods (Cross-sectional survey of consultation data and email content analysis)	Highly - the paper explores drivers of at home, online supported, medical abortion during the pandemic and beyond.	Yes - the findings are based on appropriate analysis of consultations and emails extracted from an online medical abortion service to reveal the motivations and preferences of women seeking at home abortion.	Yes	Yes

		COVID-19 pandemic.										
Boydell	2021	Women's experiences of a telemedicine abortion service (up to 12 weeks) implemented during the coronavirus (COVID-19) pandemic: a qualitative evaluation	Original research paper	Scotland	Online	People accessing online medical abortion services	20	Qualitative interview study	Highly - paper explores women's experiences of accessing online medical abortion services.	Yes - the findings are based on appropriate use of qualitative interviews to explore women's experiences of telephone consultation; remote support; views on no pre-abortion ultrasound; and self-administration of abortion medications at home.	Yes	Yes
Ehrenreich	2019	Spatial dimensions of telemedicine and abortion access: a qualitative study of women's experiences	Original research paper	Utah, USA	Online	People accessing online medical abortion services	20	Qualitative interview study	Yes - the paper explores women's experiences of using online services for the first mandatory step when accessing medical abortion in Utah, an 'information visit'.	Yes - through in-depth interviews thoroughly explore experiences of using online services for information visits to fulfil abortion requirements.	Yes	Yes
Endler	2019	Telemedicine for medical abortion: a systematic review	Review	N/A	N/A	N/A	N/A	Systematic Review	Yes - the paper assesses the success rate, safety, and acceptability for women and providers of online medical abortion services.	Yes - a systematic review assessing a broad and significant number of relevant studies.	Yes	Yes
Erlank	2021	Acceptability of no-test medical abortion provided via telemedicine during Covid-19: analysis of patient-reported outcomes	Original research paper	United Kingdom	Online	People accessing online medical abortion services	1243	Qualitative survey study	Yes - the study reports patient-reported outcome measures assessing the quality of consultation, access to medicine after consultation, ability to manage the process at home and overall patient satisfaction.	Yes – early medical abortion patients invited to opt-in to a follow-up call post-procedure to answer clinical and satisfaction questions to measure acceptability of the service.	Yes	Yes

Eshre Capri Workshop Group	2017	Induced abortion	Workshop	N/A	N/A	N/A	N/A	N/A	Moderately - This paper presents commentary on induced abortion gathered at a workshop of experts in the field. The commentary included discussion of at home medical abortion with reference to online support.	N/A	N/A	Yes
Finch	2019	Impact of self-administration of misoprostol for early medical abortion: a prospective cohort study	Original research paper	Scotland	Home	People accessing abortion services both at home and at a facility	2430	Prospective observational study	Yes - the study explores the impact of Scotland's legalisation on home use of misoprostol for the purpose of early medical abortion on uptake and success rate, and on the provision of effective contraception on discharge.	Yes - prospective observational study followed the outcomes of two cohorts who received their abortion care, before and after the introduction of home use of misoprostol.	Yes	Yes
Fix	2020	At-home telemedicine for medical abortion in Australia: a qualitative study of patient experiences and recommendations	Original research paper	Australia	Online	People accessing online medical abortion services	24	Qualitative interview study	Highly - the study explores people's experiences obtaining a medical abortion through an online service and discusses people's reasons for choosing an online service.	Yes - in-depth interviews appropriately captures the experiences of people using the at-home telemedicine service.	Yes.	Yes
Gill	2019	Feasibility and Acceptability of a Mobile Technology Intervention to Support Postabortion Care in British	Original research paper	Canada	Abortion care facility	People attending an abortion care facility	58	Mixed-methods formative study (cross-sectional survey and semi-structured interviews)	Moderately - study aims to understand how people at surgical abortion clinics utilise their mobile phones to access health care information and their preferences for a mobile intervention	Yes - mixed-methods study appropriately explores a variety of aspects relevant to technology usage during abortion process.	Yes	Yes

		Columbia: Phase 1							that supports follow-up care.			
Gill & Norman	2018	Telemedicine and medical abortion: dispelling safety myths, with facts	Editorial	N/A	N/A	N/A	N/A	Editorial commentary on recent research regarding online medical abortion services	Moderately - This paper presents commentary on recent research regarding online medical abortion services.	N/A	N/A	Yes
Goldman	2021	Transcutaneous Electrical Nerve Stimulation to Reduce Pain With Medication Abortion: A Randomized Controlled Trial	Original research paper	N/A	Abortion care facility	People undergoing medical abortion.	251	Randomised controlled trial (RCT)	Yes - evaluates whether high-frequency transcutaneous electrical nerve stimulation reduces experiences of pain during medical abortion.	Yes – randomized control trial shows that participants who received high-frequency transcutaneous electrical nerve stimulation after medical abortion had significantly lower posttreatment pain scored compared to those that received a sham procedure.	Yes	Yes
Hamoda	2005	The acceptability of home medical abortion to women in UK settings	Original research paper	England and Scotland	Home	People seeking hospital based abortion care	553	Qualitative survey study	Highly - the study explores the acceptability of home medical abortion to people UK settings including managing pain and bleeding at home.	Yes - self-complete questionnaires adequately measure people's views on the acceptability of medical abortion at home.	Yes	Yes
Harden	2021	Women's experiences of self-administration of misoprostol at home as part of early medical abortion: a qualitative evaluation	Original research paper	Scotland	Home	People that had recently undergone early medical abortion	20	Qualitative interview study	Highly - the study explores the experiences of people who accessed at home medical abortion and reported benefits of at home administration including flexibility, privacy and comfort.	Yes - qualitative data is collected exploring what people desire and valued during the process.	Yes	Yes
Heath	2019	A comparison of termination of pregnancy procedures: Patient choice,	Original research paper	Sweden	Abortion care facility	People requesting an abortion		A mixed-method prospective comparative study	Yes	Yes	Yes	Yes

		emotional impact and satisfaction with care										
Hedqvist	2016	Women's experiences of having an early medical abortion at home	Original research paper	Sweden	Home	People accessing medical abortion at home	119	Cross-sectional (semi-structured telephone interviews)	Highly - paper investigates people's experiences of having a medical abortion at home including experiences of pain and bleeding.	Yes - descriptive and comparative design enable an in-depth and balanced conclusion. Study also investigates differences between groups of people.	Yes	Yes
Hoggart and Berer	2021	Making the case for supported self-managed medical abortion as an option for the future	Editorial	N/A	Online	N/A	N/A	Editorial commentary on recent research regarding online medical abortion services	Moderately – this paper reviews recent research on online medical abortion services highlighting benefits and pointing out need for further development of services, suggesting a need for access to 24 hours support.	N/A	N/A	Yes
Iyer	2021	Preferences for contraceptive counselling and access among abortion patients at an independent clinic in Texas	Original research paper	USA	Abortion care facility	People attending an abortion care facility	181	Cross-sectional survey study	Yes - study explores preferences for contraceptive counselling and access to contraception.	Yes - self-administered surveys used as an appropriate method considering legally restrictive setting and associated stigma.	Yes	Yes
Kavanagh	2011	Patients' attitudes and experiences related to receiving contraception during abortion care	Original research paper	USA	Abortion care facility	People accessing abortion at an abortion care facility	542	Cross-sectional survey study	Yes - study documents attitudes towards contraceptive services and identifies participants characteristics associated with desire for contraception and interest in LARC.	Yes - appropriate methods for exploring attitudes and characteristics associated with specific needs relevant to contraceptive use.	Yes	Yes
Kero	2010	Home abortion - experiences of	Original research paper	Sweden	Home	Couples - where the male partner had been	23 couples	Qualitative interview study	Moderately - the paper explores the male partner's experience of being	Yes - qualitative method ensures high detailed analysis in experiences of	Yes	Yes

		male involvement				present when their female partner had had an at home medical abortion			present and supporting their partner during an induced home abortion.	both the pregnant woman and her partner.		
Killinger	2022	Why women choose abortion through telemedicine outside the formal health sector in Germany: a mixed-methods study	Original research paper	Germany	Online	People accessing an online medical abortion service	1090	Cross-sectional study analysing data from online consultations	Yes - the paper aims to understand the motivations and barriers to access for people who choose online abortion services outside of the formal health sector.	Yes - a cross-sectional study of data contained in online consultations and a content analysis of over 100 email texts.	Yes	Yes
Levine and Cameron	2009	Women's preferences for method of abortion and management of miscarriage	Original research paper	United Kingdom	Abortion care facility	People undergoing medical abortion, surgical abortion and surgical management of miscarriage	148	Cross-sectional survey study	Yes - the study explores views on medical treatment at home, and surgery under local anaesthesia, to determine whether new services should be developed.	Moderately - small sample size of self-administered anonymous questionnaires.	Yes	Yes
Loeber	2016	Contraceptive counselling for women with multiple unintended pregnancies: the abortion client's perspective	Original research paper	Netherlands	Abortion care facility	Survey – People attending an abortion care facility<br><br>Interviews – People attending an abortion care facility that had had more than three unintended pregnancies	212	Mixed methods (quantitative survey and semi-structured interviews)	Yes - study explores views on contraception use after induced abortion and post-abortion contraceptive counselling	Yes - mixed method approach appropriately explores experiences of post-abortion contraception and contraception counselling both in-depth and across a larger sample.	Yes	Yes

Lohr	2018	Telephone or integrated contraception counselling before abortion: impact on method choice and receipt	Original research paper	United Kingdom	Abortion care facility	People who had accessed abortion at an abortion care facility	18,573	Cross-sectional study of de-identified data	Yes - paper compares the characteristics of people who chose contraception counselling either over the telephone and separate from abortion consultation or face-to-face and integrated into consultation.	Yes - very large sample size which thoroughly explores demographic characteristics and contraceptive method choice.	Yes	Yes
Lokeland	2014	Medical abortion with mifepristone and home administration of misprostol up to 63 days' gestation	Original research paper	Norway	Abortion care facility	People seeking an at home medical abortion before 63 days'	1,018	Observational study	Yes - paper evaluates the acceptability of medical abortion at home, including pain and bleeding.	Yes – a large sample size of people experiencing at home abortion with follow up telephone recorded to asses bleeding, pain and acceptability.	Yes	Yes
Low	2021	Women's experiences of self-referral to an abortion service: qualitative study	Original research paper	Scotland	Abortion care service	People attending an abortion care service	21	Qualitative interview study	Yes - this study evaluates a self-referral service to abortion services by investigating its impact on people's experiences of the referral process.	Yes - semi-structured interviews to focus on people's experiences of the referral process.	Yes	Yes
Makenzius	2012	Autonomy and dependence - experiences of home abortion, contraception and prevention	Original research paper	Sweden	Home	People who have experienced an at-home abortion	37	Qualitative interview study	Yes - study explores people's experiences and needs related to care in the context of a home abortion.	Yes – interviews capture patients at-home abortion experiences and needs which fall under themes of autonomy (choice of at-home abortion, increase of privacy and control) and dependence (desire to be treated with empathy by health care workers and receive adequate information).	Yes	Yes
Matulich	2014	Understanding women's desires for contraceptive counselling at the time of	Original research paper	USA	Abortion care facility	People receiving surgical abortion at an abortion care facility	199	Qualitative survey study	Yes - paper investigates whether or not people presenting for a first-trimester surgical abortion want to	Yes - survey obtained demographic information and inquired about desire for contraceptive counselling.	Yes	Yes

		first-trimester surgical abortion							discuss contraception on the day of their procedure.			
Norman	2014	Access to Complex Abortion Care Service and Planning Improved through a Toll-Free Telephone Resource Line	Original research paper	Canada	Abortion care facility	N/A	N/A	Review of service provision model	Yes - study presents a review of a service provision model which has provided improved access to abortion care through a toll-free telephone abortion access and counselling service.	Yes - a descriptive analysis appropriately reports on the development and delivery of this service.	Yes	Yes
Oppegaard	2015	Clinical follow-up compared with self-assessment of outcome after medical abortion: a multicentre, non-inferiority, randomised, controlled trial	Original research paper	Austria; Finland; Norway; Sweden	Abortion care services	People requesting medical abortion	924	Randomised, controlled, non-inferiority trial	Yes - study compares clinical assessment with self-assessment of abortion outcome.	Yes - assigned people in a 1:1 ratio to attend routine clinical follow-up or to self-assess outcome at home with a semi-quantitative urine human chorionic gonadotropin test 1-3 weeks after abortion.	Yes	Yes
Pohjoranta	2018	Predicting poor compliance with follow-up and intrauterine contraception services after medical termination of pregnancy	Original research paper	Finland	Abortion care facility	People undergoing a medical abortion	605	RCT	Moderately - paper assesses factors associated with non-compliance with post-abortion services and evaluates differences in rates of attendance and intrauterine device insertion.	Yes – the study compared the intervention group booked to have IUD insertion 1-4 weeks after medical abortion and women in control group were advised to contact their PHC for follow-up and IUD insertion.	Yes	Yes
Pohjoranta	2020	Early provision of intrauterine contraception as part of abortion care - 5-year results of a randomised controlled trial	Original research paper	Finland	Abortion care facility	People undergoing an induced abortion	748	RCT	Moderately - paper investigates whether the incidence of subsequent termination of pregnancy can be reduced by providing IUD as part of the abortion service.	Yes – RCT assesses the effectiveness of early comprehensive provision of IUD after induced abortion. Intervention group provided with an IUD during surgical abortion and women in control group advised to	Yes	Yes

Baraitser P, et al. BMJ Open 2022; 12:e066650. doi: 10.1136/bmjopen-2022-066650

										contact primary healthcare for follow-up and IUD insertion.		
Powell-Jackson	2010	Benefits of using a digital video disk for providing information about abortion to women requesting termination of pregnancy	Original research paper	Scotland	Abortion care facility	People attending an abortion care facility and facility staff	226	Comparative cross-sectional survey design	Yes - paper examines the benefits of using a DVD for providing information about abortion.	Yes - questionnaires are used to appropriately measure people's satisfaction with the information received through the DVD and what more/less they would want it to include.	Yes	Yes
Purcell	2017	Self-managment of first trimester medical termination of pregnancy: a qualitative study of women's experiences	Original research paper	Scotland	Abortion care facility	People who are administered a medical abortion at a facility and return home to complete termination	44	Qualitative interview study	Yes - explores people's experiences of returning home to complete medical abortion, including self-monitoring of treatment process.	Yes - in-depth interviews to assess people's experiences in detail.	Yes	Yes
Rafferty & Longbons	2021	#AbortionChangesYou: A Case Study to Understand the Communicative Tensions in Women's Medication Abortion Narratives	Original research paper	United States	Online	People who have had a medical abortion	98 blog posts	Case study	Yes - study analyses people's narratives after having had a medication abortion though blogs posted on a website - Abortion Changes You.	Yes - using a case study approach enabled the study to explore multiple perspectives rooted in specific contexts.	Yes	Yes
Raymond	2018	Low-sensitivity urine pregnancy testing to assess medical abortion outcome: A systematic review	Review	N/A	N/A	N/A	N/A	Systematic review	Yes - the study explores data on the accuracy and acceptability of a strategy for identifying ongoing pregnancy after medical abortion treatment using a low-sensitivity pregnancy test (LSPT).	Moderately - A more comprehensive search across other databases could strengthen study.	Yes	Yes

Raymond	2017	Self-assessment of medical abortion outcome using symptoms and home pregnancy testing	Original research paper	United States	Abortion care facility	People undergoing medical abortion	343	Intervention study	Highly - study evaluates compliance with a strategy to enable medical abortion patients to assess treatment outcome on their own and decide whether to seek clinical follow-up.	Yes - appropriate methodology to clinically assess women's compliance to using a pregnancy test after medical abortion and women's ability to self-assess treatment outcome.	Yes	Yes
Reynolds-Wright	2020	Pain management for medical abortion before 14 weeks' gestation	Review	N/A	N/A	N/A	N/A	Systematic review	Yes - paper reviews pain relief regimens for the management of medical abortion.	Yes - wide variety of databases, conference abstracts and organisations systematically searched and analysed.	Yes	No
Reynolds-Wright	2021	Telemedicine medical abortion at home under 12 weeks' gestation: a prospective observational cohort study during the COVID-19 pandemic	Original research paper	Scotland	Online/Home	People choosing online medical abortion services	663	Prospective cohort study	Yes - the paper determines the efficacy of an online at-home medical abortion service, noting any complications after treatment and acceptability of care.	Yes - large sample size. Questionnaires and hospital database analyses allows for in-depth review of efficacy, complications and acceptability.	Yes	Yes
Robson	2009	Randomised preference trial of medical versus surgical termination of pregnancy less than 14 weeks' gestation (TOPS)	Original research paper	United Kingdom	Abortion care facility	People undergoing induced abortion and people attending a contraceptive and sexual health clinic	1,877	Randomised preference trial and economic evaluation with qualitative sub study	Yes - the paper explores the acceptability, efficacy and costs of medical abortion compared with surgical abortion and aims to understand people's decision-making processes and experiences.	Yes – participants are either randomly assigned a procedure type (with consent to random allocation) or choose their procedure type to evaluate whether in people without prior preference, acceptability of medical and surgical abortion is the same. A qualitative sub study explores people's preferences and decision making.	Yes	Moderately
Rowlands & Wale	2020	A Constructivist	Constructivist Vision	N/A	N/A	N/A	N/A	Constructivist vision	Moderately - This paper provides	N/A	N/A	Yes

		Vision of the First-Trimester Abortion Experience						commentary on how a high-quality abortion experience might be achieved in the first trimester if regulatory laws were dismissed.	commentary on recent research on induced abortion in the first trimester within the framework of a future vision of state obligation to provide high quality abortion care. This paper also provides an evidence-based set of ingredients that facilitate a positive high-quality abortion experience.			
Schmidt-Hansen	2020	Follow-up strategies to confirm the success of medical abortion of pregnancies up to 10 weeks' gestation: a systematic review with meta-analyses	Review	N/A	N/A	N/A	N/A	Systematic review	Yes - the review compares the effectiveness, safety, and acceptability of in-clinic and remote/self-assessment for confirming the success of medical abortion.	Yes - systematic review including RCT's from 2000 onward to ensure a broad range of studies captured. Quality of evidence compromised by small event rates, lack of blinding, and high attrition rates.	Yes	Yes
Sherman	2017	Providing experiential information on early medical abortion: a qualitative evaluation of an animated personal account, *Lara's Story*	Original research paper	Scotland	Abortion care facility	People who have had a medical abortion	13	Qualitative interview study	Yes - the study evaluates the views on an animated film, and its potential usefulness in providing information on medical abortion.	Yes - the use of interviews allows people to report their full experience and opinion in detail, also providing feedback or criticism.	Yes	Yes
Smith	2019	Current barriers, facilitators and future improvements to advance	Original research paper	Edinburgh, Scotland	Abortion care facility	People seeking induced abortion	154	Cross-sectional survey study	Yes - the study explores current barriers access to care, and what future improvements should	Yes - self-administered anonymous questionnaires to capture experiences.	Yes	Yes

		quality of abortion care: views of women							be implemented to abortion care services			
Smith	2017a	Women's views and experiences of a mobile phone-based intervention to support post-abortion contraception in Cambodia	Original research paper	Cambodia	Abortion care facility	People who had received a mobile phone based post-abortion contraception counselling service.	15	Qualitative interview study	Yes - the study assesses views and experiences of receiving the MObile Technology for Improved Family Planning (MOTIF) intervention, which aims to increase post-abortion contraception.	Yes - semi-structured interviews to capture in-depth data. Small sample size may be a limitation.	Yes	Yes
Smith	2017b	Process evaluation of a mobile phone-based intervention to support post-abortion contraception in Cambodia	Original research paper	Cambodia	Abortion care facility	People who had received a mobile phone based post-abortion contraception counselling service.	249	Process evaluation	Yes - the study assesses participants' interaction with the MOTIF intervention.	Yes - thorough process evaluation to assess associations with the intervention and post-abortion contraception use.	Yes	Yes
Upadhyay, U. D.	2021	Safety and Efficacy of Telehealth Medication Abortions in the US During the COVID-19 Pandemic	Original research paper	USA	Online	People accessing online medical abortion	141	Retrospective cohort study	Yes - the study assesses the safety and efficacy outcomes of a online medical abortion model.	Yes – appropriate methodology for assessing an online medical abortion service however the small sample size with some loss to follow-up, and thus some adverse events and ongoing pregnancies may have been undetected.	Yes	Yes
Whitehouse	2021	*It's a small bit of advice, but actually on the day, made such a difference...*: perceptions of quality in abortion care in England and Wales	Original research paper	England; Wales	Abortion care facility	People who have had an abortion at a facility withing the last 6 months	24	Qualitative interview study	Yes - the paper explores participants' experiences and views on abortion quality of care.	Yes - interviews are an appropriate methodology to assess experiences.	Yes	Yes

Wieβe	2008	Access to Abortion: What Women Want From Abortion Services	Original research paper	Canada	Abortion care facility	People seeking an abortion	441	Qualitative survey and interview study	Moderately - the paper identifies barriers to access for women seeking induced abortion at a facility.	Yes - questionnaire accurately explores demographic information, perceived barriers to access, and what women desire from abortion services. Interviews captured in-depth analysis to access questions.	Yes	Yes
