# Supplementary material for: Improving experience of medical abortion at home in a changing therapeutic, technological and regulatory landscape: a realist review
Source: BMJ Open. 2022 Nov 15;12(11):e066650. doi: 10.1136/bmjopen-2022-066650 (PMC9670095; doi:10.1136/bmjopen-2022-066650)
Supplement: Author's manuscript [file bmjopen-2022-066650.draft_revisions.pdf]

BMJ Open is committed to open peer review. As part of this commitment we make the peer review history of every article we publish publicly available.

When an article is published we post the peer reviewers' comments and the authors' responses online. We also post the versions of the paper that were used during peer review. These are the versions that the peer review comments apply to.

The versions of the paper that follow are the versions that were submitted during the peer review process. They are not the versions of record or the final published versions. They should not be cited or distributed as the published version of this manuscript.

BMJ Open is an open access journal and the full, final, typeset and author-corrected version of record of the manuscript is available on our site with no access controls, subscription charges or pay-per-view fees (http://bmjopen.bmj.com).

If you have any questions on BMJ Open's open peer review process please email info.bmjopen@bmj.com

BMJ Open

Improving experience of medical abortion at home in a changing therapeutic, technological and regulatory landscape: a realist review

Journal:	*BMJ Open*
Manuscript ID	bmjopen-2022-066650
Article Type:	Original research
Date Submitted by the Author:	21-Jul-2022
Complete List of Authors:	Baraitser, Paula; King's College London School of Population Health and Environmental Sciences<br>Free, Caroline; London School of Hygiene & Tropical Medicine<br>Norman, Wendy; The University of British Columbia, Dept of Family Practice; London School of Hygiene and Tropical Medicine Faculty of Public Health and Policy, Public Health, Environments & Society<br>Lewandowska, Maria; London School of Hygiene and Tropical Medicine Faculty of Public Health and Policy<br>Meiksin, Rebecca; London School of Hygiene and Tropical Medicine Faculty of Public Health and Policy<br>Palmer, Melissa; London School of Hygiene and Tropical Medicine Faculty of Public Health and Policy<br>Scott , Rachel ; London School of Hygiene and Tropical Medicine Faculty of Public Health and Policy<br>French, Rebecca ; London School of Hygiene and Tropical Medicine Faculty of Public Health and Policy<br>Wellings, Kaye; London School of Hygiene & Tropical Medicine<br>Ivory, Alice; London School of Hygiene & Tropical Medicine, DPH<br>Wong, Geoff; Oxford University, Nuffield Department of Primary Care Health Sciences
Keywords:	Telemedicine < BIOTECHNOLOGY & BIOINFORMATICS, SEXUAL MEDICINE, REPRODUCTIVE MEDICINE

SCHOLARONE™
Manuscripts

I, the Submitting Author has the right to grant and does grant on behalf of all authors of the Work (as defined in the below author licence), an exclusive licence and/or a non-exclusive licence for contributions from authors who are: i) UK Crown employees; ii) where BMJ has agreed a CC-BY licence shall apply, and/or iii) in accordance with the terms applicable for US Federal Government officers or employees acting as part of their official duties; on a worldwide, perpetual, irrevocable, royalty-free basis to BMJ Publishing Group Ltd ("BMJ") its licensees and where the relevant Journal is co-owned by BMJ to the co-owners of the Journal, to publish the Work in this journal and any other BMJ products and to exploit all rights, as set out in our licence.

The Submitting Author accepts and understands that any supply made under these terms is made by BMJ to the Submitting Author unless you are acting as an employee on behalf of your employer or a postgraduate student of an affiliated institution which is paying any applicable article publishing charge ("APC") for Open Access articles. Where the Submitting Author wishes to make the Work available on an Open Access basis (and intends to pay the relevant APC), the terms of reuse of such Open Access shall be governed by a Creative Commons licence – details of these licences and which Creative Commons licence will apply to this Work are set out in our licence referred to above.

Other than as permitted in any relevant BMJ Author's Self Archiving Policies, I confirm this Work has not been accepted for publication elsewhere, is not being considered for publication elsewhere and does not duplicate material already published. I confirm all authors consent to publication of this Work and authorise the granting of this licence.

For peer review only

Improving experience of medical abortion at home in a changing therapeutic, technological and regulatory landscape: a realist review

Authors

Paula Baraitser[1], Caroline Free[2], Wendy Norman[3,4], Maria Lewandowska[4*], Rebecca Meiksin[4], Melissa Palmer[4], Rachel Scott[4], Rebecca French[4], Kaye Wellings[4], Alice Ivory[2], Geoff Wong[5], The SACHA study team.

[1] School of Population Health & Environmental Sciences, King's College London, London, UK
[2] Faculty of Epidemiology and Population Health, London School of Hygiene & Tropical Medicine
[3] Department of Family Practice, University of British Columbia, Vancouver, British Columbia, Canada.
[4] Faculty of Public Health and Policy, London School of Hygiene & Tropical Medicine
[5] Nuffield Department of Primary Care Health Sciences, University of Oxford, Oxford, UK

*Corresponding author:
Maria Lewandowska, maria.lewandowska@lshtm.ac.uk 020 7927 2761
London School of Hygiene and Tropical Medicine 15-17 Tavistock Place, WC1H 9SH

Paula Baraister paula.baraitser@kcl.ac.uk
Caroline Free caroline.free@lshtm.ac.uk
Wendy Norman wendy.norman@lshtm.ac.uk
Maria Lewandowska maria.lewandowska@lshtm.ac.uk
Rebecca Meiksin rebecca.meiksin@lshtm.ac.uk
Melissa Palmer melissa.palmer@lshtm.ac.uk
Rachel Scott Rachel.scott@lshtm.ac.uk
Rebecca French rebecca.french@lshtm.ac.uk
Kaye Wellings kaye.wellings@lshtm.ac.uk
Alice Ivory alice.ivory@lshtm.ac.uk
Geoffrey Wong Geoffrey.wong@phc.ox.ac.uk

Target journal

BMJ Open

Word count

4199

Keywords

Abortion, telemedicine, reproductive health, self-care

For peer review only

For peer review only - http://bmjopen.bmj.com/site/about/guidelines.xhtml

Abstract

Objective:

To inform UK service development to support medical abortion at home, appropriate for person and context.

Design:

Realist review

Setting/participants:

Peer-reviewed literature from 01/01/2000 to 09/12/2021, describing interventions or models of abortion care. Participants included people seeking or having had an abortion.

Interventions:

Interventions and new models of abortion care relevant to the UK.

Outcome measures:

Causal explanations, in the form of context-mechanism-outcome configurations, to test and develop our realist program theory.

Results:

We identified 12,401 abstracts, selecting 944 for full text assessment. Our final review included 50 papers. Medical abortion at home is safe, effective and acceptable to most, but clinical pathways and user experience are variable and a minority would not choose this method again. Having a choice of abortion location remains essential, as some people are unable to have a medical abortion at home. Choice of place of abortion (home or clinical setting) was influenced by service factors (appointment number, timing, and wait-times), personal responsibilities (caring/work commitments), geography (travel time/distance), relationships (need for secrecy) and desire for awareness/involvement in the process. We found experiences could be improved by offering: an option for self-referral through a telemedicine consultation, realistic information on range of experiences, opportunities to personalise the process, improved pain relief, and choice of when and how to discuss contraception.

Conclusions

Acknowledging the work done by patients when moving medical abortion care from clinic to home is important. Patients may benefit from support to: prepare a space, manage privacy and work/caring obligations, decide when/how to take medications, understand what is normal, assess experience and decide when and how to ask for help. The transition of this complex intervention when delivered outside healthcare environments could be supported by strategies that reduce surprise or anxiety, enabling preparation and a sense of control.

Strenghts and limitations

- Strengths: systematic and transparent approach to the realist review, which was conducted in accordance with the RAMSES standards (27); Authorship team represents a variety of clinical and academic backgrounds
- Limitations: analysis on publicly accessible literature, located through recognised research databases and Google; there were gaps in the evidence that we found and we have highlighted these in our conclusions.

Author contributions

RF, KW, CF & PB conceptualised the study. GW was responsible for the methodology. RF, KW, CF, PB, GW, RM, MP, RS and ML wrote the protocol. ML registered the review in the Prospero

For peer review only - http://bmjopen.bmj.com/site/about/guidelines.xhtml

system (ID CRD42021225307) and conducted the searches. All authors screened the papers. WN conducted preliminary data extraction. All authors participated in the data analysis. AI composed the tables. PB, CF, WN and GW wrote the first draft. All authors contributed to the final manuscript. This paper reports on data collected as part of the SACHA Study: Shaping Abortion for Change, funded by the National Institute of Health Research. (www.lshtm.ac.uk/sacha) Collaborators of the SACHA Study provided feedback on all stages of the study.

Funding statement
This paper is a part of the SACHA Study, funded by the National Institute of Health Research, award NIHR129529

Conflicts of interests
None

Protocol
Protocol can be found in Propsero under ID CRD42021225307

Data availability statement
Data extracted from studies and used for analysis can be requested from the corresponding authors.

Patient and Public Involvement
The whole SACHA Study, including the realist review, was consulted by an Advisory Group with PPI members, who helped in developing the research questions and methodology. The development of the research question and outcome measures were entirely informed by patients' priorities. Patient representatives were not involved in the conduct, analysis or interpretation of the results. The results of the study will be disseminated to the public and to patients who were involved in other components of the study through online newsletters, press releases, informational videos posted online and through other activities informed by the consultations with the Advisory Group.

Ethical approval
No ethical approval was required for review of evidence.

For peer review only

For peer review only - http://bmjopen.bmj.com/site/about/guidelines.xhtml

Background

Changes in the therapeutic, technological and regulatory landscape are impacting abortion care and changing access to medical abortion at home. Medical abortion requires two medications, mifepristone (a competitive progestogen receptor antagonist) and the synthetic prostaglandin, misoprostol. Since mifepristone was first licenced for use in Great Britain in 1991, the proportion of all abortions that are medical abortions has increased steadily with a more recent shift towards medical abortion at home, where both the mifepristone and misoprostol or the misoprostol alone are taken at home. During the COVID 19 pandemic these trends were accelerated and in 2020, 85% of all abortions completed in England and Wales, and 88% of those in Scotland, were medical abortions at home[1,2] . These developments reflect similar changes in Australia, Canada and Europe[3-7].

Medical abortion at home is safe, effective and acceptable and clinical guidance on some elements, such as doses of abortion medications, are consistent[7-21]. However, significant variation in other elements of clinical pathways remain as providers test new approaches to providing information, support, pain relief and follow up[22]. It is important to note that a minority (17 – 34%) of people who participate in research on medical abortion at home would not choose this method if having an abortion again[12,23-25.] We are interested in the evidence to inform optimal configuration of medical abortion services at home and particularly in the differences in experience between populations and contexts.

Terminology and settings

Our findings relate to settings where abortion is legal and where there is a choice of abortion method and setting. Recommendations may be different in settings where abortion is illegal, where mifepristone is not available, where medication combinations or doses are used that are nt evidence based or where surgical options are not available or not safe. Women and people with other gender identities may have abortions. We have used the terms ‘people who have an abortion’ or ‘people’ to describe our population of interest.

Objectives

- To synthesise evidence on user experience of medical abortion at home
- To develop a realist programme theory to explain what interventions improve user experience for whom and in what context.
- To use this programme theory to develop recommendations for service providers and those having medical abortions at home

Review question

- What support, for whom, and in what circumstances could improve the experience of people who have medical abortion at home?

For peer review only - http://bmjopen.bmj.com/site/about/guidelines.xhtml

Methods

The SACHA study brings together over 20 researchers from seven countries to create an evidence base to inform health service configuration for abortion provision in the UK (https://www.lshtm.ac.uk/research/centres-projects-groups/sacha#welcome). This review is part of this programme of work and draws on the expertise of the team.

We conceptualised medical abortion as a complex process that has context-sensitive outcomes which may be variably delivered and experienced. A realist approach enabled us to both account for and explain the influence of context on this variation[26]. Our protocol was registered with Prospero https://www.crd.york.ac.uk/prospero/display_record.php?RecordID=225307 and we followed the steps which are commonly used in realist reviews[27,28]- location of existing theories, evidence search and document selection, data extraction, data synthesis and development of a refined programme theory.

We used the PRISMA checklist when writing our report.[1]

Location of existing theories

A realist approach to understanding interventions (in our case home medical abortion) proposes that any intervention is underpinned by one or more theories (that may be implicit or explicit) in the design of the intervention. This theory sets out how the designers anticipate their intervention will work. In a realist review this understanding is captured via an initial programme theory. Our initial programme theory was developed with input from the SACHA team. We charted the stages of home medical abortion from a user perspective to understand what type of support might be required from the health care system at each stage (figure 1). These stages within a user journey were used to structure our analysis.

Figure 1 : Stages of the medical abortion process (initial theory of change)

Searching for evidence and document selection

Our evidence search strategy started with a comprehensive literature search designed to answer a broader question for the SACHA project – this was a scoping review that sought to address the question: 'Interventions of models of abortion care/provision: addressing current therapeutic, technological and regulatory trends which would be relevant to or feasible in the UK in the next 5 years.' The databases and search terms for this search are listed in appendix 1 and the inclusion and exclusion criteria are listed in figure 1. The search dates were 01/01/2000 - 09/12/2022.

Figure 1: Inclusion/exclusion criteria for scoping review.

	Included	Excluded
Interventions		

[1] Page MJ, McKenzie JE, Bossuyt PM, Boutron I, Hoffmann TC, Mulrow CD, Shamseer L, Tetzlaff JM, Akl EA, Brennan SE, Chou R, Glanville J, Grimshaw JM, Hróbjartsson A, Lalu MM, Li T, Loder EW, Mayo-Wilson E, McDonald S, McGuinness LA, Stewart LA, Thomas J, Tricco AC, Welch VA, Whiting P, Moher D. The PRISMA 2020 statement: An updated guideline for reporting systematic reviews.

For peer review only - http://bmjopen.bmj.com/site/about/guidelines.xhtml

	Interventions of models of abortion care/provision: addressing current therapeutic, technological and regulatory trends which would be relevant to, or feasible in the UK in the next 5 years	Interventions aiming to legalise abortion, mitigate the effects of illegal abortion or address the financial aspects of abortion access. Interventions relevant to abortion care in unregulated/poorly regulated contexts. Pharmacological studies.
Populations	People seeking/having had an abortion, those accompanying someone through an abortion process, health care workers reporting experience of/attitudes towards abortion provision.	

Search results were imported into an electronic reference manager Covidence (https://www.covidence.org/) and screened in two stages. The first screen was based on title and abstract with a sample double-screened to check for consistency and discussion of studies of uncertain eligibility to reach consensus. The remainder of papers at the abstract and title stage and all those at the full text stage were single screened. The full text of all included documents were uploaded into a second electronic reference management system (Endnote).

We were confident that this search was comprehensive and contained all relevant information that was needed to answer our realist review question. We subsequently re-screened this search for the purposes of the realist review as follows:

1. A subject expert (WN) screened all full texts to identify documents that were specifically related to medical abortion at home and then grouped these into the stages outlined in our intitial programme theory.
2. PB read these papers to identify whether they were relevant to developing our initial programme theory.

Papers were considered useful for theory development and testing where they offered empirical evidence that could refine, refute or confirm developing emerging realist programme theory and inform the development of context-mechanism-outcome (CMO) configurations within it[28]. This meant including all papers that were relevant to people's experience of each stage of the medical abortion at home journey and the support provided that might modify this experience. We used a broad definition of 'support' to include any intervention by the individual having the abortion, the health care system or friends and family intended to improve or maintain health or wellbeing before, during and after the abortion process.

Realist review processes are iterative so as our CMO configurations developed we revisited the list of included papers for the scoping review to look for additional relevant materials. We also completed additional searches as required including handsearching reference lists and completing new searches, to seek out more relevant data.

For peer review only - http://bmjopen.bmj.com/site/about/guidelines.xhtml

Data extraction

The final selected papers regarding each stage of abortion care were read and re-read by PB and, for the post abortion contraception, by CF. Findings were summarised in spreadsheets which contained information on key relevant findings from each paper, grouped according to the programme theory. Based on their interpretations of these findings PB and CF developed CMO configurations (CMOCs) for each stage of the medical abortion process. No uniform data set was extracted from each paper, rather the data (verbatim sections of text) of each paper that were relevant to each emerging CMOCs were grouped together and iteratively used during the analysis process (see below) to develop CMO configurations.

Data analysis and synthesis

A realist logic of analysis uses data to produce causal explanations for outcomes that occur within a programme theory in the form of CMOCs. A CMOC is a proposition that explains what element of an initiative works, for whom and in what circumstances and is the primary way of reporting findings within a realist review. Within a CMOC, the causal claim being made is (in its simplest form), when a particular context is present, it 'triggers' or 'activates' a particular mechanism, which causes a particular outcome. Mechanisms are hidden causal processes that are context-sensitive and are usually inferred based on interpretations of the data. Data to inform our interpretation of the relationships between contexts, mechanisms and outcomes were sought within and across documents (e.g. mechanisms inferred from one document helped explain the way contexts influenced outcomes in a different document). Synthesising data from different documents is often necessary to compile CMOCs, since not all parts of the configurations will be articulated in every document. During our analysis, we used interpretive cross-case comparison to understand and explain how and why reported outcomes have occurred.

Refining programme theory

We iteratively refined our initial programme theory as the review progressed based on our interpretations of the data from the included papers. For each stage of the abortion at home process theory we sought to unpack what support is needed. Our final programme theory contains CMOCs that explain the outcomes achieved by the support provided, why it happens and in which contexts.

For peer review only - http://bmjopen.bmj.com/site/about/guidelines.xhtml

Results

Our searches identified 27,982 potentially relevant abstracts for both reviews. After duplicates and papers with incorrect dates or excluded literature types were removed 12,401 titles and abstracts were screened with 944 selected for full text assessment. At this stage, 590 studies were excluded which did not meet the inclusion criteria or were duplicates. The remaining full texts were screened for potential relevance to the realist review question and 244 studies were categorised by a content expert (WN) against the inclusion criteria and the initial programme theory. Additional detailed screening of these 244 studies indicated that 35 contained data that were relevant to support theory building and testing and hence were included. A further 12 papers were identified as relevant during the analysis through hand searching and expert consultation. At the end of the analysis, updating the searches identified 43 new abstracts of which 3 were included giving a total of 50 included papers (Figure 2). A table summarising the included papers is given in appendix 2.

Figure 2: PRISMA diagram

For peer review only

For peer review only - http://bmjopen.bmj.com/site/about/guidelines.xhtml

Our results are structured by our programme theory. For each stage, we present a narrative explanation of our findings followed by a table of the CMOCs that underpin the explanation. We found that the evidence on some of the stages in our programme theory were clustered and therefore the following stages are presented together:

- 'Understanding abortion options' and 'Choosing a method/setting'
- 'Accessing an abortion' and 'Pre-abortion counselling assessing gestational age'
- 'Taking mifepristone' and 'Taking misoprostol'.

Understanding abortion options and choosing a method

Choice of abortion methods and settings was influenced by service factors (number of appointments, timing and wait for appointments), personal responsibilities (caring/work commitments), geography (travel time/distance), relationships and need for privacy/secrecy and the extent to which people wished to be aware of/involved in/in control of the process [12, 29-35](Table 1). Those choosing medical abortion at home made this decision on the basis of their social responsibilities (such as the need to provide childcare) or when a home abortion enabled them to maintain their confidentiality, rather than their personal preferences about the procedure itself [12,21,29,35,36,37]. Conversely, some people chose hospital care because it limits knowledge of the procedure among household members (for example, an abusive or unsupportive partner) and requires less responsibility for the process[32,34]. Although we were not specifically seeking literature on reasons for surgical abortion, people cited a wish to be asleep/unaware of the procedure or to avoid pain/bleeding/sight of the products of conception ortaking the mifepristone[36,38] or because it could be completed quickly[39].

People valued frank descriptions from health professionals about the range of experience of medical abortion[40,31,39-43]. A participant in one study commented that 'It's good to do it at home but I think a good level of honesty from the nurse is helpful because it's on the cusp of being copeable with'[38]. Fear about the abortion procedure was the most frequently cited barrier to accessing an abortion in one study, with information as the most highly ranked facilitator to care[41]. High levels of satisfaction were associated with information on abortion delivered through short films in clinical contexts [43-45]. It is notable that in this study the film was rated as more informative than a leaflet even though the content was the same. Developing high quality information resources is challenging because of the need to offer both clear clinical instructions and a wide range of experience that enables people to prepare themselves, their supporters and their surroundings. One study evaluating an animated account of one person's experience of medical abortion at home showed that this approach was well received but participants were concerned that their own experience was different from that shown[43].

Table 1: CMOCs for 'understanding options' and 'choosing a method'

	CMOC
1	When people who want an abortion are offered a choice of procedures (c) they are more likely to be satisfied with their experience (i.e. predict choice of this method again) (o) because they feel their decision is their own (i.e. empowered in their choice) (m).[24,29, 36,37,38,40, 44, 45]
2	Where the information provided about each procedure option includes clinical safety and effectiveness and experiential information (c) people are in a better position to make an informed choice (o) because they are more likely to have the information needed to do so (m)[41,46,]

For peer review only - http://bmjopen.bmj.com/site/about/guidelines.xhtml

3	Where a range of personal experience is included that includes realistic accounts of pain/bleeding (c) then people are more likely to be satisfied with their choice (o) because they know what to expect and feel in control of the process (m)[31,39-43]
4	When people have social responsibilities in their lives (c), they will choose the method for abortion that fits best with these responsibilities (o) because of convenience (m)[21,29, 31,48]
5	When people would prefer not to take an active role in the abortion process (c), they may choose a surgical abortion (o) because it enables them to distance themselves from the abortion process (m)[23,31,36,47,48]
6	When people have doubts about their abilities to carry out a medical abortion properly (c), they are more likely to request they are closely supervised (e.g. complete the abortion within a health care context) (o), because of the emotional and practical support provided (m)[36-38,]
7	When people have had what they consider to be an unpleasant experience of medical abortion (c), they will be unlikely to choose medical abortion again (o), because they want to avoid the experience (m)[24,38,39,37,42,]

Accessing a service/pre-abortion consultation/assessing gestational age

People value telephone/telemedicine self-referral options[10,12,21], because of the privacy, autonomy and convenience they potentially afford and because they remove risk of judgmental attitudes from referring professionals (Table 2). People rated distance to clinic and waiting times as more important than whether counselling was via telemedicine or face to face[30] and reasons for preferring a telemedicine consultation included the ability to engage from a more comfortable space (at home) and feeling more able to ask questions[49]. However, some people need to take action to speak privately, for example, completing the consultation in their car[49,50].

The pre-abortion consultation is variable and may include an initial in-person consultation with/without an ultrasound scan and/or an examination or a fully remote service including self-reporting of gestational age[22]. Medical abortion without a prior ultrasound scan is safe[12] and in one qualitative study showed that some people preferred not to have an ultrasound scan because they associated it with having positive experiences of desired pregnancy[49] and in another study ultrasound was rated as unimportant for 53% of participants.

Table 2: CMOCs for 'Accessing a service/pre-abortion consultation

	CMOC
1	Where people can self-refer for abortion services via telephone or telemedicine services (c) they are more likely to be satisfied with this process (o) because they can avoid feeling judged by referring professionals (m).[22,50-52]
2	When people who want privacy, convenience and rapid access to abortion are offered the initial consultation by telephone or telemedicine (c), they are more likely to be satisfied (o), because their remote consultations are often available more quickly and are less likely to disrupt existing responsibilities (m)[21,29,30,48,49]

Taking the abortion medications

Most of the literature focuses on the experience of taking misoprostol because this generates the most physical side effects of medical abortion; however, some studies suggest that people may find taking the mifepristone the most emotional part of the treatment regimen and that this should be acknowledged in the information and support provided[40] (table 3). Self-administering misoprostol enabled people to control the timing of their abortion and prepare by making a comfortable space or obtaining things that they might need such as analgesia or hot water bottles, fit the abortion

For peer review only - http://bmjopen.bmj.com/site/about/guidelines.xhtml

around other responsibilities or keep it private[24]. When given a choice of how to take the misoprostol, 70% in one study chose to self-administer it sublingually, rather than vaginally because they felt more confident that they had inserted it correctly using this approach[21]. Side effects from the misoprostol were common, with 77% of people experiencing one of the common symptoms (nausea, vomiting, diarrhoea or headaches)[21].

Table 3: CMOCs for 'Taking the abortion medication'

	**CMOC**
1	When people who want an abortion have reservations about their active participation in the process (c) they may prefer not to self-administer the medications (o) because they feel personally responsible for ending the pregnancy (m)[40]
2	When people are given the option of self-administering misoprostol (c) they are better able to prepare for the process (o) because they can choose the time and place of the medical abortion (m)[24]
3	When people lack confidence in their ability to self-administer misoprostol (c) they prefer that this be done by a health professional (o) because they can then be confident that the procedure is done correctly (m)[21,24,38]

Managing expulsion

Experience of managing the expulsion of the products of conception is highly variable (Table 4). People interviewed after medical abortion at home point to the importance of understanding 'what is normal' as an important factor to help manage anxiety and to guide seeking clinical advice. Remote clinical support is important and highly valued and is used by 10-50% of people who choose medical abortion at home [24,37,53,21].

While most people find the process acceptable, the evidence shows that some experience pain and bleeding that is unexpected or distressing and cite this as the reason for future preference for an alternative method [24,37,38]. In one study 94% of respondents had experienced pain with a median reported pain score of 6.7 (on a scale of 1-10) though 57% reported their pain as better or the same as they were expecting[54]. 93% took analgesia This is consistent with mean maximum pain scale of 6-8 reported elsewhere[55]. In one study half of people experienced more bleeding than they anticipated[37] and in another 77% of participants reported that it was more than their usual menstrual period[54].

The pain relief offered to people completing medical abortion at home is not standard across services. When people completing a medical abortion at home were provided with a combination of paracetamol, ibuprofen and rectal diclofenac, almost all used all three medications, one quarter accessed additional analgesia and 65% used additional non-pharmacological measures such as heat and rest to reduce pain[37]. In recent UK studies, 93% used pain relief (41-92% paracetamol, 39-61% ibuprofen 64% dihydrocodeine/codein)[25,54].

Most people who experienced significant pain and bleeding felt more comfortable coping with this at home, "…it is such a physical and emotional process so, y'know, at home's better. You basically need to make friends with your toilet for 8 hours, so at home's better. I was such a mess. I would be embarrassed for people to see me like that."[38]. However, a minority would have preferred to have in-person health professional care because of the practical and emotional support provided[37,24,23].

When people having medical abortion at home have had a previous pregnancy (ending in live birth, abortion or miscarriage), they are less likely to report pain that is distressing or to request professional support in comparison with those who have not had a previous pregnancy. However, they are no less likely to report heavy bleeding[37]. One reason to choose medical abortion at home is that people wish to be aware of the process of the abortion. Some find it helpful to know that the pregnancy has been passed if they see the products of conception, while others find it distressing[38].

For peer review only - http://bmjopen.bmj.com/site/about/guidelines.xhtml

Some people having an abortion valued the support of partners, family and friends for company or practical support while others valued the opportunity to complete the abortion alone[24]. Partners (all men in cited study) interviewed about their experience of supporting people having a medical abortion expressed feelings of sadness, worry and concern, and sometimes ambivalence, about the decision to have an abortion as well as valuing the opportunity to support their partners through the process [56].

Table 4: CMOCs for 'Managing expulsion'

	CMOC
1	Where people experience pain or bleeding that is outside the limits of what they were told to expect (c) they are more likely to request clinical support (o) because they are concerned that their health may be at risk (m)[23,24,37]
2	Where people having medical abortion at home have also had a previous pregnancy (live birth/abortion/miscarriage) (c), they are less likely to report pain that is distressing and to request professional support (o) because the experience is more familiar (m)[37,50]
3	Where people receive adequate pain relief during medical abortion (c) they are more likely to be satisfied with the whole experience of medical abortion at home (o) as they were more comfortable (m)[24,37,38]
4	Where people want to have privacy when having an abortion (c), they may choose to manage the abortion process at home (o) because they feel less exposed (m) [35,38]
5	Where people have family/friends/partners whom they know will be supportive when they have an abortion (c) then they value their presence during a medical abortion at home (o) because of the practical and emotional help that they provide (m).[24,56]

Assessing the success of abortion

There is good evidence to suggest that people can self-assess the success of abortion[57,58,59, 60,61,62] (Table 5). There was little evidence in the literature of people's thoughts and feelings about this process.

Table 5: CMOC for 'Assessing the success of abortion'

	CMOC
1	When people are made aware of the symptoms and signs that indicate that the abortion has been successful (c), they are able to make accurate assessments (o) because they know what to look for (m) [57-62].

Choosing contraception

For some people the time of abortion provision is a convenient, appropriate and valued opportunity to discuss contraception and national/international guidelines recommend this[58] (Table 6). For those people opting for implants, having them inserted when medical abortion medication is provided, or for those choosing an intrauetine devices, an appointment booked shortly after medical abortion, means they are more likely to receive their contraceptive of choice [38,63,64]. However, medical abortion via telemedicine limits the opportunity to do this. For other people, a contraceptive discussion at the time of the medical abortion consultation is unwelcome[65] and may be associated with a sense of being judged and pressurised about contraception[38] . Some people also prefer to focus on managing their abortion before considering contraception[38,65-67]. In this situation the offer of a delayed telephone or face to face contraception discussion can be welcome including for those who have had difficulty finding a contraceptive method that suits them[38,68,69,70,71].

Table 6: CMOCs for 'Choosing contraception'

	**CMOC**

For peer review only - http://bmjopen.bmj.com/site/about/guidelines.xhtml

1	At the time of abortion provision (c) some people are happy to discuss or be provided with contraception (o) because of the salience of this issue for them and the convenience (m)[38]
2	When people opting for long-acting reversible contraceptives (LARC) have an appointment booked for them when receiving abortion medication at a time and place of their choosing after the abortion, (c) they are more likely to receive their contraception of their choice (o) because of salience (m) and less work for users in making the appointment (m)[63,64]
3	When people who are focused on the abortion procedure are offered contraceptive advice at a later date (c), they are more likely to welcome a discussion about contraception (o), because they have the capacity to consider this (m)[38,68,69,70,71]
4	When people who have had a previous abortion are offered telephone contraceptive counselling following abortion (c) they are more likely to take this up (o) because they may want help with complex contraceptive needs (m) [68,70,71]
5	When contraception is raised at the time of abortion (c), people can feel judged (o) if health care providers imply that it is important to prevent future unintended pregnancies (m) [38]
C6	When blended (digital linked to face to face) post abortion support and contraception services are designed with user input (c) they are likely to be acceptable and accessible to users (o) because they address their needs (m).[72]

For peer review only - http://bmjopen.bmj.com/site/about/guidelines.xhtml
For peer review only

Discussion

Choice of abortion procedure remains essential

Increased expectations of self-care, access to digital health services and the COVID 19 pandemic may have reduced choice of abortion method. High levels of satisfaction are reported after medical abortion but variation in experience means that choice of method remains important[21,23,31,36]. Though medical abortion at home is a lower cost procedure than medical abortion in hospital which, in turn, is a lower cost procedure than surgical abortion[73], personal preference, past experience or context may make some abortion processes, for some people, difficult, distressing or unsafe [74,75]. People who may have lower levels of satisfaction with medical abortion at home include those who: have not had a previous pregnancy, do not have access to a comfortable and private space at home, do not have support from family/partners/friends, have inflexible personal or employment responsibilities, are homeless or wish to disengage from the process.

Realistic information on the range of experience of abortion at home is important so that the abortion does not generate surprise or anxiety and so that people can adequately prepare for it at home.

Informed decisions require information on the potential advantages and disadvantages of the available treatment options and abortion providers should offer information on the effectiveness, safety, health risks and benefits and the expected range of experiences of all abortion options. Although the effectiveness of abortion procedures is important to inform choice as up to 5% may require a surgical evacuation to complete the abortion[76-78], effectiveness was not mentioned in user accounts of reasons for choosing medical abortion at home. Our review shows that it is important to communicate: a range of experiences, the importance of context (for example, where there is lack of privacy) or personal experience (for example, those with previous pregnancies report less pain). There is limited recent research on the best strategies to communicate this information and further work is required to collate and test good practice.

Acknowledging the implications of moving a process from a health care setting to home

Guidelines on support for medical abortion at home may not sufficiently acknowledge the work that people do to prepare their space, manage their privacy, fulfil their work and caring obligations around the process, make decisions on pain relief, clean household areas, decide when and how to take misoprostol, assess their experience against what is normal and decide when to ask for professional help. There is a need to understand the new types of information/advice required to support people who do this work. The response may involve new technologies (e.g. medical abortion support at home apps), new or developing roles (e.g. medical abortion doulas) or new forms of peer support (e.g. discussion forums to help with preparation and share experience). Further research is required to understand and define health service responsibilities when procedures move from clinically facilitated to self-managed in general and for medical abortion at home in particular.

Understanding optimal pain relief

There is no consensus within the literature on analgesia for those who experience pain during medical abortion although a Cochrane Review is currently in progress on this topic[79]. There is currently significant variation in practice described in terms of the analgesics offered, the schedule and route of administration and whether these are provided by health services.

More choice within the procedure

Enabling people to tailor their abortion experience could be an empowering strategy within a process that is often experienced as disempowering[32,80]. Options include when and how to take medications, pharmaceutical and other strategies for pain relief, the amount and form of support available and when contraception is discussed.

For peer review only - http://bmjopen.bmj.com/site/about/guidelines.xhtml

Contraception
Peri-abortion contraception counselling should be delivered without pressure to take up contraception as some people find this stigmatising. A choice of when and where to discuss contraception is important. The option of delayed counselling/provision is helpful for some and may be the only option for those who are having intrauterine devices or implants and choose telemedicine abortion.

Strengths and limitations
We were systematic and transparent in our approach to the realist review which was conducted in accordance with the RAMSES standards (27). Our authorship team represents a variety of clinical and academic backgrounds, ensuring divergence in our analysis and we benefited from expert feedback from the multi-disciplinary SACHA team throughout the review. Limitations include our analysis on publicly accessible literature, located through recognised research databases and Google. There were clear gaps in the evidence that we found and we have highlighted these in our conclusions.

Further research needed
Based on our review of the literature we recommend that further research is needed on:

- Optimal strategies for providing information and supporting choice of procedure/setting.
- Understanding how medical abortion at home involves or impacts family and friends (beyond partners).
- Optimal medications for pain relief including the dosing, route of administration and length of time that pain relief is required.
- How to offer choice within the abortion procedure in a manner that is empowering and not overwhelming
- Optimal means for providing contraception advice /support after the abortion procedure.

Recommendations
Our recommendations are summarized in our revised programme theory in figure 3 below.

- People should be given a choice of type and place of abortion
- Discussion of choice of method should include service factors, personal responsibilities, geographical context and preferred level of awareness of/engagement with the procedure
- Information to support choice of procedure should include the range of experience of medical abortion and realistic accounts of pain and bleeding.
- Health system support for the abortion process should acknowledge the new types of work that people take on when they manage this process at home, for example preparing a space for the abortion.
- There should be opportunities to personalize experience including when and where to take medications, who is present, where to pass products of conception.
- Support for taking mifepristone should acknowledge the emotional significance of this for some people.
- Adequate pain relief is an essential part of satisfaction with medical abortion, particularly for those who are pregnant for the first time. Pain relief should be offered as well as information on non-pharmacological strategies to manage pain.
- People should be offered a choice of where and when to discuss contraception including opportunities to book LARC appointments if this is their preference.

Conclusions
Acknowledging the work done by patients when moving an intervention from clinic to home is important. This includes preparing a space, managing privacy, managing work/caring obligations,

For peer review only - http://bmjopen.bmj.com/site/about/guidelines.xhtml

deciding when/how to take medications, understanding what is normal, assessing experience and deciding when and how to ask for help. Strategies that reduce surprise or anxiety and enable preparation and a sense of control support the transition of this complex intervention outside healthcare environments. It is important that options for those who are unable to take on these additional responsibilities or who prefer not to do so are maintained including clinical facilities and appropriately trained clinicians who can provide surgical abortion and medical abortion within health care facilities.

Figure 3: Revised programme theory with key learning from this review

References

1. Abortion statistics, England and Wales, 2020. (https://www.gov.uk/government/statistics/abortion-statistics-for-england-and-wales-2020/abortion-statistics-england-and-wales-2020
2. Public Health Scotland termination of pregnancy statistics https://publichealthscotland.scot/publications/termination-of-pregnancy-statistics/termination-of-pregnancy-statistics-year-ending-december-2020/).
3. Altshuler AL, Whaley NS. The patient perspective: perceptions of the quality of the abortion experience. Curr Opin Obstet Gynecol. 2018 Dec;30(6):407-413. doi: 10.1097/GCO.0000000000000492. PMID: 30299320.
4. Biggs MA, Ralph L, Raifman S, Foster DG, Grossman D. Support for and interest in alternative models of medication abortion provision among a national probability sample of U.S. women. Contraception. 2019 Feb;99(2):118-124. doi: 10.1016/j.contraception.2018.10.007. Epub 2018 Nov 15. PMID: 30448203.
5. Darney BG, Powell B, Andersen K, et al. Quality of care and abortion: beyond safety [published online ahead of print, 2018 May 7]. *BMJ Sex Reprod Health*. 2018;44(3):159-160. doi:10.1136/bmjsrh-2018-200060
6. Grossman, D., Grindlay, K., Buchacker, T., Lane, K., & Blanchard, K. (2011). Effectiveness and acceptability of medical abortion provided through telemedicine. *Obstet Gynecol*, *118*(2 Pt 1), 296-303. https://doi.org/https://dx.doi.org/10.1097/AOG.0b013e318224d110
7. Fok WK, Mark A. Abortion through telemedicine. Curr Opin Obstet Gynecol. 2018 Dec;30(6):394-399. doi: 10.1097/GCO.0000000000000498. PMID: 30399015.
8. Aiken, A., Gomperts, R., & Trussell, J. (2017). Experiences and characteristics of women seeking and completing at-home medical termination of pregnancy through online telemedicine in Ireland and Northern Ireland: a population-based analysis. *BJOG*, *124*(8), 1208-1215. https://doi.org/https://dx.doi.org/10.1111/1471-0528.14401
9. Aiken, A. R. A., Digol, I., Trussell, J., & Gomperts, R. (2017). Self reported outcomes and adverse events after medical abortion through online telemedicine: population based study in the Republic of Ireland and Northern Ireland. *BMJ*, *357*, j2011. https://doi.org/10.1136/bmj.j2011
10. Aiken, A. R. A., Broussard, K., Johnson, D. M., & Padron, E. (2018). Motivations and Experiences of People Seeking Medication Abortion Online in the United States. *Perspect Sex Reprod Health*, *50*(4), 157-163.

For peer review only - http://bmjopen.bmj.com/site/about/guidelines.xhtml

11. Aiken, A. R. A., Starling, J. E., Gomperts, R., Scott, J. G., & Aiken, C. E. (2021a). Demand for self-managed online telemedicine abortion in eight European countries during the COVID-19 pandemic: a regression discontinuity analysis. *BMJ Sex Reprod Health*, bmjsrh-2020-200880. https://doi.org/10.1136/bmjsrh-2020-200880
12. Aiken, A., Lohr, P. A., Lord, J., Ghosh, N., & Starling, J. (2021b). Effectiveness, safety and acceptability of no-test medical abortion (termination of pregnancy) provided via telemedicine: a national cohort study. *BJOG*, *128*(9), 1464-1474. https://doi.org/10.1111/1471-0528.16668
13. Chong, E., Shochet, T., Raymond, E., Platais, I., Anger, H. A., Raidoo, S., Soon, R., Grant, M. S., Haskell, S., Tocce, K., Baldwin, M. K., Boraas, C. M., Bednarek, P. H., Banks, J., Coplon, L., Thompson, F., Priegue, E., & Winikoff, B. (2021). Expansion of a direct-to-patient telemedicine abortion service in the United States and experience during the COVID-19 pandemic. *Contraception*, *104*(1), 43-48. https://doi.org/10.1016/j.contraception.2021.03.019
14. Gambir, K., Garnsey, C., Necastro, K. A., & Ngo, T. D. (2020). Effectiveness, safety and acceptability of medical abortion at home versus in the clinic: a systematic review and meta-analysis in response to COVID-19. *BMJ Glob Health*, *5*(12). https://doi.org/10.1136/bmjgh-2020-003934
15. Grossman, D., Baba, C. F., Kaller, S., Biggs, M. A., Raifman, S., Gurazada, T., Rafie, S., Averbach, S., Meckstroth, K. R., Micks, E. A., Berry, E., Raine-Bennett, T. R., & Creinin, M. D. (2021). Medication Abortion With Pharmacist Dispensing of Mifepristone. *Obstetrics & Gynecology*, *Latest Articles*(online first),
16. Raymond, E., Chong, E., Winikoff, B., Platais, I., Mary, M., Lotarevich, T., Castillo, P. W., Kaneshiro, B., Tschann, M., Fontanilla, T., Baldwin, M., Schnyer, A., Coplon, L., Mathieu, N., Bednarek, P., Keady, M., & Priegue, E. (2019). TelAbortion: evaluation of a direct to patient telemedicine abortion service in the United States. *Contraception*, *100*(3), 173-177. https://doi.org/https://dx.doi.org/10.1016/j.contraception.2019.05.013
17. Schummers L, Darling EK, Dunn S, McGrail K, Gayowsky A, Law MR, et al. Abortion Safety and Use with Normally Prescribed Mifepristone in Canada. New England Journal of Medicine. 2022;386(1):57-67.
18. Upadhyay, U. D., Desai, S., Zlidar, V., Weitz, T. A., Grossman, D., Anderson, P., & Taylor, D. (2015). Incidence of emergency department visits and complications after abortion. *Obstet Gynecol*, *125*(1), 175-183. https://doi.org/10.1097/AOG.0000000000000603
19. Upadhyay, U. D., Schroeder, R., & Roberts, S. C. M. (2020). Adoption of no-test and telehealth medication abortion care among independent abortion providers in response to COVID-19. *Contracept X*, *2*, 100049. https://doi.org/10.1016/j.conx.2020.100049
20. Upadhyay, U. D., Koenig, L. R., & Meckstroth, K. R. (2021). Safety and Efficacy of Telehealth Medication Abortions in the US During the COVID-19 Pandemic. *JAMA Network Open*, *4*(8), e2122320. https://doi.org/10.1001/jamanetworkopen.2021.22320
21. Reynolds-Wright JJ, Johnstone A, McCabe K, Evans E, Cameron S. Telemedicine medical abortion at home under 12 weeks' gestation: a prospective observational cohort study during the COVID-19 pandemic. BMJ Sex Reprod Health. 2021

For peer review only - http://bmjopen.bmj.com/site/about/guidelines.xhtml

Oct;47(4):246-251. doi: 10.1136/bmjsrh-2020-200976. Epub 2021 Feb 4. PMID: 33542062; PMCID: PMC7868129.

22. Gill R, Norman WV. Telemedicine and medical abortion: dispelling safety myths, with facts. Mhealth. 2018 Feb 1;4:3. doi: 10.21037/mhealth.2018.01.01. PMID: 29552565; PMCID: PMC5847856.
23. Hoggart L, Berer M. Making the case for supported self-managed medical abortion as an option for the future. *BMJ Sexual & Reproductive Health* Published Online First: 23 June 2021. doi: 10.1136/bmjsrh-2021-201181
24. Harden J, Ancian J, Cameron S*, et al*. Women's experiences of self-administration of misoprostol at home as part of early medical abortion: a qualitative evaluation. *BMJ Sexual & Reproductive Health* 2021;**47:**144-149.
25. Meurice ME, Whitehouse KC, Blaylock R, Chang JJ, Lohr PA. Client satisfaction and experience of telemedicine and home use of mifepristone and misoprostol for abortion up to 10 weeks' gestation at British Pregnancy Advisory Service: A cross-sectional evaluation. Contraception. 2021 Jul;104(1):61-66. doi: 10.1016/j.contraception.2021.04.027. Epub 2021 May 8. PMID: 33974918.
26. Pawson R. Evidence Based Policy; a realist perspective. Sage, London, 2006
27. Wong, G., Westhorp, G., Manzano, A. *et al.* RAMESES II reporting standards for realist evaluations. *BMC Med* **14,** 96 (2016). https://doi.org/10.1186/s12916-016-0643-1
28. Duddy C, Wong G, Gadsby EW, Krska J, Hibberd V. NHS Health Check programme: a protocol for a realist review. BMJ Open. 2021 Apr 14;11(4):e048937. doi: 10.1136/bmjopen-2021-048937. PMID: 33853809; PMCID: PMC8054100.
29. Fix L, Seymour JW, Sandhu MV*, et al* At-home telemedicine for medical abortion in Australia: a qualitative study of patient experiences and recommendations. *BMJ Sexual & Reproductive Health* 2020;**46:**172-176.
30. Endler, M, Lavelanet, A, Cleeve, A, Ganatra, B, Gomperts, R, Gemzell-Danielsson, K. Telemedicine for medical abortion: a systematic review. *BJOG* 2019; 126: 1094– 1102.
31. Heath J, Mitchell N, Fletcher J. A comparison of termination of pregnancy procedures: Patient choice, emotional impact and satisfaction with care. Sex Reprod Healthc. 2019 Mar;19:42-49. doi: 10.1016/j.srhc.2018.12.002. Epub 2018 Dec 3. PMID: 30928134.
32. Killinger K, Günther S, Gomperts R, Atay H, Endler M. Why women choose abortion through telemedicine outside the formal health sector in Germany: a mixed-methods study. BMJ Sex Reprod Health. 2022 Jan;48(e1):e6-e12. doi: 10.1136/bmjsrh-2020-200789. Epub 2020 Nov 23. PMID: 33229399.
33. Wiebe E. and Sandhu K.Access to abortion: what women want from abortion services. Journal of obstetrics and gynaecology Canada, 2008; 30:327-331.
34. Hamoda H, Critchley HO, Paterson K, Guthrie K, Rodger M, Penney GC. The acceptability of home medical abortion to women in UK settings. BJOG. 2005 Jun;112(6):781-5. doi: 10.1111/j.1471-0528.2004.00538.x. PMID: 15924537.
35. Atay H, Perivier H, Gemzell-Danielsson K*, et al*. Why women choose at-home abortion via teleconsultation in France: drivers of telemedicine abortion during and beyond the COVID-19 pandemic. *BMJ Sexual & Reproductive Health* 2021;**47:**285-292.

For peer review only - http://bmjopen.bmj.com/site/about/guidelines.xhtml

36. Robson SC, Kelly T, Howel D, Deverill M, Hewison J, Lie ML, Stamp E, Armstrong N, May CR. Randomised preference trial of medical versus surgical termination of pregnancy less than 14 weeks' gestation (TOPS). Health Technol Assess. 2009 Nov;13(53):1-124, iii-iv. doi: 10.3310/hta13530. PMID: 19906334.
37. Hedqvist M, Brolin L, Tydén T, Larsson M. Women's experiences of having an early medical abortion at home. Sex Reprod Healthc. 2016 Oct;9:48-54. doi: 10.1016/j.srhc.2016.07.003. Epub 2016 Aug 2. PMID: 27634665.
38. Purcell, C, Cameron, S, Lawton, J, Glasier, A, Harden, J. Self-management of first trimester medical termination of pregnancy: a qualitative study of women's experiences. *BJOG* 2017; 124: 2001– 2008.
39. Levine K, Cameron ST. Women's preferences for method of abortion and management of miscarriage. J Fam Plann Reprod Health Care. 2009 Oct;35(4):233-5. doi: 10.1783/147118909789587394. PMID: 19849917.
40. Makenzius M, Tydén T, Darj E, Larsson M. Autonomy and dependence--experiences of home abortion, contraception and prevention. Scand J Caring Sci. 2013 Sep;27(3):569-79. doi: 10.1111/j.1471-6712.2012.01068.x. Epub 2012 Aug 22. PMID: 22913927.
41. Smith JL, Cameron S. Current barriers, facilitators and future improvements to advance quality of abortion care: views of women. BMJ Sex Reprod Health. 2019 Apr 26:bmjsrh-2018-200264. doi: 10.1136/bmjsrh-2018-200264. Epub ahead of print. PMID: 31028169.
42. Rafferty KA, Longbons T. *#AbortionChangesYou*: A Case Study to Understand the Communicative Tensions in Women's Medication Abortion Narratives. Health Commun. 2021 Nov;36(12):1485-1494. doi: 10.1080/10410236.2020.1770507. Epub 2020 Jun 1. PMID: 32482094.
43. Sherman S, Harden J, Cattanach D, Cameron ST. Providing experiential information on early medical abortion: a qualitative evaluation of an animated personal account, *Lara's Story*. J Fam Plann Reprod Health Care. 2017 Oct;43(4):269-273. doi: 10.1136/jfprhc-2016-101641. Epub 2017 Jul 8. PMID: 28689176.
44. Powell-Jackson R, Glasier A, Cameron ST. Benefits of using a digital video disk for providing information about abortion to women requesting termination of pregnancy. Contraception. 2010 Jun;81(6):537-41. doi: 10.1016/j.contraception.2010.01.015. Epub 2010 Feb 19. PMID: 20472123.
45. Reynolds-Wright JJ, Belleuvre F, Daberius A, Faucher P, Gemzell-Danielsson K, Cameron ST. Information on early medical abortion for women using an audiovisual animation vs face-to-face consultation: A consortium randomized and quasi-randomized trial. Acta Obstet Gynecol Scand. 2020 Dec;99(12):1611-1617. doi: 10.1111/aogs.13944. Epub 2020 Jul 12. PMID: 32573767.
46. Norman WV, Hestrin B, Dueck R. Access to Complex Abortion Care Service and Planning Improved through a Toll-Free Telephone Resource Line. Obstet Gynecol Int. 2014;2014:913241. doi: 10.1155/2014/913241. Epub 2014 Feb 13. PMID: 24693291; PMCID: PMC3945945.
47. Whitehouse, K.C., Blaylock, R., Makleff, S. *et al. It's a small bit of advice, but actually on the day, made such a difference…*: perceptions of quality in abortion care in England and Wales. *Reprod Health* **18,** 221 (2021). https://doi.org/10.1186/s12978-021-01270-0

For peer review only - http://bmjopen.bmj.com/site/about/guidelines.xhtml

48. Rowlands, Sam, and Jeffrey Wale. “A Constructivist Vision of the First-Trimester Abortion Experience.” *Health and human rights* vol. 22,1 (2020): 237-249.
49. Boydell, N, Reynolds-Wright, JJ, Cameron, ST, Harden, J. Women’s experiences of a telemedicine abortion service (up to 12 weeks) implemented during the coronavirus (COVID-19) pandemic: a qualitative evaluation. *BJOG* 2021;https://doi.org/10.1111/1471-0528.16813 128: 1752– 1761.
50. Porter Erlank C, Lord J, Church K. Acceptability of no-test medical abortion provided via telemedicine during Covid-19: analysis of patient-reported outcomes. *BMJ Sexual & Reproductive Health* 2021;**47:**261-268.
51. Low ST, Chen ZE, Cameron S. Women’s experiences of self-referral to an abortion service: qualitative study. *BMJ Sexual & Reproductive Health* 2021;**47:**37-42.
52. Ehrenreich, K., Marston, C. Spatial dimensions of telemedicine and abortion access: a qualitative study of women’s experiences. *Reprod Health* **16,** 94 (2019). https://doi.org/10.1186/s12978-019-0759-9
53. Finch RE, McGeechan K, Johnstone A*, et al.* Impact of self-administration of misoprostol for early medical abortion: a prospective observational cohort study. *BMJ Sexual & Reproductive Health* 2019;**45:**296-301.
54. Reynolds-Wright JJ, Johnstone A, McCabe K*, et al.* Adherence to treatment and prevalence of side effects when medical abortion is delivered via telemedicine: a prospective observational cohort study during COVID-19. *BMJ Sexual & Reproductive Health* Published Online First: 28 October 2021. doi: 10.1136/bmjsrh-2021-201263
55. Goldman AR, Porsch L, Hintermeister A, Dragoman M. Transcutaneous Electrical Nerve Stimulation to Reduce Pain With Medication Abortion: A Randomized Controlled Trial. Obstet Gynecol. 2021 Jan 1;137(1):100-107. doi: 10.1097/AOG.0000000000004208. PMID: 33278292.
56. Kero A, Lalos A, Wulff M. Home abortion - experiences of male involvement. Eur J Contracept Reprod Health Care. 2010 Aug;15(4):264-70. doi: 10.3109/13625187.2010.485257. PMID: 20809674.
57. Schmidt-Hansen M, Cameron S, Lohr PA, Hasler E. Follow-up strategies to confirm the success of medical abortion of pregnancies up to 10 weeks' gestation: a systematic review with meta-analyses. Am J Obstet Gynecol. 2020 Jun;222(6):551-563.e13. doi: 10.1016/j.ajog.2019.11.1244. Epub 2019 Nov 9. PMID: 31715147
58. Eshre Capri Workshop Group. (2017). Induced abortion. *Hum Reprod*, *32*(6), 1160-1169. https://doi.org/10.1093/humrep/dex071
59. Lokeland, M., Iversen, O. E., Engeland, A., Okland, I., & Bjorge, L. (2014). Medical abortion with mifepristone and home administration of misoprostol up to 63 days' gestation. *Acta Obstet Gynecol Scand*, *93*(7), 647-653. https://doi.org/10.1111/aogs.12398
60. Oppegaard, K. S., Qvigstad, E., Fiala, C., Heikinheimo, O., Benson, L., & Gemzell-Danielsson, K. (2015). Clinical follow-up compared with self-assessment of outcome after medical abortion: a multicentre, non-inferiority, randomised, controlled trial. *Lancet*, *385*(9969), 698-704. https://doi.org/10.1016/S0140-6736(14)61054-0
61. Raymond, E. G., Shochet, T., & Bracken, H. (2018). Low-sensitivity urine pregnancy testing to assess medical abortion outcome: A systematic review. *Contraception*, *98*(1), 30-35. https://doi.org/10.1016/j.contraception.2018.03.013
62. Raymond, E. G., Tan, Y.-L., Grant, M., Benavides, E., Reis, M., Sacks, D. N., Hannum, C., Frapp, S., & Weaver, M. A. (2018). Self-assessment of medical abortion outcome

For peer review only - http://bmjopen.bmj.com/site/about/guidelines.xhtml

using symptoms and home pregnancy testing. *Contraception*, *97*(4), 324-328. https://doi.org/https://dx.doi.org/10.1016/j.contraception.2017.12.004
63. Pohjoranta E, Mentula M, Suhonen SP*, et al.* Predicting poor compliance with follow-up and intrauterine contraception services after medical termination of pregnancy. *BMJ Sexual & Reproductive Health* 2018;**44:**278-285.
64. E. Pohjoranta, S. Suhonen, M. Gissler, P. Ikonen, M. Mentula and O. Heikinheimo. Early provision of intrauterine contraception as part of abortion care-5-year results of a randomised controlled trial. Human reproduction (Oxford, England) 2020 Vol. 35 Issue 4 Pages 796-804
65. Iyer A, Wallace R, Kindratt TB, Gimpel N. Preferences for contraceptive counseling and access among abortion patients at an independent clinic in Texas. Patient Educ Couns. 2021 Sep 9:S0738-3991(21)00619-4. doi: 10.1016/j.pec.2021.09.010. Epub ahead of print. PMID: 34538463.
66. Matulich M., Cansino C., Culwell K.R., and Creinin M. D. Understanding women's desires for contraceptive counseling at the time of first-trimester surgical abortion. Contraception 2014; 89 (1):36-41
67. Kavanagh M., Carlin E., Jones R. Patient's attitudes and experiences related to receiving contraception during abortion care. Contraception, 2011; 84 (6): 585-593
68. Lohr PA, Aiken ARA, Forsyth T*, et al.* Telephone or integrated contraception counselling before abortion: impact on method choice and receipt. *BMJ Sexual & Reproductive Health* 2018;**44:**114-121.
69. Smith, C., Ly, S., Uk, V. *et al.* Women's views and experiences of a mobile phone-based intervention to support post-abortion contraception in Cambodia. *Reprod Health* **14,** 72 (2017). https://doi.org/10.1186/s12978-017-0329-y
70. Smith, C., Ly, S., Uk, V. *et al.* Process evaluation of a mobile phone-based intervention to support post-abortion contraception in Cambodia. *Contracept Reprod Med* **2,** 16 (2017). https://doi.org/10.1186/s40834-017-0043-8
71. Loeber. O. Contraceptive counselling to prevent repeat unintended pregnancy: The abortion client's perspective. European Journal of Contraception and Reproductive Health Care 2016 Vol. 21 Issue SUPPL. 1 Pages 75
72. Gill R, Ogilvie G, Norman WV, Fitzsimmons B, Maher C, Renner R Feasibility and Acceptability of a Mobile Technology Intervention to Support Postabortion Care in British Columbia: Phase I. J Med Internet Res 2019;21(5):e13387
73. NHS reference costs 2019/20 V2 https://www.england.nhs.uk/national-cost-collection/#nccdata2. Accessed 15/02/2022
74. Wingo E, Ralph LJ, Kaller S, Biggs MA. Abortion method preference among people presenting for abortion care. Contraception. 2021 Apr;103(4):269-275. doi: 10.1016/j.contraception.2020.12.010. Epub 2020 Dec 26. PMID: 33373612.
75. Blaylock R, Makleff S, Whitehouse KC*, et al*. Client perspectives on choice of abortion method in England and Wales. *BMJ Sexual & Reproductive Health* Published Online First: 20 September 2021. doi: 10.1136/bmjsrh-2021-201242
76. Creinin, M. D., & Grossman, D. A. (2020). Medication Abortion Up to 70 Days of Gestation: ACOG Practice Bulletin, Number 225. *Obstetrics & Gynecology*, *136*(4), e31-e47. https://doi.org/10.1097/aog.0000000000004082
77. Royal College of Obstetricians and Gynaecologists. (2015). *Comprehensive Abortion Care* (Best Practice Paper No. 2, Issue. Royal College of Obstetricians &

For peer review only - http://bmjopen.bmj.com/site/about/guidelines.xhtml

Gynaecologists. https://www.rcog.org.uk/en/guidelines-research-services/guidelines/bpp2/
78. Royal College of Obstetricians and Gynaecologists. (2019). *Clinical Guidelines for Early Medical Abortion at Home – England*. Royal College of Obstetricians & Gynaecologists. https://www.rcog.org.uk/en/guidelines-research-services/guidelines/early-medical-abortion-home-england/
79. Reynolds-Wright JJ, Woldetsadik MA, Morroni C, Cameron S. Pain management for medical abortion before 14 weeks' gestation. Cochrane Database of Systematic Reviews 2020, Issue 1. Art. No.: CD013525. DOI: 10.1002/14651858.CD013525
80. Ireland, S., Belton, S., & Doran, F. (2020). 'I didn't feel judged': exploring women's access to telemedicine abortion in rural Australia. Journal of primary health care, 12(1), 49-56. https://doi.org/https://dx.doi.org/10.1071/HC19050

For peer review only

For peer review only - http://bmjopen.bmj.com/site/about/guidelines.xhtml

For peer review only

For peer review only - http://bmjopen.bmj.com/site/about/guidelines.xhtml

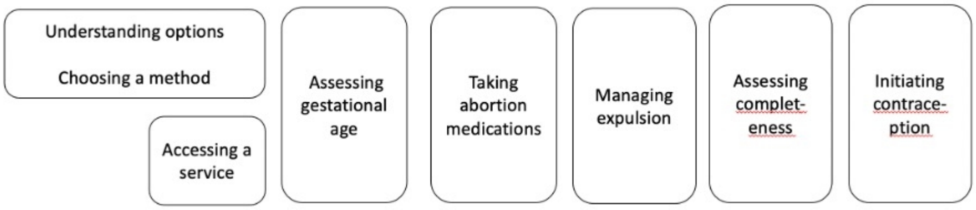

Figure 1. Stages of the medical abortion process (initial theory of change)

159x43mm (144 x 144 DPI)

Figure 2. PRISMA 2009 Flow Diagram

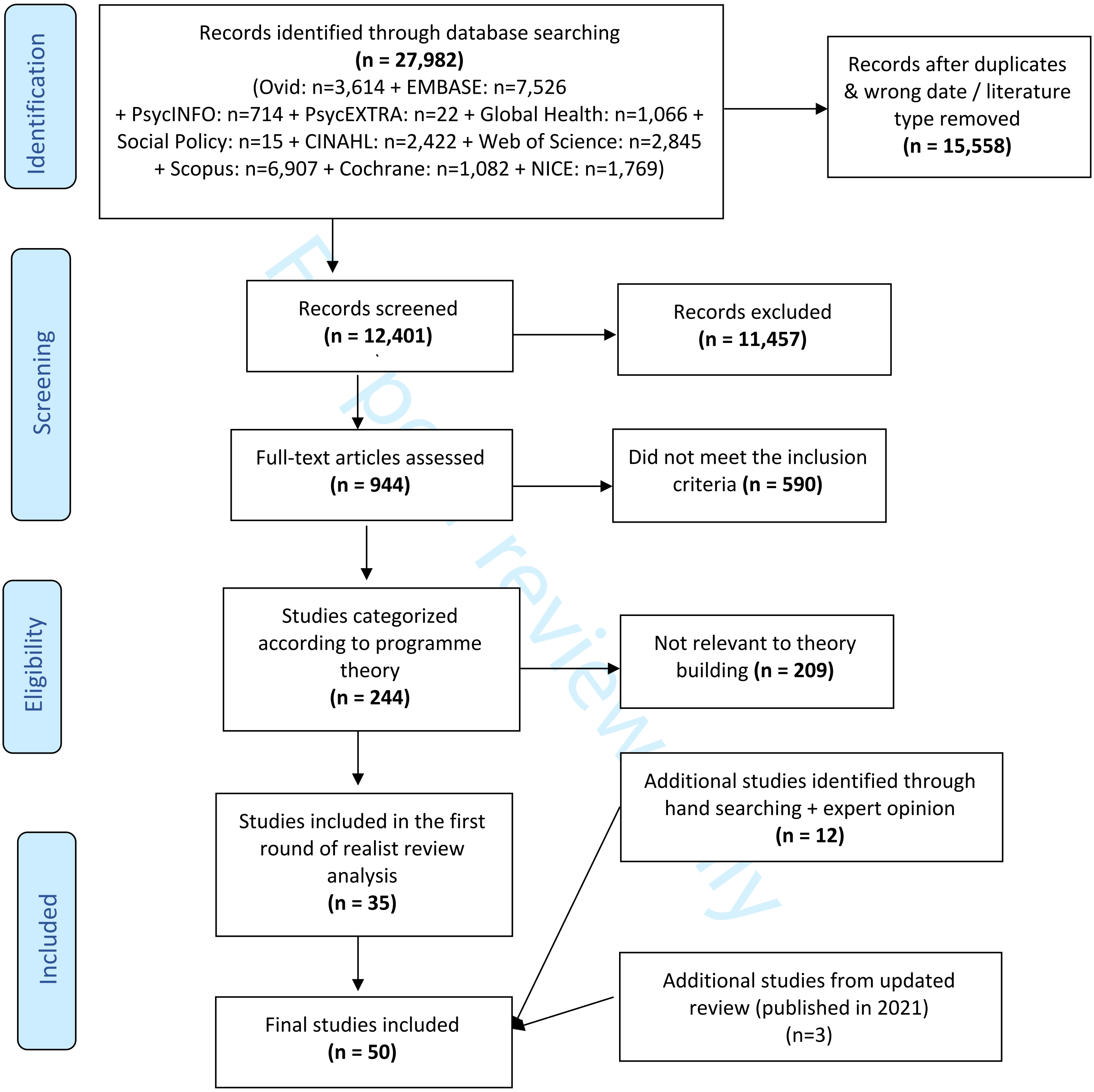

For peer review only - http://bmjopen.bmj.com/site/about/guidelines.xhtml

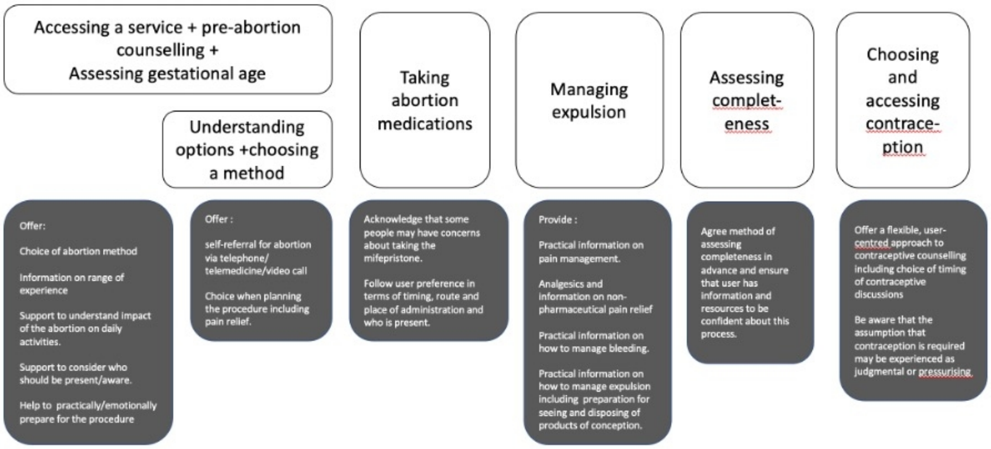

Figure 3. Revised programme theory with key learning from this review

159x74mm (144 x 144 DPI)

Appendix 1: Databases searched and search terms

Ovid MEDLINE(R) and Epub Ahead of Print, In-Process & Other Non-Indexed Citations, Daily and Versions(R) 1946 to December 8, 2021; Embase Classic+Embase 1947 to 2021 December 08; APA PsycInfo 1806 to November Week 5 2021; APA PsycExtra 1908 to November 08, 2021; Global Health 1910 to 2021 Week 49; Social Policy and Practice 202010; CINAHL Plus, Web fo Science Core collection; Scopus (Elsevier); Cochrane Library.

Seach terms for Ovid: ((exp Abortion, Induced/) OR (abortion) OR (abortions) OR (pregnancy adj4 terminat*)) AND ((self care/) OR (self-management/) OR (Self Administration/) OR (Telemedicine/) OR (exp communications media/) OR (Internet-Based Intervention/) OR (Text Messaging/) OR (Outpatients/) OR (nurses/) OR (nursing staff/) OR (Nurse Midwives/) OR (Midwifery/) OR (Pharmacists/) OR (exp General Practice/) OR (Primary Health Care/) OR (Patient-Centred Care/) OR (self-manag*) OR (self-administer*) OR (self-induc*) OR (self-assess*) OR (self-sourc*) OR ("at home") OR ("home use") OR (home adj4 dosage) OR (home adj4 administ*) OR (home adj4 abortion) OR (telemedic*) OR (digit*) OR (online) OR (web) OR (website) OR (internet) OR (mobile) OR (sms) OR (text*) OR (messag*) OR (app) OR ("social media") OR (outpatient) OR (task-shar*) OR (task-shift*) OR (mid-level) OR (midlevel) OR (nurs*) OR (midwi*) OR (pharmacist) OR (pharmacy) OR (GP) OR (GPs) OR (general adj3 practic*) OR (non-speciali*) OR (non-clinical) OR (licen*) OR (qualif*) OR (decrim*) OR (woman-centered) OR (woman-centred) OR (women-centered) OR (women-centred) OR (patient-centered) OR (patient-centred) OR (gestation* adj5 limit*)) AND (("Patient Acceptance of Health Care"/) OR (Health Services Accessibility/) OR (exp patient satisfaction/ or patient preference/) OR (access*) OR (safe*) OR (accept*) OR (complicat*) OR (side effect*) OR (efficac*) OR (effect*) OR (feasib*) OR (prefer*) OR (satisf*) OR (feel*) or (autonom*) OR (delay*) OR (wait*) OR (experienc*) OR (uptake*) OR (time*) OR (distan*) OR (succe*) OR (impact) OR (opinion*) OR (best practice*) OR (capacity) OR (capab*) OR (competen*))

We searched Google (limited to the first 100 results) to identify grey literature and included known documents suggested by experts within the team and those identified through hand searches of the references of retrieved articles. Results from 01/01/2000 and 09/12/2022 that were peer reviewed research (journal articles and conference proceedings) theses or grey literature were included. We included papers where only a part of the investigation was relevant. We did not include books, book sections, cases, serials.

For peer review only

Appendix 2: Questions used to identify relevance

Five questions to operationalise a realist logic of analysis, adapted from Duddy et al (2021).

- Is there content that provides information on the context, mechanism or outcome of interventions? (CMO value)
- What do these data tell us about the context, mechanism or outcome of the interventions studies? (CMO outcomes)
- How should the CMO outcomes enable refinement of our initial programme theory? (refinement)

For peer review only

For peer review only

For peer review only - http://bmjopen.bmj.com/site/about/guidelines.xhtml

Appendix 3: Summary of papers included in the review

First Author	Date	Title	Source Type	Country	Setting	Participant Type	Number of Participants	Study Design	Is the paper relevant to the question?	Are the methods used appropriate?	Are the findings plausible?	Do conclusions support those of other studies?
Aiken, A. R. A	2018	Motivations and Experiences of People Seeking Medication Abortion Online in the United States	Original research paper	United States	Online	People seeking online medical abortion services	32	Qualitative interview study	Yes - paper explores motivations for considering self-managed abortion.	Yes - in-depth interviews were used to gather qualitative data on people's motivations and experiences of seeking online medical abortion.	Yes	Yes
Aiken, A.	2021b	Demand for self-managed online telemedicine abortion in eight European countries during the COVID-19 pandemic: a regression discontinuity analysis	Original research paper	Germany; Hungary; Italy; Malta; Netherlands; Northern Ireland; Portugal; Great Britain	Online	N/A	3915 requests for self-managed abortion to Women on Web (WoW)	Cross-sectional comparative analysis of requests for self-managed abortion pre- and post the Covid-19 pandemic	Yes - paper assesses whether the COVID-19 pandemic increased demand for self-managed medical abortion provided through online services and suggests reasons for trends in demand for online accessed abortion during the pandemic.	Yes - request rates in 8 countries are compared using regression discontinuity both before and after lockdown measures.	Yes	Yes
Atay	2021	Why women choose at-home abortion via teleconsultation in France: drivers of telemedicine abortion during and beyond the	Original research paper	France	Online	People accessing online medical abortion services	140	Mixed Methods (Cross-sectional survey of consultation data and email content analysis)	Highly - the paper explores drivers of at home, online supported, medical abortion during the pandemic and beyond.	Yes - the findings are based on appropriate analysis of consultations and emails extracted from an online medical abortion service to reveal the motivations and preferences of women seeking at home abortion.	Yes	Yes

For peer review only - http://bmjopen.bmj.com/site/about/guidelines.xhtml

		COVID-19 pandemic.										
Boydell	2021	Women's experiences of a telemedicine abortion service (up to 12 weeks) implemented during the coronavirus (COVID-19) pandemic: a qualitative evaluation	Original research paper	Scotland	Online	People accessing online medical abortion services	20	Qualitative interview study	Highly - paper explores women's experiences of accessing online medical abortion services.	Yes - the findings are based on appropriate use of qualitative interviews to explore women's experiences of telephone consultation; remote support; views on no pre-abortion ultrasound; and self-administration of abortion medications at home.	Yes	Yes
Ehrenreich	2019	Spatial dimensions of telemedicine and abortion access: a qualitative study of women's experiences	Original research paper	Utah, USA	Online	People accessing online medical abortion services	20	Qualitative interview study	Yes - the paper explores women's experiences of using online services for the first mandatory step when accessing medical abortion in Utah, an 'information visit'.	Yes - through in-depth interviews thoroughly explore experiences of using online services for information visits to fulfil abortion requirements.	Yes	Yes
Endler	2019	Telemedicine for medical abortion: a systematic review	Review	N/A	N/A	N/A	N/A	Systematic Review	Yes - the paper assesses the success rate, safety, and acceptability for women and providers of online medical abortion services.	Yes - a systematic review assessing a broad and significant number of relevant studies.	Yes	Yes
Erlank	2021	Acceptability of no-test medical abortion provided via telemedicine during Covid-19: analysis of patient-reported outcomes	Original research paper	United Kingdom	Online	People accessing online medical abortion services	1243	Qualitative survey study	Yes - the study reports patient-reported outcome measures assessing the quality of consultation, access to medicine after consultation, ability to manage the process at home and overall patient satisfaction.	Yes – early medical abortion patients invited to opt-in to a follow-up call post-procedure to answer clinical and satisfaction questions to measure acceptability of the service.	Yes	Yes

For peer review only - http://bmjopen.bmj.com/site/about/guidelines.xhtml

Eshre Capri Workshop Group	2017	Induced abortion	Workshop	N/A	N/A	N/A	N/A	N/A	Moderately - This paper presents commentary on induced abortion gathered at a workshop of experts in the field. The commentary included discussion of at home medical abortion with reference to online support.	N/A	N/A	Yes
Finch	2019	Impact of self-administration of misoprostol for early medical abortion: a prospective cohort study	Original research paper	Scotland	Home	People accessing abortion services both at home and at a facility	2430	Prospective observational study	Yes - the study explores the impact of Scotland's legalisation on home use of misoprostol for the purpose of early medical abortion on uptake and success rate, and on the provision of effective contraception on discharge.	Yes - prospective observational study followed the outcomes of two cohorts who received their abortion care, before and after the introduction of home use of misoprostol.	Yes	Yes
Fix	2020	At-home telemedicine for medical abortion in Australia: a qualitative study of patient experiences and recommendations	Original research paper	Australia	Online	People accessing online medical abortion services	24	Qualitative interview study	Highly - the study explores people's experiences obtaining a medical abortion through an online service and discusses people's reasons for choosing an online service.	Yes - in-depth interviews appropriately captures the experiences of people using the at-home telemedicine service.	Yes.	Yes
Gill	2019	Feasibility and Acceptability of a Mobile Technology Intervention to Support Postabortion Care in British	Original research paper	Canada	Abortion care facility	People attending an abortion care facility	58	Mixed-methods formative study (cross-sectional survey and semi-structured interviews)	Moderately - study aims to understand how people at surgical abortion clinics utilise their mobile phones to access health care information and their preferences for a mobile intervention	Yes - mixed-methods study appropriately explores a variety of aspects relevant to technology usage during abortion process.	Yes	Yes

For peer review only - http://bmjopen.bmj.com/site/about/guidelines.xhtml

		Columbia: Phase 1							that supports follow-up care.			
Gill & Norman	2018	Telemedicine and medical abortion: dispelling safety myths, with facts	Editorial	N/A	N/A	N/A	N/A	Editorial commentary on recent research regarding online medical abortion services	Moderately - This paper presents commentary on recent research regarding online medical abortion services.	N/A	N/A	Yes
Goldman	2021	Transcutaneous Electrical Nerve Stimulation to Reduce Pain With Medication Abortion: A Randomized Controlled Trial	Original research paper	N/A	Abortion care facility	People undergoing medical abortion.	251	Randomised controlled trial (RCT)	Yes - evaluates whether high-frequency transcutaneous electrical nerve stimulation reduces experiences of pain during medical abortion.	Yes – randomized control trial shows that participants who received high-frequency transcutaneous electrical nerve stimulation after medical abortion had significantly lower posttreatment pain scored compared to those that received a sham procedure.	Yes	Yes
Hamoda	2005	The acceptability of home medical abortion to women in UK settings	Original research paper	England and Scotland	Home	People seeking hospital based abortion care	553	Qualitative survey study	Highly - the study explores the acceptability of home medical abortion to people UK settings including managing pain and bleeding at home.	Yes - self-complete questionnaires adequately measure people's views on the acceptability of medical abortion at home.	Yes	Yes
Harden	2021	Women's experiences of self-administration of misoprostol at home as part of early medical abortion: a qualitative evaluation	Original research paper	Scotland	Home	People that had recently undergone early medical abortion	20	Qualitative interview study	Highly - the study explores the experiences of people who accessed at home medical abortion and reported benefits of at home administration including flexibility, privacy and comfort.	Yes - qualitative data is collected exploring what people desire and valued during the process.	Yes	Yes
Heath	2019	A comparison of termination of pregnancy procedures: Patient choice,	Original research paper	Sweden	Abortion care facility	People requesting an abortion		A mixed-method prospective comparative study	Yes	Yes	Yes	Yes

For peer review only - http://bmjopen.bmj.com/site/about/guidelines.xhtml

		emotional impact and satisfaction with care										
Hedqvist	2016	Women's experiences of having an early medical abortion at home	Original research paper	Sweden	Home	People accessing medical abortion at home	119	Cross-sectional (semi-structured telephone interviews)	Highly - paper investigates people's experiences of having a medical abortion at home including experiences of pain and bleeding.	Yes - descriptive and comparative design enable an in-depth and balanced conclusion. Study also investigates differences between groups of people.	Yes	Yes
Hoggart and Berer	2021	Making the case for supported self-managed medical abortion as an option for the future	Editorial	N/A	Online	N/A	N/A	Editorial commentary on recent research regarding online medical abortion services	Moderately – this paper reviews recent research on online medical abortion services highlighting benefits and pointing out need for further development of services, suggesting a need for access to 24 hours support.	N/A	N/A	Yes
Iyer	2021	Preferences for contraceptive counselling and access among abortion patients at an independent clinic in Texas	Original research paper	USA	Abortion care facility	People attending an abortion care facility	181	Cross-sectional survey study	Yes - study explores preferences for contraceptive counselling and access to contraception.	Yes - self-administered surveys used as an appropriate method considering legally restrictive setting and associated stigma.	Yes	Yes
Kavanagh	2011	Patients' attitudes and experiences related to receiving contraception during abortion care	Original research paper	USA	Abortion care facility	People accessing abortion at an abortion care facility	542	Cross-sectional survey study	Yes - study documents attitudes towards contraceptive services and identifies participants characteristics associated with desire for contraception and interest in LARC.	Yes - appropriate methods for exploring attitudes and characteristics associated with specific needs relevant to contraceptive use.	Yes	Yes
Kero	2010	Home abortion - experiences of	Original research paper	Sweden	Home	Couples - where the male partner had been	23 couples	Qualitative interview study	Moderately - the paper explores the male partner's experience of being	Yes - qualitative method ensures high detailed analysis in experiences of	Yes	Yes

For peer review only - http://bmjopen.bmj.com/site/about/guidelines.xhtml

		male involvement				present when their female partner had had an at home medical abortion			present and supporting their partner during an induced home abortion.	both the pregnant woman and her partner.		
Killinger	2022	Why women choose abortion through telemedicine outside the formal health sector in Germany: a mixed-methods study	Original research paper	Germany	Online	People accessing an online medical abortion service	1090	Cross-sectional study analysing data from online consultations	Yes - the paper aims to understand the motivations and barriers to access for people who choose online abortion services outside of the formal health sector.	Yes - a cross-sectional study of data contained in online consultations and a content analysis of over 100 email texts.	Yes	Yes
Levine and Cameron	2009	Women's preferences for method of abortion and management of miscarriage	Original research paper	United Kingdom	Abortion care facility	People undergoing medical abortion, surgical abortion and surgical management of miscarriage	148	Cross-sectional survey study	Yes - the study explores views on medical treatment at home, and surgery under local anaesthesia, to determine whether new services should be developed.	Moderately - small sample size of self-administered anonymous questionnaires.	Yes	Yes
Loeber	2016	Contraceptive counselling for women with multiple unintended pregnancies: the abortion client's perspective	Original research paper	Netherland s	Abortion care facility	Survey – People attending an abortion care facility<br><br>Interviews – People attending an abortion care facility that had had more than three unintended pregnancies	212	Mixed methods (quantitative survey and semi-structured interviews)	Yes - study explores views on contraception use after induced abortion and post-abortion contraceptive counselling	Yes - mixed method approach appropriately explores experiences of post-abortion contraception and contraception counselling both in-depth and across a larger sample.	Yes	Yes

For peer review only

For peer review only - http://bmjopen.bmj.com/site/about/guidelines.xhtml

Lohr	2018	Telephone or integrated contraception counselling before abortion: impact on method choice and receipt	Original research paper	United Kingdom	Abortion care facility	People who had accessed abortion at an abortion care facility	18,573	Cross-sectional study of de-identified data	Yes - paper compares the characteristics of people who chose contraception counselling either over the telephone and separate from abortion consultation or face-to-face and integrated into consultation.	Yes - very large sample size which thoroughly explores demographic characteristics and contraceptive method choice.	Yes	Yes
Lokeland	2014	Medical abortion with mifepristone and home administration of misprostol up to 63 days' gestation	Original research paper	Norway	Abortion care facility	People seeking an at home medical abortion before 63 days'	1,018	Observational study	Yes - paper evaluates the acceptability of medical abortion at home, including pain and bleeding.	Yes – a large sample size of people experiencing at home abortion with follow up telephone recorded to asses bleeding, pain and acceptability.	Yes	Yes
Low	2021	Women's experiences of self-referral to an abortion service: qualitative study	Original research paper	Scotland	Abortion care service	People attending an abortion care service	21	Qualitative interview study	Yes - this study evaluates a self-referral service to abortion services by investigating its impact on people's experiences of the referral process.	Yes - semi-structured interviews to focus on people's experiences of the referral process.	Yes	Yes
Makenzius	2012	Autonomy and dependence - experiences of home abortion, contraception and prevention	Original research paper	Sweden	Home	People who have experienced an at-home abortion	37	Qualitative interview study	Yes - study explores people's experiences and needs related to care in the context of a home abortion.	Yes – interviews capture patients at-home abortion experiences and needs which fall under themes of autonomy (choice of at-home abortion, increase of privacy and control) and dependence (desire to be treated with empathy by health care workers and receive adequate information).	Yes	Yes
Matulich	2014	Understanding women's desires for contraceptive counselling at the time of	Original research paper	USA	Abortion care facility	People receiving surgical abortion at an abortion care facility	199	Qualitative survey study	Yes - paper investigates whether or not people presenting for a first-trimester surgical abortion want to	Yes - survey obtained demographic information and inquired about desire for contraceptive counselling.	Yes	Yes

For peer review only - http://bmjopen.bmj.com/site/about/guidelines.xhtml

For peer review only

		first-trimester surgical abortion							discuss contraception on the day of their procedure.			
Norman	2014	Access to Complex Abortion Care Service and Planning Improved through a Toll-Free Telephone Resource Line	Original research paper	Canada	Abortion care facility	N/A	N/A	Review of service provision model	Yes - study presents a review of a service provision model which has provided improved access to abortion care through a toll-free telephone abortion access and counselling service.	Yes - a descriptive analysis appropriately reports on the development and delivery of this service.	Yes	Yes
Oppegaard	2015	Clinical follow-up compared with self-assessment of outcome after medical abortion: a multicentre, non-inferiority, randomised, controlled trial	Original research paper	Austria; Finland; Norway; Sweden	Abortion care services	People requesting medical abortion	924	Randomised, controlled, non-inferiority trial	Yes - study compares clinical assessment with self-assessment of abortion outcome.	Yes - assigned people in a 1:1 ratio to attend routine clinical follow-up or to self-assess outcome at home with a semi-quantitative urine human chorionic gonadotropin test 1-3 weeks after abortion.	Yes	Yes
Pohjoranta	2018	Predicting poor compliance with follow-up and intrauterine contraception services after medical termination of pregnancy	Original research paper	Finland	Abortion care facility	People undergoing a medical abortion	605	RCT	Moderately - paper assesses factors associated with non-compliance with post-abortion services and evaluates differences in rates of attendance and intrauterine device insertion.	Yes – the study compared the intervention group booked to have IUD insertion 1-4 weeks after medical abortion and women in control group were advised to contact their PHC for follow-up and IUD insertion.	Yes	Yes
Pohjoranta	2020	Early provision of intrauterine contraception as part of abortion care - 5-year results of a randomised controlled trial	Original research paper	Finland	Abortion care facility	People undergoing an induced abortion	748	RCT	Moderately - paper investigates whether the incidence of subsequent termination of pregnancy can be reduced by providing IUD as part of the abortion service.	Yes – RCT assesses the effectiveness of early comprehensive provision of IUD after induced abortion. Intervention group provided with an IUD during surgical abortion and women in control group advised to	Yes	Yes

For peer review only - http://bmjopen.bmj.com/site/about/guidelines.xhtml

										contact primary healthcare for follow-up and IUD insertion.		
Powell-Jackson	2010	Benefits of using a digital video disk for providing information about abortion to women requesting termination of pregnancy	Original research paper	Scotland	Abortion care facility	People attending an abortion care facility and facility staff	226	Comparative cross-sectional survey design	Yes - paper examines the benefits of using a DVD for providing information about abortion.	Yes - questionnaires are used to appropriately measure people's satisfaction with the information received through the DVD and what more/less they would want it to include.	Yes	Yes
Purcell	2017	Self-managment of first trimester medical termination of pregnancy: a qualitative study of women's experiences	Original research paper	Scotland	Abortion care facility	People who are administered a medical abortion at a facility and return home to complete termination	44	Qualitative interview study	Yes - explores people's experiences of returning home to complete medical abortion, including self-monitoring of treatment process.	Yes - in-depth interviews to assess people's experiences in detail.	Yes	Yes
Rafferty & Longbons	2021	#AbortionChangesYou: A Case Study to Understand the Communicative Tensions in Women's Medication Abortion Narratives	Original research paper	United States	Online	People who have had a medical abortion	98 blog posts	Case study	Yes - study analyses people's narratives after having had a medication abortion though blogs posted on a website - Abortion Changes You.	Yes - using a case study approach enabled the study to explore multiple perspectives rooted in specific contexts.	Yes	Yes
Raymond	2018	Low-sensitivity urine pregnancy testing to assess medical abortion outcome: A systematic review	Review	N/A	N/A	N/A	N/A	Systematic review	Yes - the study explores data on the accuracy and acceptability of a strategy for identifying ongoing pregnancy after medical abortion treatment using a low-sensitivity pregnancy test (LSPT).	Moderately - A more comprehensive search across other databases could strengthen study.	Yes	Yes

For peer review only - http://bmjopen.bmj.com/site/about/guidelines.xhtml

Raymond	2017	Self-assessment of medical abortion outcome using symptoms and home pregnancy testing	Original research paper	United States	Abortion care facility	People undergoing medical abortion	343	Intervention study	Highly - study evaluates compliance with a strategy to enable medical abortion patients to assess treatment outcome on their own and decide whether to seek clinical follow-up.	Yes - appropriate methodology to clinically assess women's compliance to using a pregnancy test after medical abortion and women's ability to self-assess treatment outcome.	Yes	Yes
Reynolds-Wright	2020	Pain management for medical abortion before 14 weeks' gestation	Review	N/A	N/A	N/A	N/A	Systematic review	Yes - paper reviews pain relief regimens for the management of medical abortion.	Yes - wide variety of databases, conference abstracts and organisations systematically searched and analysed.	Yes	No
Reynolds-Wright	2021	Telemedicine medical abortion at home under 12 weeks' gestation: a prospective observational cohort study during the COVID-19 pandemic	Original research paper	Scotland	Online/Home	People choosing online medical abortion services	663	Prospective cohort study	Yes - the paper determines the efficacy of an online at-home medical abortion service, noting any complications after treatment and acceptability of care.	Yes - large sample size. Questionnaires and hospital database analyses allows for in-depth review of efficacy, complications and acceptability.	Yes	Yes
Robson	2009	Randomised preference trial of medical versus surgical termination of pregnancy less than 14 weeks' gestation (TOPS)	Original research paper	United Kingdom	Abortion care facility	People undergoing induced abortion and people attending a contraceptive and sexual health clinic	1,877	Randomised preference trial and economic evaluation with qualitative sub study	Yes - the paper explores the acceptability, efficacy and costs of medical abortion compared with surgical abortion and aims to understand people's decision-making processes and experiences.	Yes – participants are either randomly assigned a procedure type (with consent to random allocation) or choose their procedure type to evaluate whether in people without prior preference, acceptability of medical and surgical abortion is the same. A qualitative sub study explores people's preferences and decision making.	Yes	Moderately
Rowlands & Wale	2020	A Constructivist	Constructivist Vision	N/A	N/A	N/A	N/A	Constructivist vision	Moderately - This paper provides	N/A	N/A	Yes

For peer review only - http://bmjopen.bmj.com/site/about/guidelines.xhtml

		Vision of the First-Trimester Abortion Experience						commentary on how a high-quality abortion experience might be achieved in the first trimester if regulatory laws were dismissed.	commentary on recent research on induced abortion in the first trimester within the framework of a future vision of state obligation to provide high quality abortion care. This paper also provides an evidence-based set of ingredients that facilitate a positive high-quality abortion experience.			
Schmidt-Hansen	2020	Follow-up strategies to confirm the success of medical abortion of pregnancies up to 10 weeks' gestation: a systematic review with meta-analyses	Review	N/A	N/A	N/A	N/A	Systematic review	Yes - the review compares the effectiveness, safety, and acceptability of in-clinic and remote/self-assessment for confirming the success of medical abortion.	Yes - systematic review including RCT's from 2000 onward to ensure a broad range of studies captured. Quality of evidence compromised by small event rates, lack of blinding, and high attrition rates.	Yes	Yes
Sherman	2017	Providing experiential information on early medical abortion: a qualitative evaluation of an animated personal account, *Lara's Story*	Original research paper	Scotland	Abortion care facility	People who have had a medical abortion	13	Qualitative interview study	Yes - the study evaluates the views on an animated film, and its potential usefulness in providing information on medical abortion.	Yes - the use of interviews allows people to report their full experience and opinion in detail, also providing feedback or criticism.	Yes	Yes
Smith	2019	Current barriers, facilitators and future improvements to advance	Original research paper	Edinburgh, Scotland	Abortion care facility	People seeking induced abortion	154	Cross-sectional survey study	Yes - the study explores current barriers access to care, and what future improvements should	Yes - self-administered anonymous questionnaires to capture experiences.	Yes	Yes

For peer review only - http://bmjopen.bmj.com/site/about/guidelines.xhtml

		quality of abortion care: views of women							be implemented to abortion care services			
Smith	2017a	Women's views and experiences of a mobile phone-based intervention to support post-abortion contraception in Cambodia	Original research paper	Cambodia	Abortion care facility	People who had received a mobile phone based post-abortion contraception counselling service.	15	Qualitative interview study	Yes - the study assesses views and experiences of receiving the MObile Technology for Improved Family Planning (MOTIF) intervention, which aims to increase post-abortion contraception.	Yes - semi-structured interviews to capture in-depth data. Small sample size may be a limitation.	Yes	Yes
Smith	2017b	Process evaluation of a mobile phone-based intervention to support post-abortion contraception in Cambodia	Original research paper	Cambodia	Abortion care facility	People who had received a mobile phone based post-abortion contraception counselling service.	249	Process evaluation	Yes - the study assesses participants' interaction with the MOTIF intervention.	Yes - thorough process evaluation to assess associations with the intervention and post-abortion contraception use.	Yes	Yes
Upadhyay, U. D.	2021	Safety and Efficacy of Telehealth Medication Abortions in the US During the COVID-19 Pandemic	Original research paper	USA	Online	People accessing online medical abortion	141	Retrospective cohort study	Yes - the study assesses the safety and efficacy outcomes of a online medical abortion model.	Yes – appropriate methodology for assessing an online medical abortion service however the small sample size with some loss to follow-up, and thus some adverse events and ongoing pregnancies may have been undetected.	Yes	Yes
Whitehouse	2021	*It's a small bit of advice, but actually on the day, made such a difference...*: perceptions of quality in abortion care in England and Wales	Original research paper	England; Wales	Abortion care facility	People who have had an abortion at a facility withing the last 6 months	24	Qualitative interview study	Yes - the paper explores participants' experiences and views on abortion quality of care.	Yes - interviews are an appropriate methodology to assess experiences.	Yes	Yes

For peer review only - http://bmjopen.bmj.com/site/about/guidelines.xhtml

Wieβe	2008	Access to Abortion: What Women Want From Abortion Services	Original research paper	Canada	Abortion care facility	People seeking an abortion	441	Qualitative survey and interview study	Moderately - the paper identifies barriers to access for women seeking induced abortion at a facility.	Yes - questionnaire accurately explores demographic information, perceived barriers to access, and what women desire from abortion services. Interviews captured in-depth analysis to access questions.	Yes	Yes

For peer review only

For peer review only - http://bmjopen.bmj.com/site/about/guidelines.xhtml

Reporting checklist for systematic review (with or without a meta-analysis).

Based on the PRISMA guidelines.

Instructions to authors

Complete this checklist by entering the page numbers from your manuscript where readers will find each of the items listed below.

Your article may not currently address all the items on the checklist. Please modify your text to include the missing information. If you are certain that an item does not apply, please write "n/a" and provide a short explanation.

Upload your completed checklist as an extra file when you submit to a journal.

In your methods section, say that you used the PRISMAreporting guidelines, and cite them as:

Page MJ, McKenzie JE, Bossuyt PM, Boutron I, Hoffmann TC, Mulrow CD, Shamseer L, Tetzlaff JM, Akl EA, Brennan SE, Chou R, Glanville J, Grimshaw JM, Hróbjartsson A, Lalu MM, Li T, Loder EW, Mayo-Wilson E, McDonald S, McGuinness LA, Stewart LA, Thomas J, Tricco AC, Welch VA, Whiting P, Moher D. The PRISMA 2020 statement: An updated guideline for reporting systematic reviews

For peer review only

		Reporting Item	Page Number
**Title**			
Title	#1	Identify the report as a systematic review	1
**Abstract**			
Abstract	#2	Report an abstract addressing each item in the PRISMA 2020 for Abstracts checklist	2
**Introduction**			
Background/rationale	#3	Describe the rationale for the review in the context of existing knowledge	3
Objectives	#4	Provide an explicit statement of the objective(s) or question(s) the review addresses	3
**Methods**			

Eligibility criteria	#5	Specify the inclusion and exclusion criteria for the review and how studies were grouped for the syntheses	5-6
Information sources	#6	Specify all databases, registers, websites, organisations, reference lists, and other sources searched or consulted to identify studies. Specify the date when each source was last searched or consulted	Appendix 1
Search strategy	#7	Present the full search strategies for all databases, registers, and websites, including any filters and limits used	Appendix 1
Selection process	#8	Specify the methods used to decide whether a study met the inclusion criteria of the review, including how many reviewers screened each record and each report retrieved, whether they worked independently, and, if applicable, details of automation tools used in the process	6
Data collection process	#9	Specify the methods used to collect data from reports, including how many reviewers collected data from each report, whether they worked independently, any processes for obtaining or confirming data from study investigators, and, if applicable, details of automation tools used in the process	6-7
Data items	#10a	List and define all outcomes for which data were sought. Specify whether all results that were compatible with each outcome domain in each study were sought (for example, for all measures, time points, analyses), and, if not, the methods used to decide which results to collect	7
Study risk of bias assessment	#11	Specify the methods used to assess risk of bias in the included studies, including details of the tool(s) used, how many reviewers assessed each study and whether they worked independently, and, if applicable, details of automation tools used in the process	N/a
Effect measures	#12	Specify for each outcome the effect measure(s) (such as risk ratio, mean difference) used in the synthesis or presentation of results	7
Synthesis methods	#13a	Describe the processes used to decide which studies were eligible for each synthesis (such as tabulating the study intervention characteristics and comparing against the planned groups for each synthesis (item #5))	7
Synthesis methods	#13b	Describe any methods required to prepare the data for presentation or synthesis, such as handling of missing summary statistics or	7

For peer review only

		data conversions	
Synthesis methods	#13c	Describe any methods used to tabulate or visually display results of individual studies and syntheses	7
Synthesis methods	#13d	Describe any methods used to synthesise results and provide a rationale for the choice(s). If meta-analysis was performed, describe the model(s), method(s) to identify the presence and extent of statistical heterogeneity, and software package(s) used	7
Synthesis methods	#13e	Describe any methods used to explore possible causes of heterogeneity among study results (such as subgroup analysis, meta-regression)	7
Synthesis methods	#13f	Describe any sensitivity analyses conducted to assess robustness of the synthesised results	7
Reporting bias assessment	#14	Describe any methods used to assess risk of bias due to missing results in a synthesis (arising from reporting biases)	n/a
Certainty assessment	#15	Describe any methods used to assess certainty (or confidence) in the body of evidence for an outcome	7
Data items	#10b	List and define all other variables for which data were sought (such as participant and intervention characteristics, funding sources). Describe any assumptions made about any missing or unclear information	7
**Results**			
Study selection	#16a	Describe the results of the search and selection process, from the number of records identified in the search to the number of studies included in the review, ideally using a flow diagram (http://www.prisma-statement.org/PRISMAStatement/FlowDiagram)	8-9
Study selection	#16b	Cite studies that might appear to meet the inclusion criteria, but which were excluded, and explain why they were excluded	10
Study characteristics	#17	Cite each included study and present its characteristics	Appendix 3
Risk of bias in studies	#18	Present assessments of risk of bias for each included study	N/a
Results of individual	#19	For all outcomes, present for each study (a) summary statistics for	Appendix

For peer review only

studies		each group (where appropriate) and (b) an effect estimate and its precision (such as confidence/credible interval), ideally using structured tables or plots	3
Results of syntheses	#20a	For each synthesis, briefly summarise the characteristics and risk of bias among contributing studies	10-14
Results of syntheses	#20b	Present results of all statistical syntheses conducted. If meta-analysis was done, present for each the summary estimate and its precision (such as confidence/credible interval) and measures of statistical heterogeneity. If comparing groups, describe the direction of the effect	N/a
Results of syntheses	#20c	Present results of all investigations of possible causes of heterogeneity among study results	10-14
Results of syntheses	#20d	Present results of all sensitivity analyses conducted to assess the robustness of the synthesised results	n/a
Risk of reporting biases in syntheses	#21	Present assessments of risk of bias due to missing results (arising from reporting biases) for each synthesis assessed	16
Certainty of evidence	#22	Present assessments of certainty (or confidence) in the body of evidence for each outcome assessed	6
**Discussion**			
Results in context	#23a	Provide a general interpretation of the results in the context of other evidence	15
Limitations of included studies	#23b	Discuss any limitations of the evidence included in the review	15
Limitations of the review methods	#23c	Discuss any limitations of the review processes used	16
Implications	#23d	Discuss implications of the results for practice, policy, and future research	16
**Other information**			
Registration and protocol	#24a	Provide registration information for the review, including register name and registration number, or state that the review was not registered	3
Registration and	#24b	Indicate where the review protocol can be accessed, or state that a	3

For peer review only

protocol		protocol was not prepared	
Registration and protocol	#24c	Describe and explain any amendments to information provided at registration or in the protocol	n/a
Support	#25	Describe sources of financial or non-financial support for the review, and the role of the funders or sponsors in the review	3
Competing interests	#26	Declare any competing interests of review authors	3
Availability of data, code, and other materials	#27	Report which of the following are publicly available and where they can be found: template data collection forms; data extracted from included studies; data used for all analyses; analytic code; any other materials used in the review	3

Notes:

- 6: Appendix 1
- 7: Appendix 1
- 17: Appendix 3 The PRISMA checklist is distributed under the terms of the Creative Commons Attribution License CC-BY. This checklist was completed on 13. July 2022 using https://www.goodreports.org/, a tool made by the EQUATOR Network in collaboration with Penelope.ai

For peer review only

BMJ Open

Improving experience of medical abortion at home in a changing therapeutic, technological and regulatory landscape: a realist review

Journal:	*BMJ Open*
Manuscript ID	bmjopen-2022-066650.R1
Article Type:	Original research
Date Submitted by the Author:	04-Oct-2022
Complete List of Authors:	Baraitser, Paula; King's College London School of Population Health and Environmental Sciences<br>Free, Caroline; London School of Hygiene & Tropical Medicine<br>Norman, Wendy; The University of British Columbia, Dept of Family Practice; London School of Hygiene and Tropical Medicine Faculty of Public Health and Policy, Public Health, Environments & Society<br>Lewandowska, Maria; London School of Hygiene and Tropical Medicine Faculty of Public Health and Policy<br>Meiksin, Rebecca; London School of Hygiene and Tropical Medicine Faculty of Public Health and Policy<br>Palmer, Melissa; London School of Hygiene and Tropical Medicine Faculty of Public Health and Policy<br>Scott , Rachel ; London School of Hygiene and Tropical Medicine Faculty of Public Health and Policy<br>French, Rebecca ; London School of Hygiene and Tropical Medicine Faculty of Public Health and Policy<br>Wellings, Kaye; London School of Hygiene & Tropical Medicine<br>Ivory, Alice; London School of Hygiene & Tropical Medicine, DPH<br>Wong, Geoff; Oxford University, Nuffield Department of Primary Care Health Sciences
**Primary Subject Heading**:	Reproductive medicine
Secondary Subject Heading:	Sexual health, Obstetrics and gynaecology
Keywords:	Telemedicine < BIOTECHNOLOGY & BIOINFORMATICS, SEXUAL MEDICINE, REPRODUCTIVE MEDICINE

SCHOLARONE™
Manuscripts

I, the Submitting Author has the right to grant and does grant on behalf of all authors of the Work (as defined in the below author licence), an exclusive licence and/or a non-exclusive licence for contributions from authors who are: i) UK Crown employees; ii) where BMJ has agreed a CC-BY licence shall apply, and/or iii) in accordance with the terms applicable for US Federal Government officers or employees acting as part of their official duties; on a worldwide, perpetual, irrevocable, royalty-free basis to BMJ Publishing Group Ltd ("BMJ") its licensees and where the relevant Journal is co-owned by BMJ to the co-owners of the Journal, to publish the Work in this journal and any other BMJ products and to exploit all rights, as set out in our licence.

The Submitting Author accepts and understands that any supply made under these terms is made by BMJ to the Submitting Author unless you are acting as an employee on behalf of your employer or a postgraduate student of an affiliated institution which is paying any applicable article publishing charge ("APC") for Open Access articles. Where the Submitting Author wishes to make the Work available on an Open Access basis (and intends to pay the relevant APC), the terms of reuse of such Open Access shall be governed by a Creative Commons licence – details of these licences and which Creative Commons licence will apply to this Work are set out in our licence referred to above.

Other than as permitted in any relevant BMJ Author's Self Archiving Policies, I confirm this Work has not been accepted for publication elsewhere, is not being considered for publication elsewhere and does not duplicate material already published. I confirm all authors consent to publication of this Work and authorise the granting of this licence.

For peer review only

Improving experience of medical abortion at home in a changing therapeutic, technological and regulatory landscape: a realist review

Authors
Paula Baraitser[1], Caroline Free[2], Wendy Norman[3,4], Maria Lewandowska[4*], Rebecca Meiksin[4], Melissa Palmer[4], Rachel Scott[4], Rebecca French[4], Kaye Wellings[4], Alice Ivory[2], Geoff Wong[5], The SACHA study team.

[1] School of Population Health & Environmental Sciences, King's College London, London, UK
[2] Faculty of Epidemiology and Population Health, London School of Hygiene & Tropical Medicine
[3] Department of Family Practice, University of British Columbia, Vancouver, British Columbia, Canada.
[4] Faculty of Public Health and Policy, London School of Hygiene & Tropical Medicine
[5] Nuffield Department of Primary Care Health Sciences, University of Oxford, Oxford, UK

*Corresponding author:
Maria Lewandowska, maria.lewandowska@lshtm.ac.uk 020 7927 2761
London School of Hygiene and Tropical Medicine 15-17 Tavistock Place, WC1H 9SH

Paula Baraister paula.baraitser@kcl.ac.uk
Caroline Free caroline.free@lshtm.ac.uk
Wendy Norman wendy.norman@lshtm.ac.uk
Maria Lewandowska maria.lewandowska@lshtm.ac.uk
Rebecca Meiksin rebecca.meiksin@lshtm.ac.uk
Melissa Palmer melissa.palmer@lshtm.ac.uk
Rachel Scott Rachel.scott@lshtm.ac.uk
Rebecca French rebecca.french@lshtm.ac.uk
Kaye Wellings kaye.wellings@lshtm.ac.uk
Alice Ivory alice.ivory@lshtm.ac.uk
Geoffrey Wong Geoffrey.wong@phc.ox.ac.uk

Target journal
BMJ Open

Word count
4199

Keywords
Abortion, telemedicine, reproductive health, self-care

For peer review only

For peer review only - http://bmjopen.bmj.com/site/about/guidelines.xhtml

Abstract

Objective:

To inform UK service development to support medical abortion at home, appropriate for person and context.

Design:

Realist review

Setting/participants:

Peer-reviewed literature from 01/01/2000 to 09/12/2021, describing interventions or models of home abortion care. Participants included people seeking or having had an abortion.

Interventions:

Interventions and new models of abortion care relevant to the UK.

Outcome measures:

Causal explanations, in the form of context-mechanism-outcome configurations, to test and develop our realist program theory.

Results:

We identified 12,401 abstracts, selecting 944 for full text assessment. Our final review included 50 papers. Medical abortion at home is safe, effective and acceptable to most, but clinical pathways and user experience are variable and a minority would not choose this method again. Having a choice of abortion location remains essential, as some people are unable to have a medical abortion at home. Choice of place of abortion (home or clinical setting) was influenced by service factors (appointment number, timing, and wait-times), personal responsibilities (caring/work commitments), geography (travel time/distance), relationships (need for secrecy) and desire for awareness/involvement in the process. We found experiences could be improved by offering: an option for self-referral through a telemedicine consultation, realistic information on range of experiences, opportunities to personalise the process, improved pain relief, and choice of when and how to discuss contraception.

Conclusions

Acknowledging the work done by patients when moving medical abortion care from clinic to home is important. Patients may benefit from support to: prepare a space, manage privacy and work/caring obligations, decide when/how to take medications, understand what is normal, assess experience and decide when and how to ask for help. The transition of this complex intervention when delivered outside healthcare environments could be supported by strategies that reduce surprise or anxiety, enabling preparation and a sense of control.

Strengths and limitations

- Strengths:
    - We present a systematic and transparent approach to the realist review, which we conducted in accordance with the RAMESES standards (27);
    - A broad range of content expertise from our authorship team, patients and the public informed the review
- Limitations:
    - Our analysis was conducted only on publicly accessible literature, located through recognised research databases and Google;
    - We found gaps in the evidence and have highlighted these in our conclusions;

  Our findings are intended to be relevant to settings where abortion is legal, and primarily where it is delivered as part of a universal health care system

For peer review only - http://bmjopen.bmj.com/site/about/guidelines.xhtml

Background

Changes in the therapeutic, technological and regulatory landscape are impacting abortion care and changing access to medical abortion at home. Medical abortion requires two medications, mifepristone (a competitive progestogen receptor antagonist) and the synthetic prostaglandin, misoprostol. Since mifepristone was first licenced for use in Great Britain in 1991, the proportion of all abortions that are medical abortions has increased steadily with a more recent shift towards medical abortion at home, where both the mifepristone and misoprostol or the misoprostol alone are taken at home. During the COVID 19 pandemic these trends were accelerated and in 2020, 85% of all abortions completed in England and Wales, and 88% of those in Scotland, were medical abortions at home. [1,2] These developments reflect similar changes in Australia, Canada and Europe. [3-7]

Medical abortion at home is safe, effective and acceptable and clinical guidance on some elements, such as doses of abortion medications, are consistent. [7-21] However, significant variation in other elements of clinical pathways remain as providers test new approaches to providing information, support, pain relief and follow up. [22] It is important to note that a minority (17 – 34%) of people who participate in research on medical abortion at home would not choose this method if having an abortion again. [12, 23-25] We are interested in the evidence to inform optimal configuration of medical abortion services at home and particularly in the differences in experience between populations and contexts.

<u>Terminology and settings</u>

Our findings relate to settings where abortion is legal and where there is a choice of abortion method and setting. Recommendations may be different in settings where abortion is illegal, where mifepristone is not available, where medication combinations or doses are used that are nt evidence based or where surgical options are not available or not safe. Women and people with other gender identities may have abortions. We have used the terms 'people who have an abortion' or 'people' to describe our population of interest.

<u>Objectives</u>

- To synthesise evidence on user experience of medical abortion at home
- To develop a realist programme theory to explain what interventions improve user experience for whom and in what context.
- To use this programme theory to develop recommendations for service providers and those having medical abortions at home

<u>Review question</u>

- What support, for whom, and in what circumstances could improve the experience of people who have medical abortion at home?

For peer review only - http://bmjopen.bmj.com/site/about/guidelines.xhtml

Methods

The SACHA study brings together over 20 researchers from seven countries to create an evidence base to inform health service configuration for abortion provision in the UK (https://www.lshtm.ac.uk/research/centres-projects-groups/sacha#welcome). This review is part of this programme of work and draws on the expertise of the team.

In the literature review component of the study, we sought to investigate novel models of abortion care addressing current therapeutic, technological, and regulatory trends, which would be relevant to the UK in the next 5 years. We subsequently divided this work between two reviews: a scoping review discussing the healthcare practitioner and system preparedness for abortion provision, which is currently in progress, and this realist review, focused on improving the experience of medical abortion at home.

We conceptualised medical abortion as a complex process that has context-sensitive outcomes which may be variably delivered and experienced. A realist approach enabled us to both account for and explain the influence of context on this variation. [26] Our protocol was registered with Prospero https://www.crd.york.ac.uk/prospero/display_record.php?RecordID=225307 and we followed the steps which are commonly used in realist reviews [27, 28] - location of existing theories, evidence search and document selection, data extraction, data synthesis and development of a refined programme theory.

We used the PRISMA checklist when writing our report.[1]

Location of existing theories

A realist approach to understanding interventions (in our case home medical abortion) proposes that any intervention is underpinned by one or more theories (that may be implicit or explicit) in the design of the intervention. This theory sets out how the designers anticipate their intervention will work. In a realist review this understanding is captured via an initial programme theory. Our initial programme theory was developed with input from the SACHA team. We charted the stages of home medical abortion from a user perspective to understand what type of support might be required from the health care system at each stage (Figure 1). These stages within a user journey were used to structure our analysis.

Figure 1 : Stages of the medical abortion process (initial theory of change)

(attached)

Searching for evidence and document selection

Our evidence search strategy started with a comprehensive literature search designed to answer a broader question for the SACHA Study: ‘Interventions of models of abortion care/provision: addressing current therapeutic, technological and regulatory trends which would be relevant to or feasible in the UK in the next 5 years.’ This search was designed to retrieve papers that would likely be relevant to both this realist review and a scoping review. The databases and search terms for this search are listed in appendix 1 and the inclusion and exclusion criteria are listed in Table 1. The search dates were 01/01/2000 - 09/12/2022.

[1] Page MJ, McKenzie JE, Bossuyt PM, Boutron I, Hoffmann TC, Mulrow CD, Shamseer L, Tetzlaff JM, Akl EA, Brennan SE, Chou R, Glanville J, Grimshaw JM, Hróbjartsson A, Lalu MM, Li T, Loder EW, Mayo-Wilson E, McDonald S, McGuinness LA, Stewart LA, Thomas J, Tricco AC, Welch VA, Whiting P, Moher D. The PRISMA 2020 statement: An updated guideline for reporting systematic reviews.

For peer review only - http://bmjopen.bmj.com/site/about/guidelines.xhtml

Table 1: Inclusion/exclusion criteria for scoping review.

	Included	Excluded
Interventions		
	Interventions of models of abortion care/provision: addressing current therapeutic, technological and regulatory trends which would be relevant to, or feasible in the UK in the next 5 years	Interventions aiming to legalise abortion, mitigate the effects of illegal abortion or address the financial aspects of abortion access. Interventions relevant to abortion care in unregulated/poorly regulated contexts. Pharmacological studies.
Populations	People seeking/having had an abortion, those accompanying someone through an abortion process, health care workers reporting experience of/attitudes towards abortion provision.	

Search results were imported into an electronic reference manager Covidence (https://www.covidence.org/) and screened in two stages. The first screen was based on title and abstract with a sample double-screened to check for consistency and discussion of studies of uncertain eligibility to reach consensus. The remainder of papers at the abstract and title stage and all those at the full text stage were single screened. The full text of all included documents were uploaded into a second electronic reference management system (Endnote).

We screened the papers using the WHO database (https://abortion-policies.srhr.org/) to determine the legality of abortion in each of the studied settings.

We were confident that this search was comprehensive and contained all relevant information that was needed to answer our realist review question. We subsequently re-screened this search for the purposes of the realist review as follows:

1. A subject expert (WN) screened all full texts to identify documents that were specifically related to medical abortion at home and then grouped these into the stages outlined in our intitial programme theory.
2. PB read these papers to identify whether they were relevant to developing our initial programme theory.

Papers were considered useful for theory development and testing where they offered empirical evidence that could refine, refute or confirm developing emerging realist programme theory and inform the development of context-mechanism-outcome (CMO) configurations within it. [28] (Appendix 2) This meant including all papers that were relevant to people's experience of each stage of the medical abortion at home journey and the support provided that might modify this

For peer review only - http://bmjopen.bmj.com/site/about/guidelines.xhtml

experience. We used a broad definition of 'support' to include any intervention by the individual having the abortion, the health care system or friends and family intended to improve or maintain health or wellbeing before, during and after the abortion process.

Realist review processes are iterative so as our CMO configurations developed we revisited the list of included papers for the scoping review to look for additional relevant materials. We also completed additional searches as required including handsearching reference lists and completing new searches, to seek out more relevant data.

Data extraction

The final selected papers regarding each stage of abortion care were read and re-read by PB and, for the post abortion contraception, by CF. Findings were summarised in spreadsheets which contained information on key relevant findings from each paper, grouped according to the programme theory. Based on their interpretations of these findings PB and CF developed CMO configurations (CMOCs) for each stage of the medical abortion process. No uniform data set was extracted from each paper, rather the data (verbatim sections of text) of each paper that were relevant to each emerging CMOCs were grouped together and iteratively used during the analysis process (see below) to develop CMO configurations.

Data analysis and synthesis

A realist logic of analysis uses data to produce causal explanations for outcomes that occur within a programme theory in the form of CMOCs. A CMOC is a proposition that explains what element of an initiative works, for whom and in what circumstances and is the primary way of reporting findings within a realist review. Within a CMOC, the causal claim being made is (in its simplest form), when a particular context is present, it 'triggers' or 'activates' a particular mechanism, which causes a particular outcome. Mechanisms are hidden causal processes that are context-sensitive and are usually inferred based on interpretations of the data. Data to inform our interpretation of the relationships between contexts, mechanisms and outcomes were sought within and across documents (e.g. mechanisms inferred from one document helped explain the way contexts influenced outcomes in a different document). Synthesising data from different documents is often necessary to compile CMOCs, since not all parts of the configurations will be articulated in every document. During our analysis, we used interpretive cross-case comparison to understand and explain how and why reported outcomes have occurred.

Refining programme theory

We iteratively refined our initial programme theory as the review progressed based on our interpretations of the data from the included papers. For each stage of the abortion at home process theory we sought to unpack what support is needed. Our final programme theory contains CMOCs that explain the outcomes achieved by the support provided, why it happens and in which contexts.

Patient and Public Involvement

The whole SACHA Study, including the realist review, was consulted by an Advisory Group with PPI members, who helped in developing the research questions and methodology. The development of

For peer review only - http://bmjopen.bmj.com/site/about/guidelines.xhtml

the research question and outcome measures were entirely informed by patients' priorities. Patient representatives were not involved in the conduct, analysis or interpretation of the results. The results of the study will be disseminated to the public and to patients who were involved in other components of the study through online newsletters, press releases, informational videos posted online and through other activities informed by the consultations with the Advisory Group.

For peer review only

For peer review only - http://bmjopen.bmj.com/site/about/guidelines.xhtml

Results

Our searches identified 27,982 potentially relevant abstracts for both reviews. After duplicates and papers with incorrect dates or excluded literature types were removed 12,401 titles and abstracts were screened with 944 selected for full text assessment. At this stage, 590 studies were excluded which did not meet the inclusion criteria or were duplicates. The remaining full texts were screened for potential relevance to the realist review question and 244 studies were categorised by a content expert (WN) against the inclusion criteria and the initial programme theory. Additional detailed screening of these 244 studies indicated that 35 contained data that were relevant to support theory building and testing and hence were included. A further 12 papers were identified as relevant during the analysis through hand searching and expert consultation. At the end of the analysis, updating the searches identified 43 new abstracts of which 3 were included giving a total of 50 included papers (Figure 2). A table summarising the included papers is given in Appendix 3.

Figure 2: PRISMA diagram

For peer review only

For peer review only - http://bmjopen.bmj.com/site/about/guidelines.xhtml

Our results are structured by our programme theory. For each stage, we present a narrative explanation of our findings followed by a table of the CMOCs that underpin the explanation. We found that the evidence on some of the stages in our programme theory were clustered and therefore the following stages are presented together:

- 'Understanding abortion options' and 'Choosing a method/setting'
- 'Accessing an abortion' and 'Pre-abortion counselling assessing gestational age'
- 'Taking mifepristone' and 'Taking misoprostol'.

Understanding abortion options and choosing a method

Choice of abortion methods and settings was influenced by service factors (number of appointments, timing and wait for appointments), personal responsibilities (caring/work commitments), geography (travel time/distance), relationships and need for privacy/secrecy and the extent to which people wished to be aware of/involved in/in control of the process. (Table 2) [12, 29-35] Those choosing medical abortion at home made this decision on the basis of their social responsibilities (such as the need to provide childcare) or when a home abortion enabled them to maintain their confidentiality, rather than their personal preferences about the procedure itself. [12, 21, 29, 35-37] Conversely, some people chose hospital care because it limits knowledge of the procedure among household members (for example, an abusive or unsupportive partner) and requires less responsibility for the process. [32, 34] Although we were not specifically seeking literature on reasons for surgical abortion, people cited a wish to be asleep/unaware of the procedure or to avoid pain/bleeding/sight of the products of conception ortaking the mifepristone [36, 38] or because it could be completed quickly. [39]

People valued frank descriptions from health professionals about the range of experience of medical abortion. [31, 39-43] A participant in one study commented that 'It's good to do it at home but I think a good level of honesty from the nurse is helpful because it's on the cusp of being copeable with'. [38] Fear about the abortion procedure was the most frequently cited barrier to accessing an abortion in one study, with information as the most highly ranked facilitator to care. [41] High levels of satisfaction were associated with information on abortion delivered through short films in clinical contexts. [43-45] It is notable that in this study the film was rated as more informative than a leaflet even though the content was the same. Developing high quality information resources is challenging because of the need to offer both clear clinical instructions and a wide range of experience that enables people to prepare themselves, their supporters and their surroundings. One study evaluating an animated account of one person's experience of medical abortion at home showed that this approach was well received but participants were concerned that their own experience was different from that shown. [43]

Table 2: CMOCs for 'understanding options' and 'choosing a method'

	CMOC
1	When people who want an abortion are offered a choice of procedures (c) they are more likely to be satisfied with their experience (i.e. predict choice of this method again) (o) because they feel their decision is their own (i.e. empowered in their choice) (m).[24, 29, 36, 37, 38, 40, 44, 45]
2	Where the information provided about each procedure option includes clinical safety and effectiveness and experiential information (c) people are in a better position to make an informed choice (o) because they are more likely to have the information needed to do so (m) [41, 46]

For peer review only - http://bmjopen.bmj.com/site/about/guidelines.xhtml

3	Where a range of personal experience is included that includes realistic accounts of pain/bleeding (c) then people are more likely to be satisfied with their choice (o) because they know what to expect and feel in control of the process (m) [31, 39-43]
4	When people have social responsibilities in their lives (c), they will choose the method for abortion that fits best with these responsibilities (o) because of convenience (m) [21, 29, 31, 48]
5	When people would prefer not to take an active role in the abortion process (c), they may choose a surgical abortion (o) because it enables them to distance themselves from the abortion process (m) [23, 31, 36, 47, 48]
6	When people have doubts about their abilities to carry out a medical abortion properly (c), they are more likely to request they are closely supervised (e.g. complete the abortion within a health care context) (o), because of the emotional and practical support provided (m) [36-38]
7	When people have had what they consider to be an unpleasant experience of medical abortion (c), they will be unlikely to choose medical abortion again (o), because they want to avoid the experience (m) [24, 38, 39, 37, 42]

Accessing a service/pre-abortion consultation/assessing gestational age

People value telephone/telemedicine self-referral options, [10, 12, 21], because of the privacy, autonomy and convenience they potentially afford and because they remove risk of judgmental attitudes from referring professionals (Table 3). People rated distance to clinic and waiting times as more important than whether counselling was via telemedicine or face to face [30] and reasons for preferring a telemedicine consultation included the ability to engage from a more comfortable space (at home) and feeling more able to ask questions. [49] However, some people need to take action to speak privately, for example, completing the consultation in their car. [49, 50]

The pre-abortion consultation is variable and may include an initial in-person consultation with/without an ultrasound scan and/or an examination or a fully remote service including self-reporting of gestational age. [22] Medical abortion without a prior ultrasound scan is safe [12] and in one qualitative study showed that some people preferred not to have an ultrasound scan because they associated it with having positive experiences of desired pregnancy [49] and in another study ultrasound was rated as unimportant for 53% of participants.

Table 3: CMOCs for 'Accessing a service/pre-abortion consultation

	CMOC
1	Where people can self-refer for abortion services via telephone or telemedicine services (c) they are more likely to be satisfied with this process (o) because they can avoid feeling judged by referring professionals (m).[22, 50-52]
2	When people who want privacy, convenience and rapid access to abortion are offered the initial consultation by telephone or telemedicine (c), they are more likely to be satisfied (o), because their remote consultations are often available more quickly and are less likely to disrupt existing responsibilities (m) [21, 29, 30, 48, 49]

Taking the abortion medications

Most of the literature focuses on the experience of taking misoprostol because this generates the most physical side effects of medical abortion; however, some studies suggest that people may find taking the mifepristone the most emotional part of the treatment regimen and that this should be acknowledged in the information and support provided.(Table 4) [40] Self-administering misoprostol enabled people to control the timing of their abortion and prepare by making a comfortable space or obtaining things that they might need such as analgesia or hot water bottles,

For peer review only - http://bmjopen.bmj.com/site/about/guidelines.xhtml

fit the abortion around other responsibilities or keep it private. [24] When given a choice of how to take the misoprostol, 70% in one study chose to self-administer it sublingually, rather than vaginally because they felt more confident that they had inserted it correctly using this approach. [21] Side effects from the misoprostol were common, with 77% of people experiencing one of the common symptoms (nausea, vomiting, diarrhoea or headaches). [21]

Table 4: CMOCs for 'Taking the abortion medication'

	CMOC
1	When people who want an abortion have reservations about their active participation in the process © they may prefer not to self-administer the medications (o) because they feel personally responsible for ending the pregnancy (m) [40]
2	When people are given the option of self-administering misoprostol (c) they are better able to prepare for the process (o) because they can choose the time and place of the medical abortion (m) [24]
3	When people lack confidence in their ability to self-administer misoprostol (c) they prefer that this be done by a health professional (o) because they can then be confident that the procedure is done correctly (m) [21, 24, 38]

Managing expulsion

Experience of managing the expulsion of the products of conception is highly variable (Table 5). People interviewed after medical abortion at home point to the importance of understanding 'what is normal' as an important factor to help manage anxiety and to guide seeking clinical advice. Remote clinical support is important and highly valued and is used by 10-50% of people who choose medical abortion at home. [24, 37, 53, 21]

While most people find the process acceptable, the evidence shows that some experience pain and bleeding that is unexpected or distressing and cite this as the reason for future preference for an alternative method. [24, 37, 38] In one study 94% of respondents had experienced pain with a median reported pain score of 6.7 (on a scale of 1-10) though 57% reported their pain as better or the same as they were expecting. [54] 93% took analgesia This is consistent with mean maximum pain scale of 6-8 reported elsewhere. [55] In one study half of people experienced more bleeding than they anticipated [37] and in another 77% of participants reported that it was more than their usual menstrual period. [54]

The pain relief offered to people completing medical abortion at home is not standard across services. When people completing a medical abortion at home were provided with a combination of paracetamol, ibuprofen and rectal diclofenac, almost all used all three medications, one quarter accessed additional analgesia and 65% used additional non-pharmacological measures such as heat and rest to reduce pain. [37] In recent UK studies, 93% used pain relief (41-92% paracetamol, 39-61% ibuprofen 64% dihydrocodeine/codein). [25, 54]

Most people who experienced significant pain and bleeding felt more comfortable coping with this at home, "…it is such a physical and emotional process so, y'know, at home's better. You basically need to make friends with your toilet for 8 hours, so at home's better. I was such a mess. I would be embarrassed for people to see me like that."[38]. However, a minority would have preferred to have in-person health professional care because of the practical and emotional support provided/ [37, 24, 23]

When people having medical abortion at home have had a previous pregnancy (ending in live birth, abortion or miscarriage), they are less likely to report pain that is distressing or to request professional support in comparison with those who have not had a previous pregnancy. However, they are no less likely to report heavy bleeding. [37] One reason to choose medical abortion at home is that people wish to be aware of the process of the abortion. Some find it helpful to know that the pregnancy has been passed if they see the products of conception, while others find it distressing. [38]

For peer review only - http://bmjopen.bmj.com/site/about/guidelines.xhtml
For peer review only

Some people having an abortion valued the support of partners, family and friends for company or practical support while others valued the opportunity to complete the abortion alone. [24] Partners (all men in cited study) interviewed about their experience of supporting people having a medical abortion expressed feelings of sadness, worry and concern, and sometimes ambivalence, about the decision to have an abortion as well as valuing the opportunity to support their partners through the process. [56]

Table 5: CMOCs for 'Managing expulsion'

	CMOC
1	Where people experience pain or bleeding that is outside the limits of what they were told to expect (c) they are more likely to request clinical support (o) because they are concerned that their health may be at risk (m) [23, 24, 37]
2	Where people having medical abortion at home have also had a previous pregnancy (live birth/abortion/miscarriage) (c), they are less likely to report pain that is distressing and to request professional support (o) because the experience is more familiar (m) [37, 50]
3	Where people receive adequate pain relief during medical abortion (c) they are more likely to be satisfied with the whole experience of medical abortion at home (o) as they were more comfortable (m)[24,37,38]
4	Where people want to have privacy when having an abortion (c), they may choose to manage the abortion process at home (o) because they feel less exposed (m) [35, 38]
5	Where people have family/friends/partners whom they know will be supportive when they have an abortion (c) then they value their presence during a medical abortion at home (o) because of the practical and emotional help that they provide (m). [24, 56]

Assessing the success of abortion

There is good evidence to suggest that people can self-assess the success of abortion [57-62] (Table 6). There was little evidence in the literature of people's thoughts and feelings about this process.

Table 6: CMOC for 'Assessing the success of abortion'

	CMOC
1	When people are made aware of the symptoms and signs that indicate that the abortion has been successful (c), they are able to make accurate assessments (o) because they know what to look for (m) [57-62]

Choosing contraception

For some people the time of abortion provision is a convenient, appropriate and valued opportunity to discuss contraception and national/international guidelines recommend this. [58] (Table 7) For those people opting for implants, having them inserted when medical abortion medication is provided, or for those choosing an intrauetine devices, an appointment booked shortly after medical abortion, means they are more likely to receive their contraceptive of choice. [38, 63, 64] However, medical abortion via telemedicine limits the opportunity to do this. For other people, a contraceptive discussion at the time of the medical abortion consultation is unwelcome [65] and may be associated with a sense of being judged and pressurised about contraception. [38] Some people also prefer to focus on managing their abortion before considering contraception. [38, 65-67] In this situation the offer of a delayed telephone or face to face contraception discussion can be welcome including for those who have had difficulty finding a contraceptive method that suits them. [38, 68-71]

Table 7: CMOCs for 'Choosing contraception'

For peer review only - http://bmjopen.bmj.com/site/about/guidelines.xhtml

	CMOC
1	At the time of abortion provision (c) some people are happy to discuss or be provided with contraception (o) because of the salience of this issue for them and the convenience (m) [38]
2	When people opting for long-acting reversible contraceptives (LARC) have an appointment booked for them when receiving abortion medication at a time and place of their choosing after the abortion, (c) they are more likely to receive their contraception of their choice (o) because of salience (m) and less work for users in making the appointment (m) [63, 64]
3	When people who are focused on the abortion procedure are offered contraceptive advice at a later date (c), they are more likely to welcome a discussion about contraception (o), because they have the capacity to consider this (m)[38, 68-71]
4	When people who have had a previous abortion are offered telephone contraceptive counselling following abortion (c) they are more likely to take this up (o) because they may want help with complex contraceptive needs (m) [68, 70, 71]
5	When contraception is raised at the time of abortion (c), people can feel judged (o) if health care providers imply that it is important to prevent future unintended pregnancies (m) [38]
C6	When blended (digital linked to face to face) post abortion support and contraception services are designed with user input (c) they are likely to be acceptable and accessible to users (o) because they address their needs (m). [72]

For peer review only

For peer review only - http://bmjopen.bmj.com/site/about/guidelines.xhtml

Discussion

Choice of abortion procedure remains essential

Increased expectations of self-care, access to digital health services and the COVID 19 pandemic may have reduced choice of abortion method. High levels of satisfaction are reported after medical abortion but variation in experience means that choice of method remains important. [21, 23, 31, 36] Though medical abortion at home is a lower cost procedure than medical abortion in hospital which, in turn, is a lower cost procedure than surgical abortion [73], personal preference, past experience or context may make some abortion processes, for some people, difficult, distressing or unsafe. [74, 75] People who may have lower levels of satisfaction with medical abortion at home include those who: have not had a previous pregnancy, do not have access to a comfortable and private space at home, e.g., those experiencing homelessness, do not have support from family/partners/friends, have inflexible personal or employment responsibilities, or wish to disengage from the process.

Realistic information on the range of experience of abortion at home is important so that the abortion does not generate surprise or anxiety and so that people can adequately prepare for it at home.

Informed decisions require information on the potential advantages and disadvantages of the available treatment options and abortion providers should offer information on the effectiveness, safety, health risks and benefits and the expected range of experiences of all abortion options. Although the effectiveness of abortion procedures is important to inform choice as up to 5% may require a surgical evacuation to complete the abortion [76-78], effectiveness was not mentioned in user accounts of reasons for choosing medical abortion at home. Our review shows that it is important to communicate: a range of experiences, the importance of context (for example, where there is lack of privacy) or personal experience (for example, those with previous pregnancies report less pain). There is limited recent research on the best strategies to communicate this information and further work is required to collate and test good practice.

Acknowledging the implications of moving a process from a health care setting to home

Guidelines on support for medical abortion at home may not sufficiently acknowledge the work that people do to prepare their space, manage their privacy, fulfil their work and caring obligations around the process, make decisions on pain relief, clean household areas, decide when and how to take misoprostol, assess their experience against what is normal and decide when to ask for professional help. There is a need to understand the new types of information/advice required to support people who do this work. The response may involve new technologies (e.g. medical abortion support at home apps), new or developing roles (e.g. medical abortion doulas) or new forms of peer support (e.g. discussion forums to help with preparation and share experience). Further research is required to understand and define health service responsibilities when procedures move from clinically facilitated to self-managed in general and for medical abortion at home in particular.

Understanding optimal pain relief

There is no consensus within the literature on analgesia for those who experience pain during medical abortion although a Cochrane Review is currently in progress on this topic. [79] There is currently significant variation in practice described in terms of the analgesics offered, the schedule and route of administration and whether these are provided by health services.

More choice within the procedure

Enabling people to tailor their abortion experience could be an empowering strategy within a process that is often experienced as disempowering. [32, 80] Options include when and how to take medications, pharmaceutical and other strategies for pain relief, the amount and form of support available and when contraception is discussed.

For peer review only - http://bmjopen.bmj.com/site/about/guidelines.xhtml

Contraception
Peri-abortion contraception counselling should be delivered without pressure to take up contraception as some people find this stigmatising. A choice of when and where to discuss contraception is important. The option of delayed counselling/provision is helpful for some and may be the only option for those who are having intrauterine devices or implants and choose telemedicine abortion.

Strengths and limitations
We were systematic and transparent in our approach to the realist review which was conducted in accordance with the RAMSES standards. [27] Our authorship team represents a variety of clinical and academic backgrounds, ensuring divergence in our analysis and we benefited from expert feedback from the multi-disciplinary SACHA team throughout the review. Limitations include our analysis on publicly accessible literature, located through recognised research databases and Google. We focused on the settings where abortion is legal to draw conclusions most relevant to the UK context, which meant our eligibility criteria screened out settings where abortion is not currently legal, which often included low- and middle-income country studies. There were clear gaps in the evidence that we found and we have highlighted these in our conclusions.

Further research needed
Based on our review of the literature we recommend that further research is needed on:

- Optimal strategies for providing information and supporting choice of procedure/setting.
- Understanding how medical abortion at home involves or impacts family and friends (beyond partners).
- Optimal medications for pain relief including the dosing, route of administration and length of time that pain relief is required.
- How to offer choice within the abortion procedure in a manner that is empowering and not overwhelming
- Optimal means for providing contraception advice /support after the abortion procedure.
- Perspectives and experiences of healthcare providers: as part of the SACHA Study, we are conducting a scoping review on healthcare practitioner preparedness to provide abortion globally, and a quantitative survey of practitioners in Britain, aimed at determining their experiences and attitudes towards abortion provision.

Recommendations
Our recommendations are summarized in our revised programme theory in figure 3 below.

- People should be given a choice of type and place of abortion
- Discussion of choice of method should include service factors, personal responsibilities, geographical context and preferred level of awareness of/engagement with the procedure
- Information to support choice of procedure should include the range of experience of medical abortion and realistic accounts of pain and bleeding.
- Health system support for the abortion process should acknowledge the new types of work that people take on when they manage this process at home, for example preparing a space for the abortion.
- There should be opportunities to personalize experience including when and where to take medications, who is present, where to pass products of conception.
- Support for taking mifepristone should acknowledge the emotional significance of this for some people.
- Adequate pain relief is an essential part of satisfaction with medical abortion, particularly for those who are pregnant for the first time. Pain relief should be offered as well as information on non-pharmacological strategies to manage pain.

For peer review only - http://bmjopen.bmj.com/site/about/guidelines.xhtml

- People should be offered a choice of where and when to discuss contraception including opportunities to book LARC appointments if this is their preference.

Conclusions

Acknowledging the work done by patients when moving an intervention from clinic to home is important. This includes preparing a space, managing privacy, managing work/caring obligations, deciding when/how to take medications, understanding what is normal, assessing experience and deciding when and how to ask for help. Strategies that reduce surprise or anxiety and enable preparation and a sense of control support the transition of this complex intervention outside healthcare environments. It is important that options for those who are unable to take on these additional responsibilities or who prefer not to do so are maintained including clinical facilities and appropriately trained clinicians who can provide surgical abortion and medical abortion within health care facilities.

Figure 3: Revised programme theory with key learning from this review

For peer review only

For peer review only - http://bmjopen.bmj.com/site/about/guidelines.xhtml

Contributorship

RF, KW, CF & PB conceptualised the study. GW was responsible for the methodology. RF, KW, CF, PB, GW, RM, MP, RS and ML wrote the protocol. ML registered the review in the Prospero system (ID CRD42021225307) and conducted the searches. All authors screened the papers. WN conducted preliminary data extraction. All authors participated in the data analysis. AI composed the tables. PB, CF, WN and GW wrote the first draft. All authors contributed to the final manuscript. This paper reports on data collected as part of the SACHA Study: Shaping Abortion for Change, funded by the National Institute of Health Research. (www.lshtm.ac.uk/sacha) Collaborators of the SACHA Study provided feedback on all stages of the study.

Funding statement

This paper is a part of the SACHA Study, funded by the National Institute of Health Research, award NIHR129529

Competing interests

None

Protocol

Protocol can be found in Propsero under ID CRD42021225307

Ethical approval

No ethical approval was required for review of evidence.

Data sharing

Data extracted from studies and used for analysis can be requested from the corresponding authors.

References

1. Abortion statistics, England and Wales, 2020. (https://www.gov.uk/government/statistics/abortion-statistics-for-england-and-wales-2020/abortion-statistics-england-and-wales-2020
2. Public Health Scotland termination of pregnancy statistics https://publichealthscotland.scot/publications/termination-of-pregnancy-statistics/termination-of-pregnancy-statistics-year-ending-december-2020/).
3. Altshuler AL, Whaley NS. The patient perspective: perceptions of the quality of the abortion experience. Curr Opin Obstet Gynecol. 2018 Dec;30(6):407-413. doi: 10.1097/GCO.0000000000000492. PMID: 30299320.
4. Biggs MA, Ralph L, Raifman S, Foster DG, Grossman D. Support for and interest in alternative models of medication abortion provision among a national probability sample of U.S. women. Contraception. 2019 Feb;99(2):118-124. doi: 10.1016/j.contraception.2018.10.007. Epub 2018 Nov 15. PMID: 30448203.
5. Darney BG, Powell B, Andersen K, et al. Quality of care and abortion: beyond safety [published online ahead of print, 2018 May 7]. *BMJ Sex Reprod Health*. 2018;44(3):159-160. doi:10.1136/bmjsrh-2018-200060
6. Grossman, D., Grindlay, K., Buchacker, T., Lane, K., & Blanchard, K. (2011). Effectiveness and acceptability of medical abortion provided through telemedicine.

For peer review only - http://bmjopen.bmj.com/site/about/guidelines.xhtml

Obstet Gynecol, *118*(2 Pt 1), 296-303. https://doi.org/https://dx.doi.org/10.1097/AOG.0b013e318224d110
7. Fok WK, Mark A. Abortion through telemedicine. Curr Opin Obstet Gynecol. 2018 Dec;30(6):394-399. doi: 10.1097/GCO.0000000000000498. PMID: 30399015.
8. Aiken, A., Gomperts, R., & Trussell, J. (2017). Experiences and characteristics of women seeking and completing at-home medical termination of pregnancy through online telemedicine in Ireland and Northern Ireland: a population-based analysis. *BJOG*, *124*(8), 1208-1215. https://doi.org/https://dx.doi.org/10.1111/1471-0528.14401
9. Aiken, A. R. A., Digol, I., Trussell, J., & Gomperts, R. (2017). Self reported outcomes and adverse events after medical abortion through online telemedicine: population based study in the Republic of Ireland and Northern Ireland. *BMJ*, *357*, j2011. https://doi.org/10.1136/bmj.j2011
10. Aiken, A. R. A., Broussard, K., Johnson, D. M., & Padron, E. (2018). Motivations and Experiences of People Seeking Medication Abortion Online in the United States. *Perspect Sex Reprod Health*, *50*(4), 157-163.
11. Aiken, A. R. A., Starling, J. E., Gomperts, R., Scott, J. G., & Aiken, C. E. (2021a). Demand for self-managed online telemedicine abortion in eight European countries during the COVID-19 pandemic: a regression discontinuity analysis. *BMJ Sex Reprod Health*, bmjsrh-2020-200880. https://doi.org/10.1136/bmjsrh-2020-200880
12. Aiken, A., Lohr, P. A., Lord, J., Ghosh, N., & Starling, J. (2021b). Effectiveness, safety and acceptability of no-test medical abortion (termination of pregnancy) provided via telemedicine: a national cohort study. *BJOG*, *128*(9), 1464-1474. https://doi.org/10.1111/1471-0528.16668
13. Chong, E., Shochet, T., Raymond, E., Platais, I., Anger, H. A., Raidoo, S., Soon, R., Grant, M. S., Haskell, S., Tocce, K., Baldwin, M. K., Boraas, C. M., Bednarek, P. H., Banks, J., Coplon, L., Thompson, F., Priegue, E., & Winikoff, B. (2021). Expansion of a direct-to-patient telemedicine abortion service in the United States and experience during the COVID-19 pandemic. *Contraception*, *104*(1), 43-48. https://doi.org/10.1016/j.contraception.2021.03.019
14. Gambir, K., Garnsey, C., Necastro, K. A., & Ngo, T. D. (2020). Effectiveness, safety and acceptability of medical abortion at home versus in the clinic: a systematic review and meta-analysis in response to COVID-19. *BMJ Glob Health*, *5*(12). https://doi.org/10.1136/bmjgh-2020-003934
15. Grossman, D., Baba, C. F., Kaller, S., Biggs, M. A., Raifman, S., Gurazada, T., Rafie, S., Averbach, S., Meckstroth, K. R., Micks, E. A., Berry, E., Raine-Bennett, T. R., & Creinin, M. D. (2021). Medication Abortion With Pharmacist Dispensing of Mifepristone. *Obstetrics & Gynecology*, *Latest Articles*(online first),
16. Raymond, E., Chong, E., Winikoff, B., Platais, I., Mary, M., Lotarevich, T., Castillo, P. W., Kaneshiro, B., Tschann, M., Fontanilla, T., Baldwin, M., Schnyer, A., Coplon, L., Mathieu, N., Bednarek, P., Keady, M., & Priegue, E. (2019). TelAbortion: evaluation of a direct to patient telemedicine abortion service in the United States. *Contraception*, *100*(3), 173-177. https://doi.org/https://dx.doi.org/10.1016/j.contraception.2019.05.013
17. Schummers L, Darling EK, Dunn S, McGrail K, Gayowsky A, Law MR, et al. Abortion Safety and Use with Normally Prescribed Mifepristone in Canada. New England Journal of Medicine. 2022;386(1):57-67.

For peer review only - http://bmjopen.bmj.com/site/about/guidelines.xhtml

18. Upadhyay, U. D., Desai, S., Zlidar, V., Weitz, T. A., Grossman, D., Anderson, P., & Taylor, D. (2015). Incidence of emergency department visits and complications after abortion. *Obstet Gynecol*, *125*(1), 175-183. https://doi.org/10.1097/AOG.0000000000000603
19. Upadhyay, U. D., Schroeder, R., & Roberts, S. C. M. (2020). Adoption of no-test and telehealth medication abortion care among independent abortion providers in response to COVID-19. *Contracept X*, *2*, 100049. https://doi.org/10.1016/j.conx.2020.100049
20. Upadhyay, U. D., Koenig, L. R., & Meckstroth, K. R. (2021). Safety and Efficacy of Telehealth Medication Abortions in the US During the COVID-19 Pandemic. *JAMA Network Open*, *4*(8), e2122320. https://doi.org/10.1001/jamanetworkopen.2021.22320
21. Reynolds-Wright JJ, Johnstone A, McCabe K, Evans E, Cameron S. Telemedicine medical abortion at home under 12 weeks' gestation: a prospective observational cohort study during the COVID-19 pandemic. BMJ Sex Reprod Health. 2021 Oct;47(4):246-251. doi: 10.1136/bmjsrh-2020-200976. Epub 2021 Feb 4. PMID: 33542062; PMCID: PMC7868129.
22. Gill R, Norman WV. Telemedicine and medical abortion: dispelling safety myths, with facts. Mhealth. 2018 Feb 1;4:3. doi: 10.21037/mhealth.2018.01.01. PMID: 29552565; PMCID: PMC5847856.
23. Hoggart L, Berer M. Making the case for supported self-managed medical abortion as an option for the future. *BMJ Sexual & Reproductive Health* Published Online First: 23 June 2021. doi: 10.1136/bmjsrh-2021-201181
24. Harden J, Ancian J, Cameron S*, et al*. Women's experiences of self-administration of misoprostol at home as part of early medical abortion: a qualitative evaluation. *BMJ Sexual & Reproductive Health* 2021;**47:**144-149.
25. Meurice ME, Whitehouse KC, Blaylock R, Chang JJ, Lohr PA. Client satisfaction and experience of telemedicine and home use of mifepristone and misoprostol for abortion up to 10 weeks' gestation at British Pregnancy Advisory Service: A cross-sectional evaluation. Contraception. 2021 Jul;104(1):61-66. doi: 10.1016/j.contraception.2021.04.027. Epub 2021 May 8. PMID: 33974918.
26. Pawson R. Evidence Based Policy; a realist perspective. Sage, London, 2006
27. Wong, G., Westhorp, G., Manzano, A. *et al.* RAMESES II reporting standards for realist evaluations. *BMC Med* **14,** 96 (2016). https://doi.org/10.1186/s12916-016-0643-1
28. Duddy C, Wong G, Gadsby EW, Krska J, Hibberd V. NHS Health Check programme: a protocol for a realist review. BMJ Open. 2021 Apr 14;11(4):e048937. doi: 10.1136/bmjopen-2021-048937. PMID: 33853809; PMCID: PMC8054100.
29. Fix L, Seymour JW, Sandhu MV*, et al* At-home telemedicine for medical abortion in Australia: a qualitative study of patient experiences and recommendations. *BMJ Sexual & Reproductive Health* 2020;**46:**172-176.
30. Endler, M, Lavelanet, A, Cleeve, A, Ganatra, B, Gomperts, R, Gemzell-Danielsson, K. Telemedicine for medical abortion: a systematic review. *BJOG* 2019; 126: 1094– 1102.
31. Heath J, Mitchell N, Fletcher J. A comparison of termination of pregnancy procedures: Patient choice, emotional impact and satisfaction with care. Sex Reprod

For peer review only - http://bmjopen.bmj.com/site/about/guidelines.xhtml

Healthc. 2019 Mar;19:42-49. doi: 10.1016/j.srhc.2018.12.002. Epub 2018 Dec 3. PMID: 30928134.
32. Killinger K, Günther S, Gomperts R, Atay H, Endler M. Why women choose abortion through telemedicine outside the formal health sector in Germany: a mixed-methods study. BMJ Sex Reprod Health. 2022 Jan;48(e1):e6-e12. doi: 10.1136/bmjsrh-2020-200789. Epub 2020 Nov 23. PMID: 33229399.
33. Wiebe E. and Sandhu K.Access to abortion: what women want from abortion services. Journal of obstetrics and gynaecology Canada, 2008; 30:327-331.
34. Hamoda H, Critchley HO, Paterson K, Guthrie K, Rodger M, Penney GC. The acceptability of home medical abortion to women in UK settings. BJOG. 2005 Jun;112(6):781-5. doi: 10.1111/j.1471-0528.2004.00538.x. PMID: 15924537.
35. Atay H, Perivier H, Gemzell-Danielsson K*, et al*. Why women choose at-home abortion via teleconsultation in France: drivers of telemedicine abortion during and beyond the COVID-19 pandemic. *BMJ Sexual & Reproductive Health* 2021;**47:**285-292.
36. Robson SC, Kelly T, Howel D, Deverill M, Hewison J, Lie ML, Stamp E, Armstrong N, May CR. Randomised preference trial of medical versus surgical termination of pregnancy less than 14 weeks' gestation (TOPS). Health Technol Assess. 2009 Nov;13(53):1-124, iii-iv. doi: 10.3310/hta13530. PMID: 19906334.
37. Hedqvist M, Brolin L, Tydén T, Larsson M. Women's experiences of having an early medical abortion at home. Sex Reprod Healthc. 2016 Oct;9:48-54. doi: 10.1016/j.srhc.2016.07.003. Epub 2016 Aug 2. PMID: 27634665.
38. Purcell, C, Cameron, S, Lawton, J, Glasier, A, Harden, J. Self-management of first trimester medical termination of pregnancy: a qualitative study of women's experiences. *BJOG* 2017; 124: 2001– 2008.
39. Levine K, Cameron ST. Women's preferences for method of abortion and management of miscarriage. J Fam Plann Reprod Health Care. 2009 Oct;35(4):233-5. doi: 10.1783/147118909789587394. PMID: 19849917.
40. Makenzius M, Tydén T, Darj E, Larsson M. Autonomy and dependence--experiences of home abortion, contraception and prevention. Scand J Caring Sci. 2013 Sep;27(3):569-79. doi: 10.1111/j.1471-6712.2012.01068.x. Epub 2012 Aug 22. PMID: 22913927.
41. Smith JL, Cameron S. Current barriers, facilitators and future improvements to advance quality of abortion care: views of women. BMJ Sex Reprod Health. 2019 Apr 26:bmjsrh-2018-200264. doi: 10.1136/bmjsrh-2018-200264. Epub ahead of print. PMID: 31028169.
42. Rafferty KA, Longbons T. *#AbortionChangesYou*: A Case Study to Understand the Communicative Tensions in Women's Medication Abortion Narratives. Health Commun. 2021 Nov;36(12):1485-1494. doi: 10.1080/10410236.2020.1770507. Epub 2020 Jun 1. PMID: 32482094.
43. Sherman S, Harden J, Cattanach D, Cameron ST. Providing experiential information on early medical abortion: a qualitative evaluation of an animated personal account, *Lara's Story*. J Fam Plann Reprod Health Care. 2017 Oct;43(4):269-273. doi: 10.1136/jfprhc-2016-101641. Epub 2017 Jul 8. PMID: 28689176.
44. Powell-Jackson R, Glasier A, Cameron ST. Benefits of using a digital video disk for providing information about abortion to women requesting termination of

For peer review only - http://bmjopen.bmj.com/site/about/guidelines.xhtml

pregnancy. Contraception. 2010 Jun;81(6):537-41. doi: 10.1016/j.contraception.2010.01.015. Epub 2010 Feb 19. PMID: 20472123.
45. Reynolds-Wright JJ, Belleuvre F, Daberius A, Faucher P, Gemzell-Danielsson K, Cameron ST. Information on early medical abortion for women using an audiovisual animation vs face-to-face consultation: A consortium randomized and quasi-randomized trial. Acta Obstet Gynecol Scand. 2020 Dec;99(12):1611-1617. doi: 10.1111/aogs.13944. Epub 2020 Jul 12. PMID: 32573767.
46. Norman WV, Hestrin B, Dueck R. Access to Complex Abortion Care Service and Planning Improved through a Toll-Free Telephone Resource Line. Obstet Gynecol Int. 2014;2014:913241. doi: 10.1155/2014/913241. Epub 2014 Feb 13. PMID: 24693291; PMCID: PMC3945945.
47. Whitehouse, K.C., Blaylock, R., Makleff, S. *et al. It's a small bit of advice, but actually on the day, made such a difference…*: perceptions of quality in abortion care in England and Wales. *Reprod Health* **18,** 221 (2021). https://doi.org/10.1186/s12978-021-01270-0
48. Rowlands, Sam, and Jeffrey Wale. "A Constructivist Vision of the First-Trimester Abortion Experience." *Health and human rights* vol. 22,1 (2020): 237-249.
49. Boydell, N, Reynolds-Wright, JJ, Cameron, ST, Harden, J. Women's experiences of a telemedicine abortion service (up to 12 weeks) implemented during the coronavirus (COVID-19) pandemic: a qualitative evaluation. *BJOG* 2021;https://doi.org/10.1111/1471-0528.16813 128: 1752– 1761.
50. Porter Erlank C, Lord J, Church K. Acceptability of no-test medical abortion provided via telemedicine during Covid-19: analysis of patient-reported outcomes. *BMJ Sexual & Reproductive Health* 2021;**47:**261-268.
51. Low ST, Chen ZE, Cameron S. Women's experiences of self-referral to an abortion service: qualitative study. *BMJ Sexual & Reproductive Health* 2021;**47:**37-42.
52. Ehrenreich, K., Marston, C. Spatial dimensions of telemedicine and abortion access: a qualitative study of women's experiences. *Reprod Health* **16,** 94 (2019). https://doi.org/10.1186/s12978-019-0759-9
53. Finch RE, McGeechan K, Johnstone A*, et al.* Impact of self-administration of misoprostol for early medical abortion: a prospective observational cohort study. *BMJ Sexual & Reproductive Health* 2019;**45:**296-301.
54. Reynolds-Wright JJ, Johnstone A, McCabe K*, et al.* Adherence to treatment and prevalence of side effects when medical abortion is delivered via telemedicine: a prospective observational cohort study during COVID-19. *BMJ Sexual & Reproductive Health* Published Online First: 28 October 2021. doi: 10.1136/bmjsrh-2021-201263
55. Goldman AR, Porsch L, Hintermeister A, Dragoman M. Transcutaneous Electrical Nerve Stimulation to Reduce Pain With Medication Abortion: A Randomized Controlled Trial. Obstet Gynecol. 2021 Jan 1;137(1):100-107. doi: 10.1097/AOG.0000000000004208. PMID: 33278292.
56. Kero A, Lalos A, Wulff M. Home abortion - experiences of male involvement. Eur J Contracept Reprod Health Care. 2010 Aug;15(4):264-70. doi: 10.3109/13625187.2010.485257. PMID: 20809674.
57. Schmidt-Hansen M, Cameron S, Lohr PA, Hasler E. Follow-up strategies to confirm the success of medical abortion of pregnancies up to 10 weeks' gestation: a systematic review with meta-analyses. Am J Obstet Gynecol. 2020 Jun;222(6):551-563.e13. doi: 10.1016/j.ajog.2019.11.1244. Epub 2019 Nov 9. PMID: 31715147

For peer review only - http://bmjopen.bmj.com/site/about/guidelines.xhtml

58. Eshre Capri Workshop Group. (2017). Induced abortion. *Hum Reprod*, *32*(6), 1160-1169. https://doi.org/10.1093/humrep/dex071
59. Lokeland, M., Iversen, O. E., Engeland, A., Okland, I., & Bjorge, L. (2014). Medical abortion with mifepristone and home administration of misoprostol up to 63 days' gestation. *Acta Obstet Gynecol Scand*, *93*(7), 647-653. https://doi.org/10.1111/aogs.12398
60. Oppegaard, K. S., Qvigstad, E., Fiala, C., Heikinheimo, O., Benson, L., & Gemzell-Danielsson, K. (2015). Clinical follow-up compared with self-assessment of outcome after medical abortion: a multicentre, non-inferiority, randomised, controlled trial. *Lancet*, *385*(9969), 698-704. https://doi.org/10.1016/S0140-6736(14)61054-0
61. Raymond, E. G., Shochet, T., & Bracken, H. (2018). Low-sensitivity urine pregnancy testing to assess medical abortion outcome: A systematic review. *Contraception*, *98*(1), 30-35. https://doi.org/10.1016/j.contraception.2018.03.013
62. Raymond, E. G., Tan, Y.-L., Grant, M., Benavides, E., Reis, M., Sacks, D. N., Hannum, C., Frapp, S., & Weaver, M. A. (2018). Self-assessment of medical abortion outcome using symptoms and home pregnancy testing. *Contraception*, *97*(4), 324-328. https://doi.org/https://dx.doi.org/10.1016/j.contraception.2017.12.004
63. Pohjoranta E, Mentula M, Suhonen SP*, et al.* Predicting poor compliance with follow-up and intrauterine contraception services after medical termination of pregnancy. *BMJ Sexual & Reproductive Health* 2018;**44:**278-285.
64. E. Pohjoranta, S. Suhonen, M. Gissler, P. Ikonen, M. Mentula and O. Heikinheimo. Early provision of intrauterine contraception as part of abortion care-5-year results of a randomised controlled trial. Human reproduction (Oxford, England) 2020 Vol. 35 Issue 4 Pages 796-804
65. Iyer A, Wallace R, Kindratt TB, Gimpel N. Preferences for contraceptive counseling and access among abortion patients at an independent clinic in Texas. Patient Educ Couns. 2021 Sep 9:S0738-3991(21)00619-4. doi: 10.1016/j.pec.2021.09.010. Epub ahead of print. PMID: 34538463.
66. Matulich M., Cansino C., Culwell K.R., and Creinin M. D. Understanding women's desires for contraceptive counseling at the time of first-trimester surgical abortion. Contraception 2014; 89 (1):36-41
67. Kavanagh M., Carlin E., Jones R. Patient's attitudes and experiences related to receiving contraception during abortion care. Contraception, 2011; 84 (6): 585-593
68. Lohr PA, Aiken ARA, Forsyth T, *et al.* Telephone or integrated contraception counselling before abortion: impact on method choice and receipt. *BMJ Sexual & Reproductive Health* 2018;**44:**114-121.
69. Smith, C., Ly, S., Uk, V. *et al.* Women's views and experiences of a mobile phone-based intervention to support post-abortion contraception in Cambodia. *Reprod Health* **14,** 72 (2017). https://doi.org/10.1186/s12978-017-0329-y
70. Smith, C., Ly, S., Uk, V. *et al.* Process evaluation of a mobile phone-based intervention to support post-abortion contraception in Cambodia. *Contracept Reprod Med* **2,** 16 (2017). https://doi.org/10.1186/s40834-017-0043-8
71. Loeber. O. Contraceptive counselling to prevent repeat unintended pregnancy: The abortion client's perspective. European Journal of Contraception and Reproductive Health Care 2016 Vol. 21 Issue SUPPL. 1 Pages 75
72. Gill R, Ogilvie G, Norman WV, Fitzsimmons B, Maher C, Renner R
Feasibility and Acceptability of a Mobile Technology Intervention to Support

For peer review only - http://bmjopen.bmj.com/site/about/guidelines.xhtml

Postabortion Care in British Columbia: Phase I. J Med Internet Res 2019;21(5):e13387

73. NHS reference costs 2019/20 V2 https://www.england.nhs.uk/national-cost-collection/#nccdata2. Accessed 15/02/2022
74. Wingo E, Ralph LJ, Kaller S, Biggs MA. Abortion method preference among people presenting for abortion care. Contraception. 2021 Apr;103(4):269-275. doi: 10.1016/j.contraception.2020.12.010. Epub 2020 Dec 26. PMID: 33373612.
75. Blaylock R, Makleff S, Whitehouse KC*, et al*. Client perspectives on choice of abortion method in England and Wales. *BMJ Sexual & Reproductive Health* Published Online First: 20 September 2021. doi: 10.1136/bmjsrh-2021-201242
76. Creinin, M. D., & Grossman, D. A. (2020). Medication Abortion Up to 70 Days of Gestation: ACOG Practice Bulletin, Number 225. *Obstetrics & Gynecology*, *136*(4), e31-e47. https://doi.org/10.1097/aog.0000000000004082
77. Royal College of Obstetricians and Gynaecologists. (2015). *Comprehensive Abortion Care* (Best Practice Paper No. 2, Issue. Royal College of Obstetricians & Gynaecologists. https://www.rcog.org.uk/en/guidelines-research-services/guidelines/bpp2/
78. Royal College of Obstetricians and Gynaecologists. (2019). *Clinical Guidelines for Early Medical Abortion at Home – England*. Royal College of Obstetricians & Gynaecologists. https://www.rcog.org.uk/en/guidelines-research-services/guidelines/early-medical-abortion-home-england/
79. Reynolds-Wright JJ, Woldetsadik MA, Morroni C, Cameron S. Pain management for medical abortion before 14 weeks' gestation. Cochrane Database of Systematic Reviews 2020, Issue 1. Art. No.: CD013525. DOI: 10.1002/14651858.CD013525
80. Ireland, S., Belton, S., & Doran, F. (2020). 'I didn't feel judged': exploring women's access to telemedicine abortion in rural Australia. Journal of primary health care, 12(1), 49-56. https://doi.org/https://dx.doi.org/10.1071/HC19050

For peer review only - http://bmjopen.bmj.com/site/about/guidelines.xhtml

For peer review only

For peer review only - http://bmjopen.bmj.com/site/about/guidelines.xhtml

Figure 1. Stages of the medical abortion process (initial theory of change)

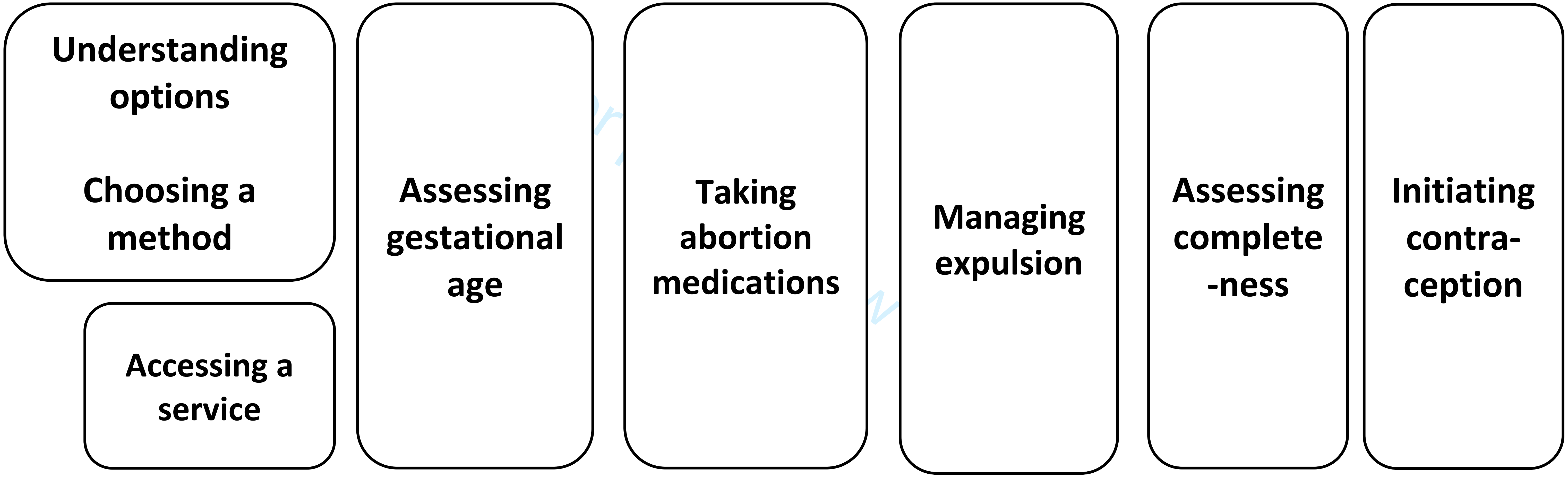

Figure 2. PRISMA 2009 Flow Diagram

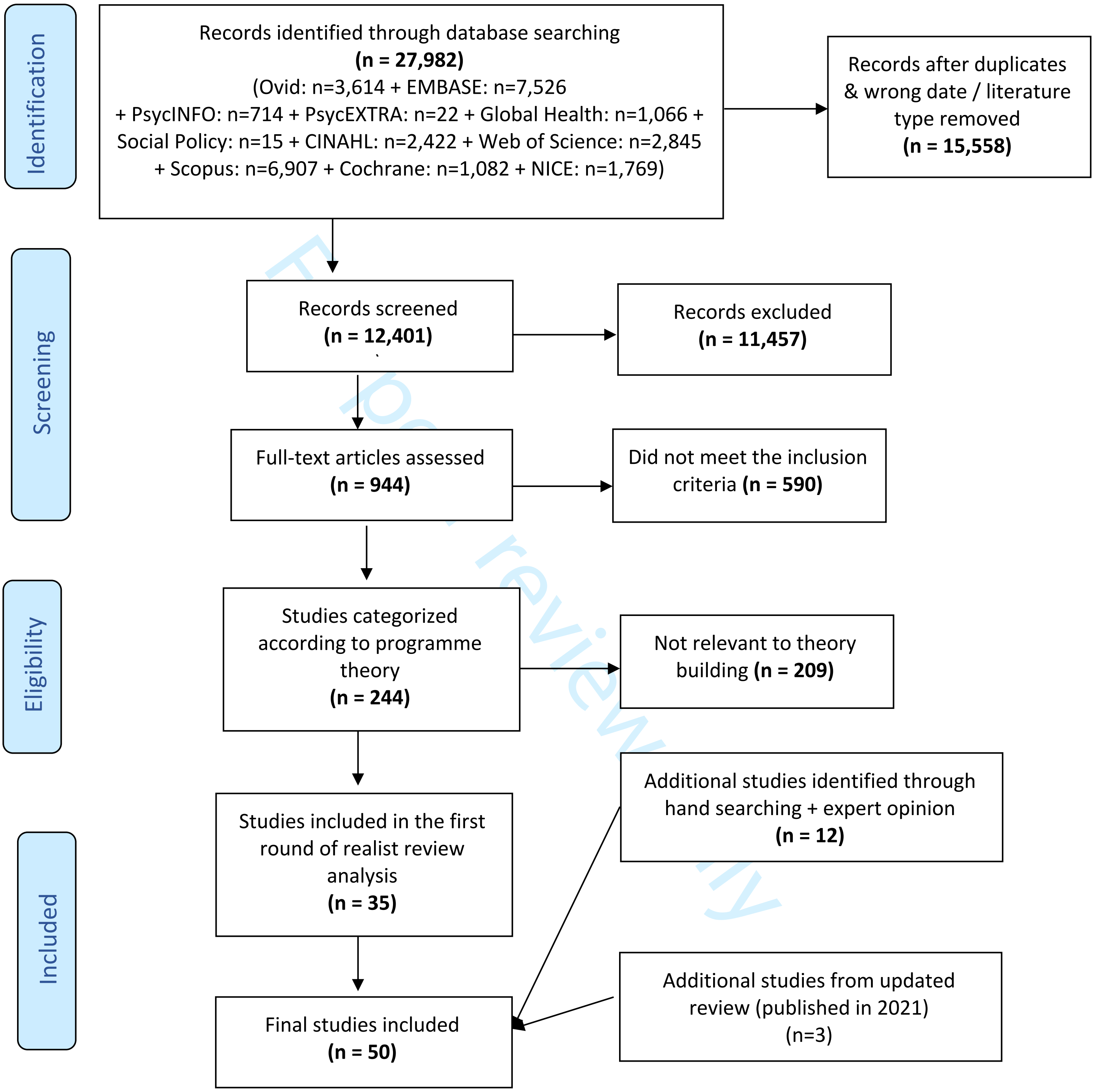

For peer review only - http://bmjopen.bmj.com/site/about/guidelines.xhtml

Figure 3. Revised programme theory with key learning from this review

Accessing a service + Pre-abortion counselling + Assessing gestational age

Offer:
- Choice of abortion method
- Information on range of experience
- Support to understand impact of the abortion on daily activities
- Support to consider who should be present/aware
- Help to practically/ emotionally prepare for the procedure

Understanding options + choosing a method

Offer:
- Self-referral for abortion via telephone / telemedicine / video call
- Choice when planning the procedure including pain relief

Taking abortion medications

Acknowledge that some people may have concerns about taking mifepristone

Follow user preference in terms of timing, route, and place of administration and who is present

Managing expulsion

Provide:
Analgesics and practical information on:
- Pain management & non-pharmaceutical pain relief
- How to manage bleeding
- How to manage expulsion including preparation for seeing and disposing of products of conception

Assessing complete-ness

Agree method of assessing completeness in advance and ensure the user has information and resources to be confident about the process

Choosing and accessing contraception

Offer a flexible, use-centred approach to contraceptive counselling, including choice of timing of contraceptive discussions

Be aware that the assumption that contraception is required may be experienced as judgmental or pressurising

For peer review only - http://bmjopen.bmj.com/site/about/guidelines.xhtml

Appendix 1: Databases searched and search terms

Ovid MEDLINE(R) and Epub Ahead of Print, In-Process & Other Non-Indexed Citations, Daily and Versions(R) 1946 to December 8, 2021; Embase Classic+Embase 1947 to 2021 December 08; APA PsycInfo 1806 to November Week 5 2021; APA PsycExtra 1908 to November 08, 2021; Global Health 1910 to 2021 Week 49; Social Policy and Practice 202010; CINAHL Plus, Web fo Science Core collection; Scopus (Elsevier); Cochrane Library.

Seach terms for Ovid: ((exp Abortion, Induced/) OR (abortion) OR (abortions) OR (pregnancy adj4 terminat*)) AND ((self care/) OR (self-management/) OR (Self Administration/) OR (Telemedicine/) OR (exp communications media/) OR (Internet-Based Intervention/) OR (Text Messaging/) OR (Outpatients/) OR (nurses/) OR (nursing staff/) OR (Nurse Midwives/) OR (Midwifery/) OR (Pharmacists/) OR (exp General Practice/) OR (Primary Health Care/) OR (Patient-Centred Care/) OR (self-manag*) OR (self-administer*) OR (self-induc*) OR (self-assess*) OR (self-sourc*) OR ("at home") OR ("home use") OR (home adj4 dosage) OR (home adj4 administ*) OR (home adj4 abortion) OR (telemedic*) OR (digit*) OR (online) OR (web) OR (website) OR (internet) OR (mobile) OR (sms) OR (text*) OR (messag*) OR (app) OR ("social media") OR (outpatient) OR (task-shar*) OR (task-shift*) OR (mid-level) OR (midlevel) OR (nurs*) OR (midwi*) OR (pharmacist) OR (pharmacy) OR (GP) OR (GPs) OR (general adj3 practic*) OR (non-speciali*) OR (non-clinical) OR (licen*) OR (qualif*) OR (decrim*) OR (woman-centered) OR (woman-centred) OR (women-centered) OR (women-centred) OR (patient-centered) OR (patient-centred) OR (gestation* adj5 limit*)) AND (("Patient Acceptance of Health Care"/) OR (Health Services Accessibility/) OR (exp patient satisfaction/ or patient preference/) OR (access*) OR (safe*) OR (accept*) OR (complicat*) OR (side effect*) OR (efficac*) OR (effect*) OR (feasib*) OR (prefer*) OR (satisf*) OR (feel*) or (autonom*) OR (delay*) OR (wait*) OR (experienc*) OR (uptake*) OR (time*) OR (distan*) OR (succe*) OR (impact) OR (opinion*) OR (best practice*) OR (capacity) OR (capab*) OR (competen*))

We searched Google (limited to the first 100 results) to identify grey literature and included known documents suggested by experts within the team and those identified through hand searches of the references of retrieved articles. Results from 01/01/2000 and 09/12/2022 that were peer reviewed research (journal articles and conference proceedings) theses or grey literature were included. We included papers where only a part of the investigation was relevant. We did not include books, book sections, cases, serials.

For peer review only

Appendix 2: Questions used to identify relevance

Five questions to operationalise a realist logic of analysis, adapted from Duddy et al (2021).

- Is there content that provides information on the context, mechanism or outcome of interventions? (CMO value)
- What do these data tell us about the context, mechanism or outcome of the interventions studies? (CMO outcomes)
- How should the CMO outcomes enable refinement of our initial programme theory? (refinement)

For peer review only

For peer review only

For peer review only - http://bmjopen.bmj.com/site/about/guidelines.xhtml

Appendix 3: Summary of papers included in the review

First Author	Date	Title	Source Type	Country	Setting	Participant Type	Number of Participants	Study Design	Is the paper relevant to the question?	Are the methods used appropriate?	Are the findings plausible?	Do conclusions support those of other studies?
Aiken, A. R. A	2018	Motivations and Experiences of People Seeking Medication Abortion Online in the United States	Original research paper	United States	Online	People seeking online medical abortion services	32	Qualitative interview study	Yes - paper explores motivations for considering self-managed abortion.	Yes - in-depth interviews were used to gather qualitative data on people's motivations and experiences of seeking online medical abortion.	Yes	Yes
Aiken, A.	2021b	Demand for self-managed online telemedicine abortion in eight European countries during the COVID-19 pandemic: a regression discontinuity analysis	Original research paper	Germany; Hungary; Italy; Malta; Netherlands; Northern Ireland; Portugal; Great Britain	Online	N/A	3915 requests for self-managed abortion to Women on Web (WoW)	Cross-sectional comparative analysis of requests for self-managed abortion pre- and post the Covid-19 pandemic	Yes - paper assesses whether the COVID-19 pandemic increased demand for self-managed medical abortion provided through online services and suggests reasons for trends in demand for online accessed abortion during the pandemic.	Yes - request rates in 8 countries are compared using regression discontinuity both before and after lockdown measures.	Yes	Yes
Atay	2021	Why women choose at-home abortion via teleconsultation in France: drivers of telemedicine abortion during and beyond the	Original research paper	France	Online	People accessing online medical abortion services	140	Mixed Methods (Cross-sectional survey of consultation data and email content analysis)	Highly - the paper explores drivers of at home, online supported, medical abortion during the pandemic and beyond.	Yes - the findings are based on appropriate analysis of consultations and emails extracted from an online medical abortion service to reveal the motivations and preferences of women seeking at home abortion.	Yes	Yes

For peer review only - http://bmjopen.bmj.com/site/about/guidelines.xhtml

		COVID-19 pandemic.										
Boydell	2021	Women's experiences of a telemedicine abortion service (up to 12 weeks) implemented during the coronavirus (COVID-19) pandemic: a qualitative evaluation	Original research paper	Scotland	Online	People accessing online medical abortion services	20	Qualitative interview study	Highly - paper explores women's experiences of accessing online medical abortion services.	Yes - the findings are based on appropriate use of qualitative interviews to explore women's experiences of telephone consultation; remote support; views on no pre-abortion ultrasound; and self-administration of abortion medications at home.	Yes	Yes
Ehrenreich	2019	Spatial dimensions of telemedicine and abortion access: a qualitative study of women's experiences	Original research paper	Utah, USA	Online	People accessing online medical abortion services	20	Qualitative interview study	Yes - the paper explores women's experiences of using online services for the first mandatory step when accessing medical abortion in Utah, an 'information visit'.	Yes - through in-depth interviews thoroughly explore experiences of using online services for information visits to fulfil abortion requirements.	Yes	Yes
Endler	2019	Telemedicine for medical abortion: a systematic review	Review	N/A	N/A	N/A	N/A	Systematic Review	Yes - the paper assesses the success rate, safety, and acceptability for women and providers of online medical abortion services.	Yes - a systematic review assessing a broad and significant number of relevant studies.	Yes	Yes
Erlank	2021	Acceptability of no-test medical abortion provided via telemedicine during Covid-19: analysis of patient-reported outcomes	Original research paper	United Kingdom	Online	People accessing online medical abortion services	1243	Qualitative survey study	Yes - the study reports patient-reported outcome measures assessing the quality of consultation, access to medicine after consultation, ability to manage the process at home and overall patient satisfaction.	Yes – early medical abortion patients invited to opt-in to a follow-up call post-procedure to answer clinical and satisfaction questions to measure acceptability of the service.	Yes	Yes

For peer review only - http://bmjopen.bmj.com/site/about/guidelines.xhtml

Eshre Capri Workshop Group	2017	Induced abortion	Workshop	N/A	N/A	N/A	N/A	N/A	Moderately - This paper presents commentary on induced abortion gathered at a workshop of experts in the field. The commentary included discussion of at home medical abortion with reference to online support.	N/A	N/A	Yes
Finch	2019	Impact of self-administration of misoprostol for early medical abortion: a prospective cohort study	Original research paper	Scotland	Home	People accessing abortion services both at home and at a facility	2430	Prospective observational study	Yes - the study explores the impact of Scotland's legalisation on home use of misoprostol for the purpose of early medical abortion on uptake and success rate, and on the provision of effective contraception on discharge.	Yes - prospective observational study followed the outcomes of two cohorts who received their abortion care, before and after the introduction of home use of misoprostol.	Yes	Yes
Fix	2020	At-home telemedicine for medical abortion in Australia: a qualitative study of patient experiences and recommendations	Original research paper	Australia	Online	People accessing online medical abortion services	24	Qualitative interview study	Highly - the study explores people's experiences obtaining a medical abortion through an online service and discusses people's reasons for choosing an online service.	Yes - in-depth interviews appropriately captures the experiences of people using the at-home telemedicine service.	Yes.	Yes
Gill	2019	Feasibility and Acceptability of a Mobile Technology Intervention to Support Postabortion Care in British	Original research paper	Canada	Abortion care facility	People attending an abortion care facility	58	Mixed-methods formative study (cross-sectional survey and semi-structured interviews)	Moderately - study aims to understand how people at surgical abortion clinics utilise their mobile phones to access health care information and their preferences for a mobile intervention	Yes - mixed-methods study appropriately explores a variety of aspects relevant to technology usage during abortion process.	Yes	Yes

For peer review only - http://bmjopen.bmj.com/site/about/guidelines.xhtml

BMJ Open

		Columbia: Phase 1							that supports follow-up care.			
Gill & Norman	2018	Telemedicine and medical abortion: dispelling safety myths, with facts	Editorial	N/A	N/A	N/A	N/A	Editorial commentary on recent research regarding online medical abortion services	Moderately - This paper presents commentary on recent research regarding online medical abortion services.	N/A	N/A	Yes
Goldman	2021	Transcutaneous Electrical Nerve Stimulation to Reduce Pain With Medication Abortion: A Randomized Controlled Trial	Original research paper	N/A	Abortion care facility	People undergoing medical abortion.	251	Randomised controlled trial (RCT)	Yes - evaluates whether high-frequency transcutaneous electrical nerve stimulation reduces experiences of pain during medical abortion.	Yes – randomized control trial shows that participants who received high-frequency transcutaneous electrical nerve stimulation after medical abortion had significantly lower posttreatment pain scored compared to those that received a sham procedure.	Yes	Yes
Hamoda	2005	The acceptability of home medical abortion to women in UK settings	Original research paper	England and Scotland	Home	People seeking hospital based abortion care	553	Qualitative survey study	Highly - the study explores the acceptability of home medical abortion to people UK settings including managing pain and bleeding at home.	Yes - self-complete questionnaires adequately measure people's views on the acceptability of medical abortion at home.	Yes	Yes
Harden	2021	Women's experiences of self-administration of misoprostol at home as part of early medical abortion: a qualitative evaluation	Original research paper	Scotland	Home	People that had recently undergone early medical abortion	20	Qualitative interview study	Highly - the study explores the experiences of people who accessed at home medical abortion and reported benefits of at home administration including flexibility, privacy and comfort.	Yes - qualitative data is collected exploring what people desire and valued during the process.	Yes	Yes
Heath	2019	A comparison of termination of pregnancy procedures: Patient choice,	Original research paper	Sweden	Abortion care facility	People requesting an abortion		A mixed-method prospective comparative study	Yes	Yes	Yes	Yes

For peer review only

		emotional impact and satisfaction with care										
Hedqvist	2016	Women's experiences of having an early medical abortion at home	Original research paper	Sweden	Home	People accessing medical abortion at home	119	Cross-sectional (semi-structured telephone interviews)	Highly - paper investigates people's experiences of having a medical abortion at home including experiences of pain and bleeding.	Yes - descriptive and comparative design enable an in-depth and balanced conclusion. Study also investigates differences between groups of people.	Yes	Yes
Hoggart and Berer	2021	Making the case for supported self-managed medical abortion as an option for the future	Editorial	N/A	Online	N/A	N/A	Editorial commentary on recent research regarding online medical abortion services	Moderately – this paper reviews recent research on online medical abortion services highlighting benefits and pointing out need for further development of services, suggesting a need for access to 24 hours support.	N/A	N/A	Yes
Iyer	2021	Preferences for contraceptive counselling and access among abortion patients at an independent clinic in Texas	Original research paper	USA	Abortion care facility	People attending an abortion care facility	181	Cross-sectional survey study	Yes - study explores preferences for contraceptive counselling and access to contraception.	Yes - self-administered surveys used as an appropriate method considering legally restrictive setting and associated stigma.	Yes	Yes
Kavanagh	2011	Patients' attitudes and experiences related to receiving contraception during abortion care	Original research paper	USA	Abortion care facility	People accessing abortion at an abortion care facility	542	Cross-sectional survey study	Yes - study documents attitudes towards contraceptive services and identifies participants characteristics associated with desire for contraception and interest in LARC.	Yes - appropriate methods for exploring attitudes and characteristics associated with specific needs relevant to contraceptive use.	Yes	Yes
Kero	2010	Home abortion - experiences of	Original research paper	Sweden	Home	Couples - where the male partner had been	23 couples	Qualitative interview study	Moderately - the paper explores the male partner's experience of being	Yes - qualitative method ensures high detailed analysis in experiences of	Yes	Yes

For peer review only - http://bmjopen.bmj.com/site/about/guidelines.xhtml

		male involvement				present when their female partner had had an at home medical abortion			present and supporting their partner during an induced home abortion.	both the pregnant woman and her partner.		
Killinger	2022	Why women choose abortion through telemedicine outside the formal health sector in Germany: a mixed-methods study	Original research paper	Germany	Online	People accessing an online medical abortion service	1090	Cross-sectional study analysing data from online consultations	Yes - the paper aims to understand the motivations and barriers to access for people who choose online abortion services outside of the formal health sector.	Yes - a cross-sectional study of data contained in online consultations and a content analysis of over 100 email texts.	Yes	Yes
Levine and Cameron	2009	Women's preferences for method of abortion and management of miscarriage	Original research paper	United Kingdom	Abortion care facility	People undergoing medical abortion, surgical abortion and surgical management of miscarriage	148	Cross-sectional survey study	Yes - the study explores views on medical treatment at home, and surgery under local anaesthesia, to determine whether new services should be developed.	Moderately - small sample size of self-administered anonymous questionnaires.	Yes	Yes
Loeber	2016	Contraceptive counselling for women with multiple unintended pregnancies: the abortion client's perspective	Original research paper	Netherland s	Abortion care facility	Survey – People attending an abortion care facility<br><br>Interviews – People attending an abortion care facility that had had more than three unintended pregnancies	212	Mixed methods (quantitative survey and semi-structured interviews)	Yes - study explores views on contraception use after induced abortion and post-abortion contraceptive counselling	Yes - mixed method approach appropriately explores experiences of post-abortion contraception and contraception counselling both in-depth and across a larger sample.	Yes	Yes

For peer review only - http://bmjopen.bmj.com/site/about/guidelines.xhtml

Lohr	2018	Telephone or integrated contraception counselling before abortion: impact on method choice and receipt	Original research paper	United Kingdom	Abortion care facility	People who had accessed abortion at an abortion care facility	18,573	Cross-sectional study of de-identified data	Yes - paper compares the characteristics of people who chose contraception counselling either over the telephone and separate from abortion consultation or face-to-face and integrated into consultation.	Yes - very large sample size which thoroughly explores demographic characteristics and contraceptive method choice.	Yes	Yes
Lokeland	2014	Medical abortion with mifepristone and home administration of misprostol up to 63 days' gestation	Original research paper	Norway	Abortion care facility	People seeking an at home medical abortion before 63 days'	1,018	Observational study	Yes - paper evaluates the acceptability of medical abortion at home, including pain and bleeding.	Yes – a large sample size of people experiencing at home abortion with follow up telephone recorded to asses bleeding, pain and acceptability.	Yes	Yes
Low	2021	Women's experiences of self-referral to an abortion service: qualitative study	Original research paper	Scotland	Abortion care service	People attending an abortion care service	21	Qualitative interview study	Yes - this study evaluates a self-referral service to abortion services by investigating its impact on people's experiences of the referral process.	Yes - semi-structured interviews to focus on people's experiences of the referral process.	Yes	Yes
Makenzius	2012	Autonomy and dependence - experiences of home abortion, contraception and prevention	Original research paper	Sweden	Home	People who have experienced an at-home abortion	37	Qualitative interview study	Yes - study explores people's experiences and needs related to care in the context of a home abortion.	Yes – interviews capture patients at-home abortion experiences and needs which fall under themes of autonomy (choice of at-home abortion, increase of privacy and control) and dependence (desire to be treated with empathy by health care workers and receive adequate information).	Yes	Yes
Matulich	2014	Understanding women's desires for contraceptive counselling at the time of	Original research paper	USA	Abortion care facility	People receiving surgical abortion at an abortion care facility	199	Qualitative survey study	Yes - paper investigates whether or not people presenting for a first-trimester surgical abortion want to	Yes - survey obtained demographic information and inquired about desire for contraceptive counselling.	Yes	Yes

For peer review only - http://bmjopen.bmj.com/site/about/guidelines.xhtml

		first-trimester surgical abortion							discuss contraception on the day of their procedure.			
Norman	2014	Access to Complex Abortion Care Service and Planning Improved through a Toll-Free Telephone Resource Line	Original research paper	Canada	Abortion care facility	N/A	N/A	Review of service provision model	Yes - study presents a review of a service provision model which has provided improved access to abortion care through a toll-free telephone abortion access and counselling service.	Yes - a descriptive analysis appropriately reports on the development and delivery of this service.	Yes	Yes
Oppegaard	2015	Clinical follow-up compared with self-assessment of outcome after medical abortion: a multicentre, non-inferiority, randomised, controlled trial	Original research paper	Austria; Finland; Norway; Sweden	Abortion care services	People requesting medical abortion	924	Randomised, controlled, non-inferiority trial	Yes - study compares clinical assessment with self-assessment of abortion outcome.	Yes - assigned people in a 1:1 ratio to attend routine clinical follow-up or to self-assess outcome at home with a semi-quantitative urine human chorionic gonadotropin test 1-3 weeks after abortion.	Yes	Yes
Pohjoranta	2018	Predicting poor compliance with follow-up and intrauterine contraception services after medical termination of pregnancy	Original research paper	Finland	Abortion care facility	People undergoing a medical abortion	605	RCT	Moderately - paper assesses factors associated with non-compliance with post-abortion services and evaluates differences in rates of attendance and intrauterine device insertion.	Yes – the study compared the intervention group booked to have IUD insertion 1-4 weeks after medical abortion and women in control group were advised to contact their PHC for follow-up and IUD insertion.	Yes	Yes
Pohjoranta	2020	Early provision of intrauterine contraception as part of abortion care - 5-year results of a randomised controlled trial	Original research paper	Finland	Abortion care facility	People undergoing an induced abortion	748	RCT	Moderately - paper investigates whether the incidence of subsequent termination of pregnancy can be reduced by providing IUD as part of the abortion service.	Yes – RCT assesses the effectiveness of early comprehensive provision of IUD after induced abortion. Intervention group provided with an IUD during surgical abortion and women in control group advised to	Yes	Yes

For peer review only - http://bmjopen.bmj.com/site/about/guidelines.xhtml

										contact primary healthcare for follow-up and IUD insertion.		
Powell-Jackson	2010	Benefits of using a digital video disk for providing information about abortion to women requesting termination of pregnancy	Original research paper	Scotland	Abortion care facility	People attending an abortion care facility and facility staff	226	Comparative cross-sectional survey design	Yes - paper examines the benefits of using a DVD for providing information about abortion.	Yes - questionnaires are used to appropriately measure people's satisfaction with the information received through the DVD and what more/less they would want it to include.	Yes	Yes
Purcell	2017	Self-managment of first trimester medical termination of pregnancy: a qualitative study of women's experiences	Original research paper	Scotland	Abortion care facility	People who are administered a medical abortion at a facility and return home to complete termination	44	Qualitative interview study	Yes - explores people's experiences of returning home to complete medical abortion, including self-monitoring of treatment process.	Yes - in-depth interviews to assess people's experiences in detail.	Yes	Yes
Rafferty & Longbons	2021	#AbortionChangesYou: A Case Study to Understand the Communicative Tensions in Women's Medication Abortion Narratives	Original research paper	United States	Online	People who have had a medical abortion	98 blog posts	Case study	Yes - study analyses people's narratives after having had a medication abortion though blogs posted on a website - Abortion Changes You.	Yes - using a case study approach enabled the study to explore multiple perspectives rooted in specific contexts.	Yes	Yes
Raymond	2018	Low-sensitivity urine pregnancy testing to assess medical abortion outcome: A systematic review	Review	N/A	N/A	N/A	N/A	Systematic review	Yes - the study explores data on the accuracy and acceptability of a strategy for identifying ongoing pregnancy after medical abortion treatment using a low-sensitivity pregnancy test (LSPT).	Moderately - A more comprehensive search across other databases could strengthen study.	Yes	Yes

For peer review only - http://bmjopen.bmj.com/site/about/guidelines.xhtml

Raymond	2017	Self-assessment of medical abortion outcome using symptoms and home pregnancy testing	Original research paper	United States	Abortion care facility	People undergoing medical abortion	343	Intervention study	Highly - study evaluates compliance with a strategy to enable medical abortion patients to assess treatment outcome on their own and decide whether to seek clinical follow-up.	Yes - appropriate methodology to clinically assess women's compliance to using a pregnancy test after medical abortion and women's ability to self-assess treatment outcome.	Yes	Yes
Reynolds-Wright	2020	Pain management for medical abortion before 14 weeks' gestation	Review	N/A	N/A	N/A	N/A	Systematic review	Yes - paper reviews pain relief regimens for the management of medical abortion.	Yes - wide variety of databases, conference abstracts and organisations systematically searched and analysed.	Yes	No
Reynolds-Wright	2021	Telemedicine medical abortion at home under 12 weeks' gestation: a prospective observational cohort study during the COVID-19 pandemic	Original research paper	Scotland	Online/Home	People choosing online medical abortion services	663	Prospective cohort study	Yes - the paper determines the efficacy of an online at-home medical abortion service, noting any complications after treatment and acceptability of care.	Yes - large sample size. Questionnaires and hospital database analyses allows for in-depth review of efficacy, complications and acceptability.	Yes	Yes
Robson	2009	Randomised preference trial of medical versus surgical termination of pregnancy less than 14 weeks' gestation (TOPS)	Original research paper	United Kingdom	Abortion care facility	People undergoing induced abortion and people attending a contraceptive and sexual health clinic	1,877	Randomised preference trial and economic evaluation with qualitative sub study	Yes - the paper explores the acceptability, efficacy and costs of medical abortion compared with surgical abortion and aims to understand people's decision-making processes and experiences.	Yes – participants are either randomly assigned a procedure type (with consent to random allocation) or choose their procedure type to evaluate whether in people without prior preference, acceptability of medical and surgical abortion is the same. A qualitative sub study explores people's preferences and decision making.	Yes	Moderately
Rowlands & Wale	2020	A Constructivist	Constructivist Vision	N/A	N/A	N/A	N/A	Constructivist vision	Moderately - This paper provides	N/A	N/A	Yes

For peer review only - http://bmjopen.bmj.com/site/about/guidelines.xhtml

		Vision of the First-Trimester Abortion Experience						commentary on how a high-quality abortion experience might be achieved in the first trimester if regulatory laws were dismissed.	commentary on recent research on induced abortion in the first trimester within the framework of a future vision of state obligation to provide high quality abortion care. This paper also provides an evidence-based set of ingredients that facilitate a positive high-quality abortion experience.			
Schmidt-Hansen	2020	Follow-up strategies to confirm the success of medical abortion of pregnancies up to 10 weeks' gestation: a systematic review with meta-analyses	Review	N/A	N/A	N/A	N/A	Systematic review	Yes - the review compares the effectiveness, safety, and acceptability of in-clinic and remote/self-assessment for confirming the success of medical abortion.	Yes - systematic review including RCT's from 2000 onward to ensure a broad range of studies captured. Quality of evidence compromised by small event rates, lack of blinding, and high attrition rates.	Yes	Yes
Sherman	2017	Providing experiential information on early medical abortion: a qualitative evaluation of an animated personal account, *Lara's Story*	Original research paper	Scotland	Abortion care facility	People who have had a medical abortion	13	Qualitative interview study	Yes - the study evaluates the views on an animated film, and its potential usefulness in providing information on medical abortion.	Yes - the use of interviews allows people to report their full experience and opinion in detail, also providing feedback or criticism.	Yes	Yes
Smith	2019	Current barriers, facilitators and future improvements to advance	Original research paper	Edinburgh, Scotland	Abortion care facility	People seeking induced abortion	154	Cross-sectional survey study	Yes - the study explores current barriers access to care, and what future improvements should	Yes - self-administered anonymous questionnaires to capture experiences.	Yes	Yes

For peer review only - http://bmjopen.bmj.com/site/about/guidelines.xhtml

		quality of abortion care: views of women							be implemented to abortion care services			
Smith	2017a	Women's views and experiences of a mobile phone-based intervention to support post-abortion contraception in Cambodia	Original research paper	Cambodia	Abortion care facility	People who had received a mobile phone based post-abortion contraception counselling service.	15	Qualitative interview study	Yes - the study assesses views and experiences of receiving the MObile Technology for Improved Family Planning (MOTIF) intervention, which aims to increase post-abortion contraception.	Yes - semi-structured interviews to capture in-depth data. Small sample size may be a limitation.	Yes	Yes
Smith	2017b	Process evaluation of a mobile phone-based intervention to support post-abortion contraception in Cambodia	Original research paper	Cambodia	Abortion care facility	People who had received a mobile phone based post-abortion contraception counselling service.	249	Process evaluation	Yes - the study assesses participants' interaction with the MOTIF intervention.	Yes - thorough process evaluation to assess associations with the intervention and post-abortion contraception use.	Yes	Yes
Upadhyay, U. D.	2021	Safety and Efficacy of Telehealth Medication Abortions in the US During the COVID-19 Pandemic	Original research paper	USA	Online	People accessing online medical abortion	141	Retrospective cohort study	Yes - the study assesses the safety and efficacy outcomes of a online medical abortion model.	Yes – appropriate methodology for assessing an online medical abortion service however the small sample size with some loss to follow-up, and thus some adverse events and ongoing pregnancies may have been undetected.	Yes	Yes
Whitehouse	2021	*It's a small bit of advice, but actually on the day, made such a difference...*: perceptions of quality in abortion care in England and Wales	Original research paper	England; Wales	Abortion care facility	People who have had an abortion at a facility withing the last 6 months	24	Qualitative interview study	Yes - the paper explores participants' experiences and views on abortion quality of care.	Yes - interviews are an appropriate methodology to assess experiences.	Yes	Yes

For peer review only - http://bmjopen.bmj.com/site/about/guidelines.xhtml

Wieβe	2008	Access to Abortion: What Women Want From Abortion Services	Original research paper	Canada	Abortion care facility	People seeking an abortion	441	Qualitative survey and interview study	Moderately - the paper identifies barriers to access for women seeking induced abortion at a facility.	Yes - questionnaire accurately explores demographic information, perceived barriers to access, and what women desire from abortion services. Interviews captured in-depth analysis to access questions.	Yes	Yes

For peer review only

For peer review only - http://bmjopen.bmj.com/site/about/guidelines.xhtml

Reporting checklist for systematic review (with or without a meta-analysis).

Based on the PRISMA guidelines.

Instructions to authors

Complete this checklist by entering the page numbers from your manuscript where readers will find each of the items listed below.

Your article may not currently address all the items on the checklist. Please modify your text to include the missing information. If you are certain that an item does not apply, please write "n/a" and provide a short explanation.

Upload your completed checklist as an extra file when you submit to a journal.

In your methods section, say that you used the PRISMAreporting guidelines, and cite them as:

Page MJ, McKenzie JE, Bossuyt PM, Boutron I, Hoffmann TC, Mulrow CD, Shamseer L, Tetzlaff JM, Akl EA, Brennan SE, Chou R, Glanville J, Grimshaw JM, Hróbjartsson A, Lalu MM, Li T, Loder EW, Mayo-Wilson E, McDonald S, McGuinness LA, Stewart LA, Thomas J, Tricco AC, Welch VA, Whiting P, Moher D. The PRISMA 2020 statement: An updated guideline for reporting systematic reviews

		Reporting Item	Page Number
**Title**			
Title	#1	Identify the report as a systematic review	1
**Abstract**			
Abstract	#2	Report an abstract addressing each item in the PRISMA 2020 for Abstracts checklist	2
**Introduction**			
Background/rationale	#3	Describe the rationale for the review in the context of existing knowledge	3
Objectives	#4	Provide an explicit statement of the objective(s) or question(s) the review addresses	3
**Methods**			

For peer review only

Eligibility criteria	#5	Specify the inclusion and exclusion criteria for the review and how studies were grouped for the syntheses	5-6
Information sources	#6	Specify all databases, registers, websites, organisations, reference lists, and other sources searched or consulted to identify studies. Specify the date when each source was last searched or consulted	Appendix 1
Search strategy	#7	Present the full search strategies for all databases, registers, and websites, including any filters and limits used	Appendix 1
Selection process	#8	Specify the methods used to decide whether a study met the inclusion criteria of the review, including how many reviewers screened each record and each report retrieved, whether they worked independently, and, if applicable, details of automation tools used in the process	6
Data collection process	#9	Specify the methods used to collect data from reports, including how many reviewers collected data from each report, whether they worked independently, any processes for obtaining or confirming data from study investigators, and, if applicable, details of automation tools used in the process	6-7
Data items	#10a	List and define all outcomes for which data were sought. Specify whether all results that were compatible with each outcome domain in each study were sought (for example, for all measures, time points, analyses), and, if not, the methods used to decide which results to collect	7
Study risk of bias assessment	#11	Specify the methods used to assess risk of bias in the included studies, including details of the tool(s) used, how many reviewers assessed each study and whether they worked independently, and, if applicable, details of automation tools used in the process	N/a
Effect measures	#12	Specify for each outcome the effect measure(s) (such as risk ratio, mean difference) used in the synthesis or presentation of results	7
Synthesis methods	#13a	Describe the processes used to decide which studies were eligible for each synthesis (such as tabulating the study intervention characteristics and comparing against the planned groups for each synthesis (item #5))	7
Synthesis methods	#13b	Describe any methods required to prepare the data for presentation or synthesis, such as handling of missing summary statistics or	7

For peer review only

		data conversions	
Synthesis methods	#13c	Describe any methods used to tabulate or visually display results of individual studies and syntheses	7
Synthesis methods	#13d	Describe any methods used to synthesise results and provide a rationale for the choice(s). If meta-analysis was performed, describe the model(s), method(s) to identify the presence and extent of statistical heterogeneity, and software package(s) used	7
Synthesis methods	#13e	Describe any methods used to explore possible causes of heterogeneity among study results (such as subgroup analysis, meta-regression)	7
Synthesis methods	#13f	Describe any sensitivity analyses conducted to assess robustness of the synthesised results	7
Reporting bias assessment	#14	Describe any methods used to assess risk of bias due to missing results in a synthesis (arising from reporting biases)	n/a
Certainty assessment	#15	Describe any methods used to assess certainty (or confidence) in the body of evidence for an outcome	7
Data items	#10b	List and define all other variables for which data were sought (such as participant and intervention characteristics, funding sources). Describe any assumptions made about any missing or unclear information	7
**Results**			
Study selection	#16a	Describe the results of the search and selection process, from the number of records identified in the search to the number of studies included in the review, ideally using a flow diagram (http://www.prisma-statement.org/PRISMAStatement/FlowDiagram)	8-9
Study selection	#16b	Cite studies that might appear to meet the inclusion criteria, but which were excluded, and explain why they were excluded	10
Study characteristics	#17	Cite each included study and present its characteristics	Appendix 3
Risk of bias in studies	#18	Present assessments of risk of bias for each included study	N/a
Results of individual	#19	For all outcomes, present for each study (a) summary statistics for	Appendix

For peer review only

studies		each group (where appropriate) and (b) an effect estimate and its precision (such as confidence/credible interval), ideally using structured tables or plots	3
Results of syntheses	#20a	For each synthesis, briefly summarise the characteristics and risk of bias among contributing studies	10-14
Results of syntheses	#20b	Present results of all statistical syntheses conducted. If meta-analysis was done, present for each the summary estimate and its precision (such as confidence/credible interval) and measures of statistical heterogeneity. If comparing groups, describe the direction of the effect	N/a
Results of syntheses	#20c	Present results of all investigations of possible causes of heterogeneity among study results	10-14
Results of syntheses	#20d	Present results of all sensitivity analyses conducted to assess the robustness of the synthesised results	n/a
Risk of reporting biases in syntheses	#21	Present assessments of risk of bias due to missing results (arising from reporting biases) for each synthesis assessed	16
Certainty of evidence	#22	Present assessments of certainty (or confidence) in the body of evidence for each outcome assessed	6
**Discussion**			
Results in context	#23a	Provide a general interpretation of the results in the context of other evidence	15
Limitations of included studies	#23b	Discuss any limitations of the evidence included in the review	15
Limitations of the review methods	#23c	Discuss any limitations of the review processes used	16
Implications	#23d	Discuss implications of the results for practice, policy, and future research	16
**Other information**			
Registration and protocol	#24a	Provide registration information for the review, including register name and registration number, or state that the review was not registered	3
Registration and	#24b	Indicate where the review protocol can be accessed, or state that a	3

For peer review only

protocol		protocol was not prepared	
Registration and protocol	#24c	Describe and explain any amendments to information provided at registration or in the protocol	n/a
Support	#25	Describe sources of financial or non-financial support for the review, and the role of the funders or sponsors in the review	3
Competing interests	#26	Declare any competing interests of review authors	3
Availability of data, code, and other materials	#27	Report which of the following are publicly available and where they can be found: template data collection forms; data extracted from included studies; data used for all analyses; analytic code; any other materials used in the review	3

Notes:

- 6: Appendix 1
- 7: Appendix 1
- 17: Appendix 3 The PRISMA checklist is distributed under the terms of the Creative Commons Attribution License CC-BY. This checklist was completed on 13. July 2022 using https://www.goodreports.org/, a tool made by the EQUATOR Network in collaboration with Penelope.ai

For peer review only
